# Continuous positive airway pressure improves gait control in severe obstructive sleep apnoea: A prospective study

**DOI:** 10.1371/journal.pone.0192442

**Published:** 2018-02-23

**Authors:** Sébastien Baillieul, Bernard Wuyam, Jean-Louis Pépin, Mathieu Marillier, Renaud Tamisier, Dominic Pérennou, Samuel Verges

**Affiliations:** 1 HP2 laboratory, Grenoble Alpes University, Grenoble, France; 2 U1042, INSERM, Grenoble, France; 3 Pôle Thorax et Vaisseaux, Grenoble Alpes University Hospital, Grenoble, France; 4 LPNC laboratory (UMPR CNRS 5105), Grenoble Alpes University, Grenoble, France; 5 MPR Department, Grenoble Alpes University Hospital, Grenoble, France; AUSTRALIA

## Abstract

**Study aim:**

Severe obstructive sleep apnoea (OSA) can lead to neurocognitive alterations, including gait impairments. The beneficial effects of continuous positive airway pressure (CPAP) on improving excessive daytime sleepiness and daily functioning have been documented. However, a demonstration of CPAP treatment efficacy on gait control is still lacking. This study aims to test the hypothesis that CPAP improves gait control in severe OSA patients.

**Material and methods:**

In this prospective controlled study, twelve severe OSA patients (age = 57.2±8.9 years, body mass index = 27.4±3.1 kg·m^-2^, apnoea-hypopnoea index = 46.3±11.7 events·h^-1^) and 10 healthy matched subjects were included. Overground gait parameters were recorded at spontaneous speed and stride time variability, a clinical marker of gait control, was calculated. To assess the role of executive functions in gait and postural control, a dual-task paradigm was applied using a Stroop test as secondary cognitive task. All assessments were performed before and after 8 weeks of CPAP treatment.

**Results:**

Before CPAP treatment, OSA patients had significantly larger stride time variability (3.1±1.1% vs 2.1±0.5%) and lower cognitive performances under dual task compared to controls. After CPAP treatment, stride time variability was significantly improved and no longer different compared to controls. Cognitive performance under dual task also improved after CPAP treatment.

**Conclusion:**

Eight weeks of CPAP treatment improves gait control of severe OSA patients, suggesting morphological and functional cerebral improvements. Our data provide a rationale for further mechanistic studies and the use of gait as a biomarker of OSA brain consequences.

## Introduction

Obstructive sleep apnoea (OSA) is a highly prevalent, chronic disease, which is now widely accepted as a growing health concern [1]. Alterations in sleep quality, oxidative stress and neuro-inflammation triggered by chronic intermittent hypoxia along with changes in cerebrovascular reactivity with impaired cerebral blood flow [2, 3] contribute to the structural and functional cerebral changes associated with OSA [4, 5]. Neuropsychological domains of attention, memory and executive functions (EF) are specifically affected by OSA [6–9] with a substantial impact on daily functioning, work performance and productivity. The beneficial effects of continuous positive airway pressure (CPAP, the gold standard treatment for OSA) on improving excessive daytime sleepiness and daily functioning have been documented [10].

Mostly automatically piloted, gait may also be a demanding cognitive task, requiring attention and EF resources [11]. Stride time variability (STV) has been identified as a biomarker of gait control requiring cerebral integrity [12, 13]. Greater STV reflects worse gait control and is related to disease severity in disabling neurological conditions [14–16]. Two recent studies reported gait impairments in severe OSA. Celle et al. [17], in a cross-sectional study showed an association between moderate-to-severe OSA and greater (i.e., worse) STV. Allali et al. [18] in a prospective open-labelled study conducted in severe OSA participants reported that gait speed, step and stance time improved after 8 weeks of CPAP treatment, especially when gait was assessed while performing concurrently a cognitively demanding task. The lack of an appropriate control group, matched for age and anthropometric parameters makes the interpretation of the association between OSA and gait control impairments difficult. Moreover, evaluating the effect of CPAP on gait control is required and should demonstrate the relationship between OSA and gait control.

To test the hypothesis that gait control in severe non-obese OSA participants would be improved by CPAP treatment, we compared gait control assessed by STV in severe non-obese OSA participants before and after 8 weeks of CPAP treatment versus matched healthy subjects. To further characterize the mechanisms underlying gait impairments in OSA participants, gait and postural control have been assessed using a dual-task paradigm [11].

## Materials and methods

### Subjects

**Fig 1:**
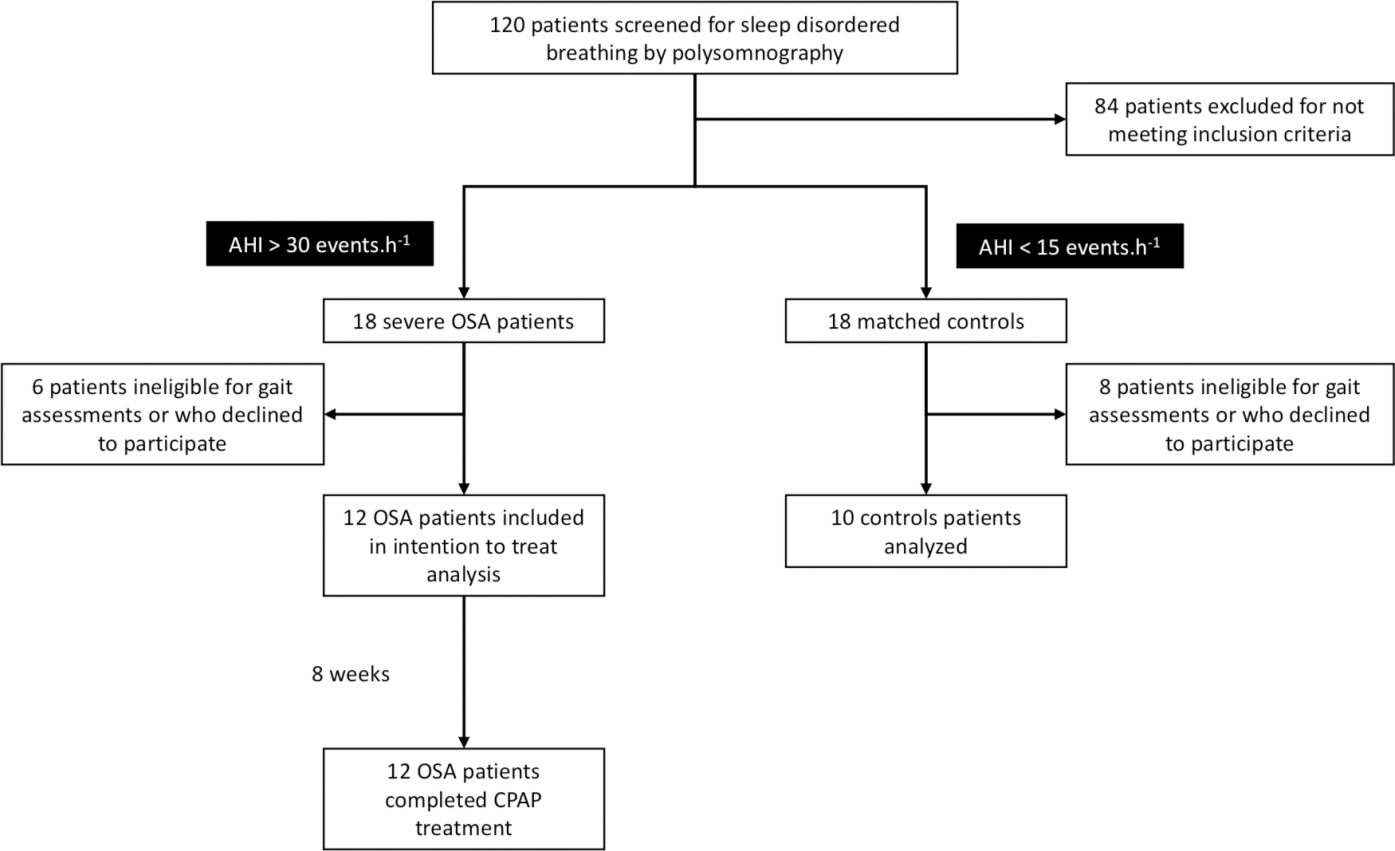
Study flow chart. AHI: Apnoea-Hypopnea Index; CPAP: Continuous Positive Airway Pressure treatment; OSA: Obstructive Sleep Apnoea.

Twelve severe, newly diagnosed OSA participants were recruited in this prospective controlled study conducted in the Sleep Laboratory of Grenoble-Alpes University Hospital between February 2014 and April 2016. Inclusion criteria were: (1) age ≥18 years and <70 years, (2) severe OSA syndrome (apnoea-hypopnoea index, AHI>30 events·hour^-1^) and (3) to present a strictly normal neurological examination. OSA diagnosis was based on a full-night polysomnography [19]. Ten healthy participants, matched for age and BMI were included as control group. They underwent a full-night polysomnography similar to OSA participants. Patients were excluded if they declined to participate or were unable to give informed consent. Patients with any of the following criteria were also excluded: the presence of any neurological, orthopaedic, visual or inner ear disease, any hypnotic or central nervous system targeted medication and chronic alcohol consumption (Fig 1).

The study was approved by local ethic committee (CPP Sud-Est V, 12-CHUG-12, ID RCB: 2012-A00A58-35, date of initial agreement: April 4^th^, 2012; date for the gait and posture investigations amendment agreement: January 21^st^, 2014 (S1 File). For more details on Study Protocol and ethical authorization, please refer to S1 and S2 Protocols). This study protocol is registered on ClinicalTrials.gov, with the following ID: NCT02854280. Study registration was performed after enrolment of participants started due to pending supplementary amendments at the time of first enrolments. The authors confirm that all ongoing and related trials for this intervention are registered. All participants gave their written informed consent prior to their participation in the study. Individuals photographed in the figures in this manuscript are associated with a written informed consent provided by the patient (as outlined in the PLOS consent form) to publish these case details.

### Polysomnography

Polysomnographic recordings were undertaken with electroencephalography (EEG) electrode positions C3/A2-C4/A1-Cz/01 of the international 10–20 Electrode Placement System, eye movements, chin electromyogram and ECG with a modified V2 lead. Sleep was scored manually according to standard criteria. Airflow was measured with nasal pressure prongs, together with the sum of oral and nasal thermistor signals. Respiratory effort was monitored using abdominal and thoracic bands. Oxygen saturation was measured using a pulse oximeter. An apnoea was defined as the complete cessation of airflow for at least 10 s and hypopnoea as a reduction of at least 50% in the nasal pressure signal or a decrease of between 30% and 50% associated with either oxygen desaturation of at least 3% or an EEG arousal, both lasting for at least 10 s [20]. Apnoea was classified as obstructive, central or mixed, according to the presence or absence of respiratory efforts. The classification of hypopnoea as obstructive or central was based on the thoraco-abdominal band signal and the shape of the respiratory nasal pressure curve (flow limited aspect or not). The AHI, defined as the number of apnoea and hypopnoea per hour of sleep was calculated.

### Experimental protocol

All the participants underwent the same protocol evaluation: (1) clinical examination and single task (SiT) cognitive performance assessment, (2) SiT overground gait, (3) treadmill gait assessment in SiT and dual task (DT) and (4) standing postural control in SiT and DT. All the assessments were interspersed by a resting period of at least 15 minutes. OSA participants were evaluated at baseline and after eight weeks of CPAP treatment while control participants were evaluated only once.

#### Dual-task paradigm

**Fig 2:**
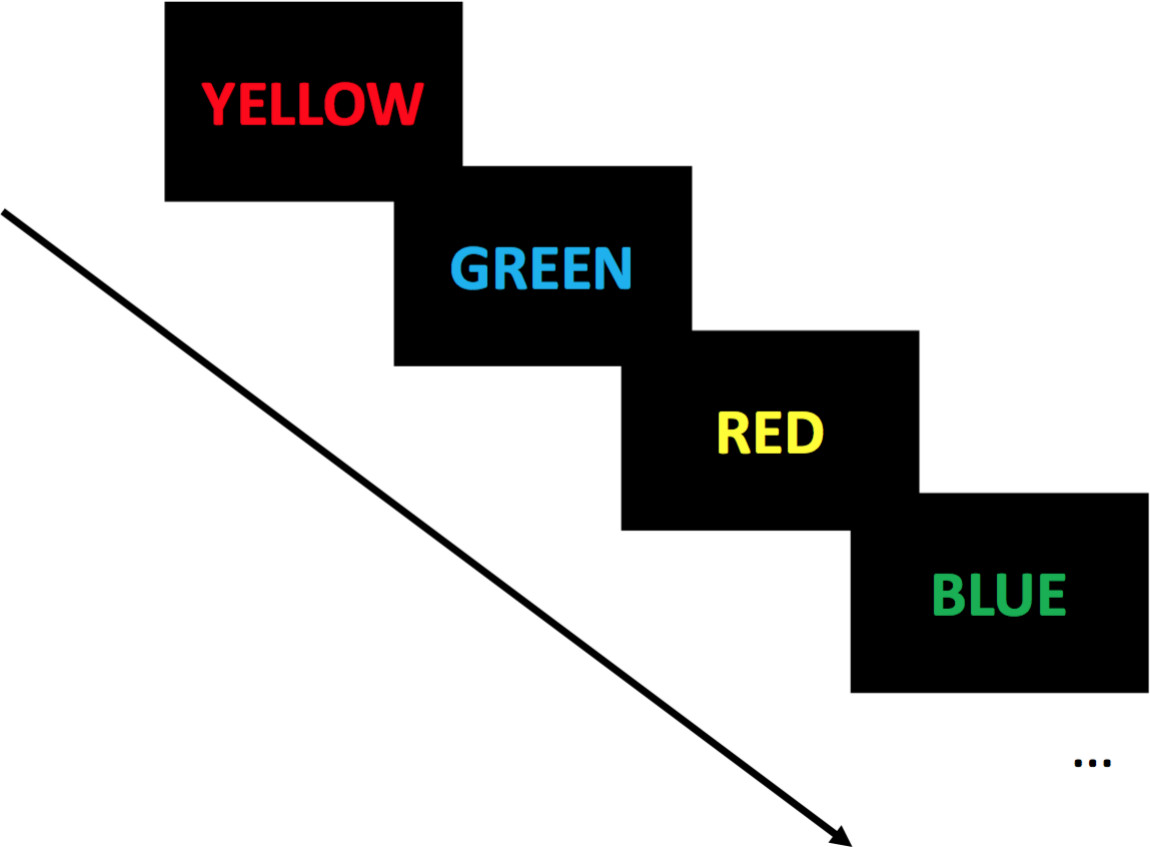
Schematic representation of the visuo-verbal Stroop test. Participants were instructed to name the words font colour and to inhibit reading the word (Correct answers here: “red” then “blue” then “yellow” then “green”). Words were presented one by one, and the evaluator skipped manually to the next one after the subject gave an oral response.

Dual-task paradigm consists in the assessment of the interferences occurring when a motor and a cognitive task are performed simultaneously [11]. The Stroop colour word interference test [21] was used as secondary task. Stroop colour test is a cognitive task considered to specifically measure selective attention and cognitive inhibition, two EF subdomains [22, 23]. Stroop test consists in colour names (blue, red, green and yellow) written in a different font colour. Participants were instructed to name the words font colour and to inhibit reading the word (e.g., the word “red” written in yellow font colour (Fig 2). To avoid learning effects, there were 10 different versions of the Stroop test presented randomly to the participants throughout the different assessments. Each version consisted of 30 colour words. For all single task (SiT) and dual-task (DT) assessments, the Stroop test was displayed on a black background screen. Screen was systematically installed 1.5 m ahead of the participant and its height adjusted for each participant and for each evaluation. Words were presented one by one, and the evaluator skipped manually to the next one after the subject gave an oral response. The number of correct answers and errors were recorded by a trained evaluator (SB). In DT, participants were asked to perform the two tasks at the best of their capacity without any task prioritization. The correct response rate (Correct response rate = Response rate per second × Percentage of correct responses) accounted for EF performance in DT gait and posture assessments [24].

#### Clinical and cognitive assessment

Prior to all gait and postural assessments, participants underwent a screening history and physical examination to ensure that they were free of significant orthopaedic, neurological and visual disorder which could interfere with the outcomes of the present study. Subjects were tested for their ability to distinguish colours appropriately by using the same screen setting as during the dual-task assessments (S1 Fig). The four colours used in our Stroop test were consecutively displayed on the screen (S1A Fig), then words written in the congruent font colour were displayed (S1B Fig). Participants were instructed to give the right colour name (S1A Fig) and to name the words font colour (S1B Fig). Then, all participants then performed one Stroop test in a sitting position to assess their SiT performance regarding EF. Daytime sleepiness was assessed with the Epworth Sleepiness Scale [25].

#### Overground gait and stride time variability assessment

**Fig 3:**
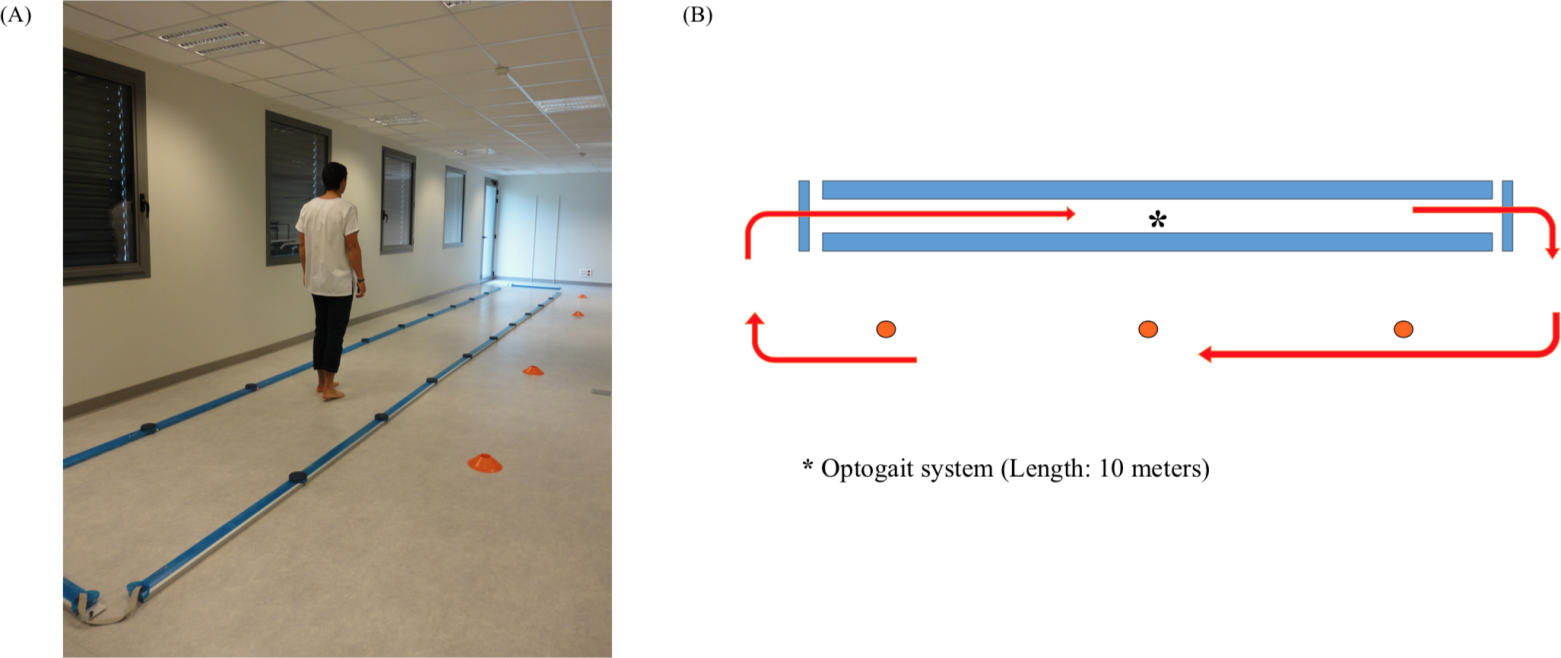
Overground gait assessment. (A) Experimental setting. (B) Schematic representation of the oval gait circuit.

Spatiotemporal gait parameters were recorded using a 10-meter long OptoGait system (OptoGait, Microgate, Bolzano, Italy), which demonstrated high reliability for the assessment of spatiotemporal gait parameters [26]. Participants walked bare foot, at their self-selected, comfortable speed continuously along an oval circuit [27] (Fig 3). Each participant completed three familiarization and five consecutive evaluation loops. Gait speed, step frequency, 2 spatial (stride length, step width) and 1 temporal (stride time) gait parameters were analysed. Data were recorded at a 1 kHz sample frequency and analysed using the OptoGait software (version 1.10.7.0, Microgate). The average value for all recorded steps was used for data analysis. STV, our primary study endpoint, was calculated by the mean of the coefficient of variation of stride time (CV) [16].

CVofstridetime=StandardDeviationofstridetime/Meanstridetime×100

#### Treadmill dual-task gait

**Fig 4:**
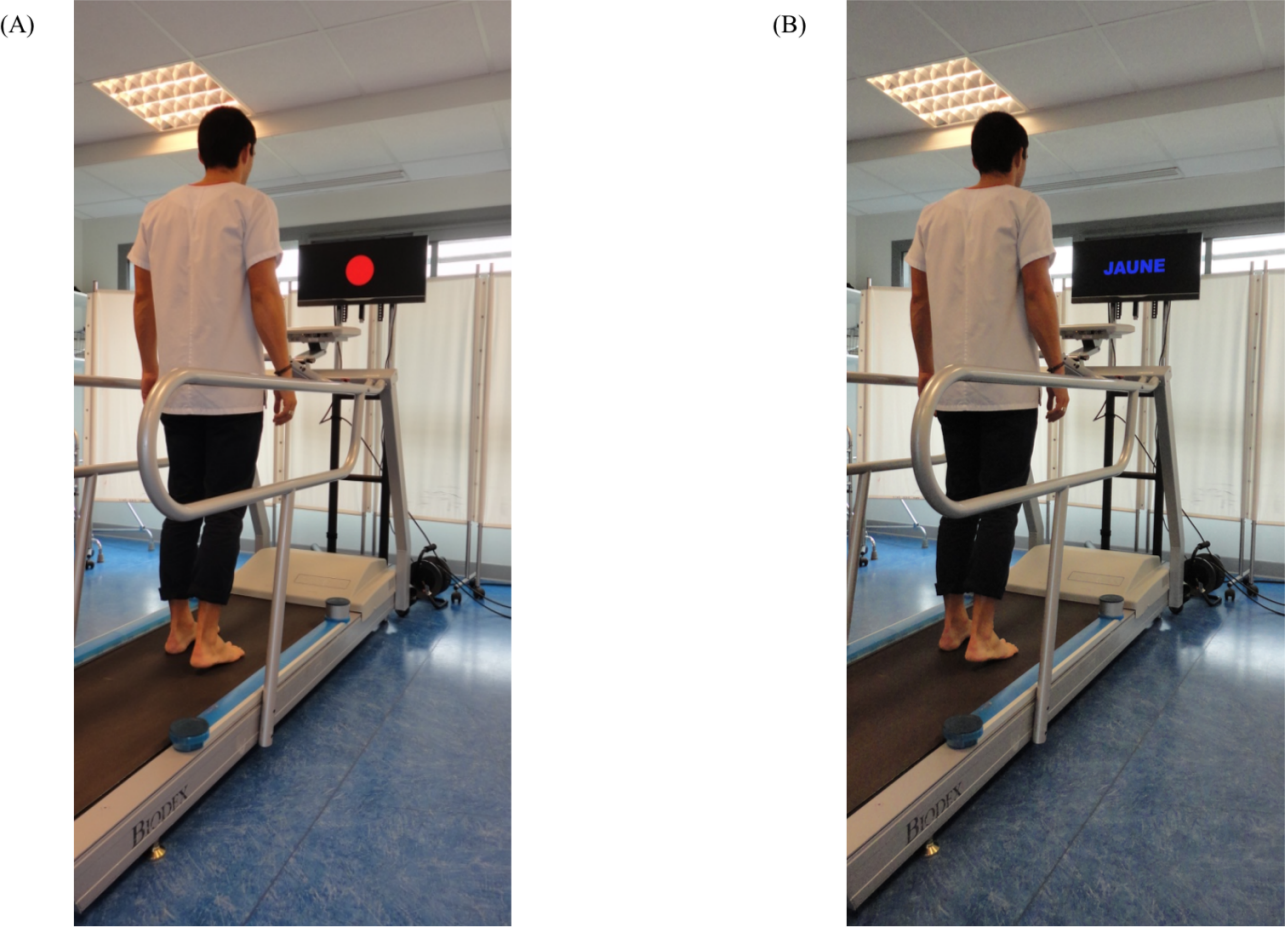
Treadmill gait assessment. (A) Experimental setting in single task (gait only). (B) Experimental setting in dual task (Gait and Stroop test).

To ensure the recording of a higher number of steps, DT gait assessments were performed on a treadmill (Gait Trainer 3, Biodex Medical System, NY, USA) (Fig 4). Spatiotemporal gait parameters were recorded using an OptoGait system (Microgate). Following a 10-minute period of habituation to the treadmill [28], each participant’s preferred walking speed was determined according to a standardized protocol [29]. In OSA participants, the preferred walking speed determined before CPAP treatment was used for post-CPAP evaluations. To evaluate the influence of speed on OSA patients’ ability to walk under DT condition, gait assessments were performed in two conditions of speed (preferred walking speed and preferred walking speed+30%), alternatively in SiT (gait only) and in DT (gait and Stroop test). Each trial lasted 30 s with 4 trials per condition (2 gait*2 task paradigms, 16 trials in total). Participants walked continuously during 30 s between each trial. In SIT, participants were instructed to walk according to their natural pattern, arms moving freely by their sides, while looking straight-ahead at a fixed red target displayed on the screen. In DT, subjects were asked to walk as naturally as possible and to perform the Stroop test at the best of their capacity without any task prioritization. STV was calculated.

#### Standing postural control

**Fig 5:**
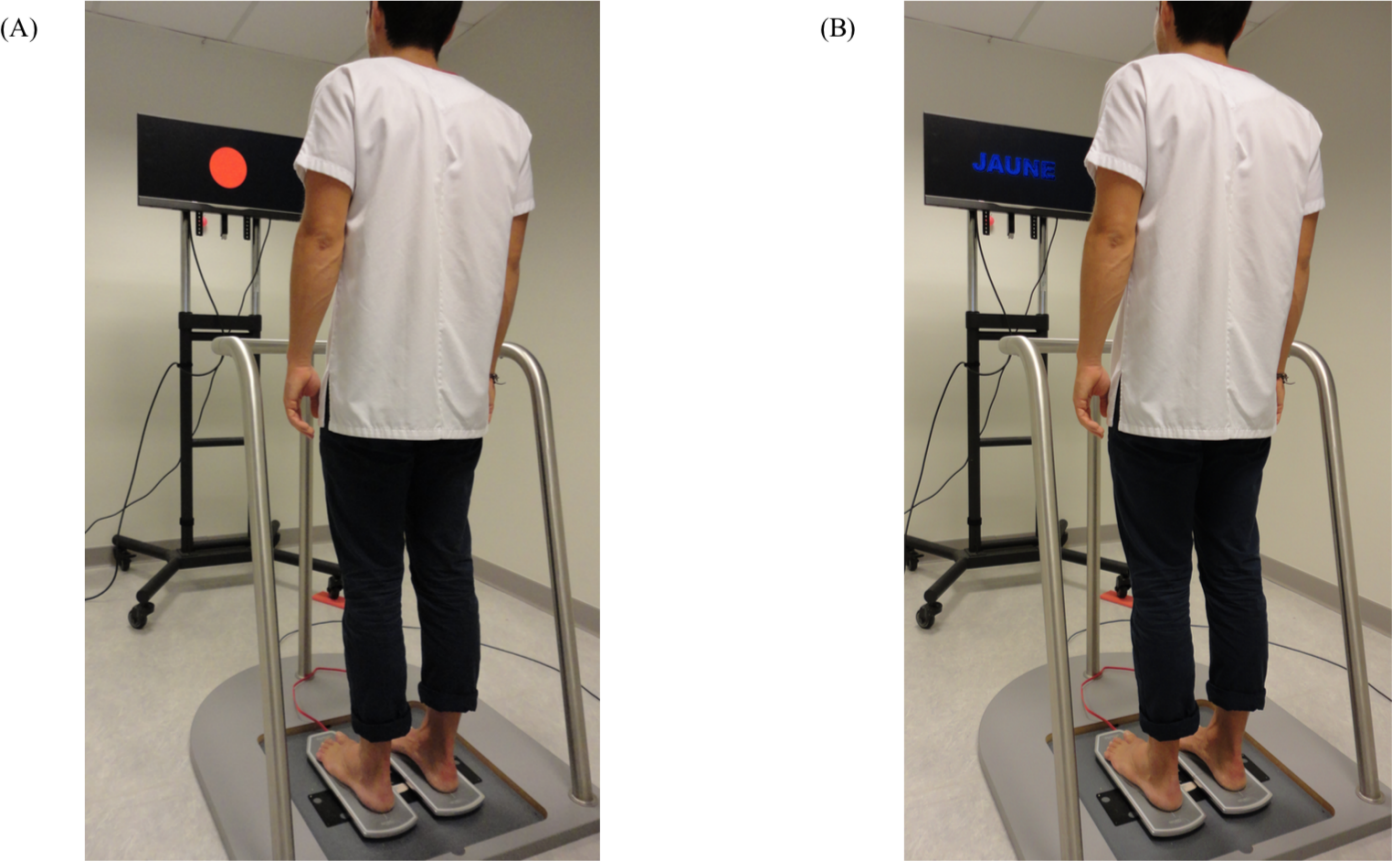
Standing postural control assessment. (A) Experimental setting in single task (gait only). (B) Experimental setting in dual task (Gait and Stroop test).

As an efficient postural control is a determinant of stride-to-stride gait variability [16], we assessed standing postural control using a posturographic platform (Feetest 6, TechnoConcept, Céreste, France). The two dynamometric posturographic clogs were positioned in a parallel manner, with a 4-cm width. Participants stood upright barefoot on the clogs, with their arms alongside the body. Assessments were alternatively performed in SiT (posture only, 4 trials) and in DT (posture and Stroop test, 4 trials) (Fig 5). Each trial lasted 30 seconds (1 postural*2 task paradigms, 16 trials in total). One minute of rest sitting was systematic between trials. Data were recorded with a sampling rate of 40 Hz, and calculated using the Posturewin 4 software.

In SiT, subjects were instructed to maintain their balance while looking straight-ahead at a fixed red target displayed on the screen. In DT, subjects were asked to maintain the erect posture as still as possible and to perform the Stroop test at the best of their capacity without any task prioritization. As a marker of an efficient standing postural control, the amount of sway was assessed by calculating centre of pressure (CoP) area (90% confidence ellipse, mm^2^). The smaller the area, the better the postural control [30].

#### Continuous positive airway pressure treatment

CPAP treatment was applied with an auto-titrating machine (Autoset Spirit, ResMed, UK or Remstar Auto, Philips Respironics, Murrysville, PA, USA) provided by a home care company (Agir à Dom, France). CPAP compliance and AHI_Flow_ [31] were measured from the machine’s internal microprocessor.

### Data and statistical analysis

This is an ancillary study of a large project, which aim was to investigate the effects of OSA on the central neuromuscular mechanisms of fatigue, before and after 8 weeks of CPAP treatment.

The reported variables are the averaged values from the trials performed in each condition. All variables are reported as mean ± one standard deviation (SD), or mean [95% confidence interval for mean] when appropriate. Normality of distributions and homogeneity of variances were confirmed using the Kolmogorov-Smirnov and Skewness test, respectively. Analyses were performed in an “intention to treat” manner. Between-group comparisons were performed using t-tests for independent samples while pre- and post-CPAP comparisons in OSA group were conducted using paired-sample t-tests. To assess the effect of group and task paradigm (SiT *versus* DT) at baseline, variables were analysed using two-way ANOVAs (group×task paradigm). To assess the effect of treatment and task paradigm in OSA group, variables were analysed using two-way ANOVAs with repeated measures (treatment×task paradigm). Tukey’s test for post-hoc analysis was used if ANOVA indicated a significant main effect or interaction. Partial eta square (_p_η^2^) values are reported as measures of effect size, with moderate and large effects considered for _p_η^2^≥0.07 and _p_η^2^≥0.14, respectively [32]. A two-tailed α level of 0.05 was used as the cut-off for significance. Statistical analyses were carried out using IBM’s Statistical Package for the Social Sciences (SPSS), Version 23.0.

## Results

### Clinical characteristics

No difference was found for age and anthropometric parameters as well as for daytime sleepiness levels assessed by the Epworth Sleepiness Scale between OSA and controls (Table 1). OSA participants showed no difference in baseline SiT EF performance compared to controls (percentage of correct response at Stroop test in SiT: 96.9±2.9% vs. 98.2±1.3%, respectively, *p*>0.05).

**Table 1:** Anthropometric and apnoea parameters for control group (CONTROLS) and obstructive sleep apnoea (OSA) group.

		CONTROLS (N = 10)	OSA (N = 12)	p
Anthropometric				
	Sex	8 ♂/ 2 ♀	11 ♂/ 1 ♀	0.57 ^a^
	Age (years)	60.2 ± 7.6	57.2 ± 8.9	0.41
	BMI (kg·m^-2^)	25.1 ± 3.2	27.4 ± 3.1	0.10
Epworth Sleepiness Scale score				
		6.90 ± 3.14	9.83 ± 5.02	0.13
Apnoea characteristics and CPAP observance				
	AHI (events·hour^-1^)	8.1 ± 6.9	44.0 ± 12.7	< 0.001
	AHI_Flow_ (events·hour^-1^)	-	2.9 ± 3.1	
	Percentage of nights with CPAP usage (%)	-	86.1 ± 18.7	
	Percentage of nights with CPAP usage > 4h/night (%)	-	65.7 ± 35.1	
	Mean duration of CPAP utilization (hours·night^-1^)	-	4.8 ± 2.1	
	Peff (cmH_2_O)	-	8.1 ± 1.8	

Data are mean values ± SD. Between-group comparisons were performed using t-tests for independent samples except for

^a^, calculated using a Fisher exact test.

AHI: Apnoea-Hypopnoea Index; AHI_Flow_: residual AHI on CPAP; BMI: Body Mass Index; CPAP: Continuous Positive Airway Pressure; OSA: Obstructive Sleep Apnoea group; Peff: Effective pressure.

### Baseline assessment

#### Overground gait and stride time variability assessment

**Fig 6:**
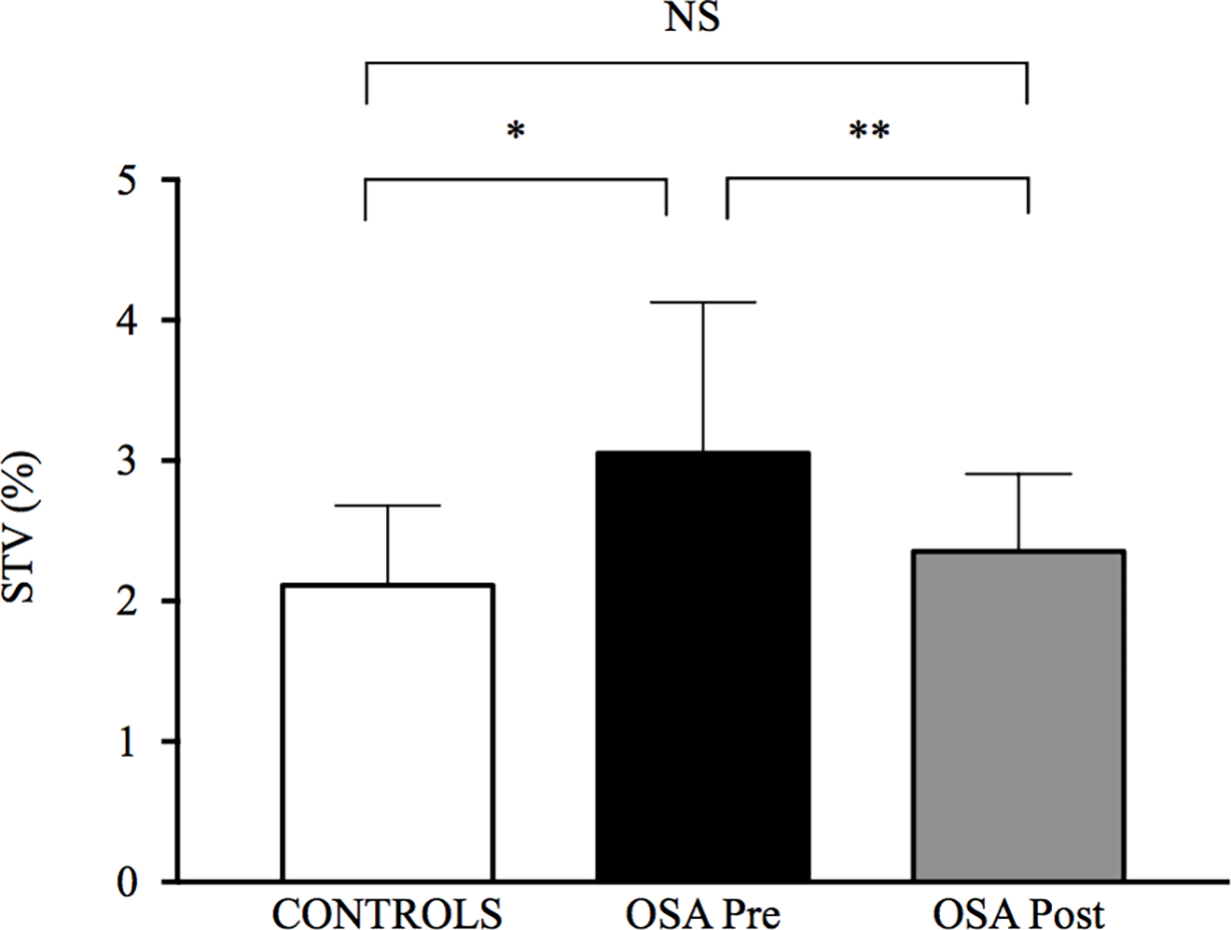
Stride time variability (STV) in control and in obstructive sleep apnoea (OSA) groups before and after 8 weeks of continuous positive airway pressure treatment. Values are presented as mean ± SD. Between-group comparisons were performed using t-tests for independent samples. Pre- and post-CPAP comparisons in OSA group were conducted using paired-sample t-tests.

Spatiotemporal gait parameters are presented in Table 2. OSA participants showed larger step width at baseline compared to controls (*p* = 0.001, _p_ƞ^2^ = 0.46). OSA participants showed higher STV compared to controls at baseline (*p* = 0.02, _p_ƞ^2^ = 0.24; Fig 6).

**Table 2:** Spatiotemporal gait parameters recorded overground. Spatiotemporal gait parameters data are presented for control group (CONTROLS) and for the obstructive sleep apnoea group (OSA) before (Pre) and after eight weeks of continuous positive airway pressure treatment (Post).

Gait parameters	CONTROLS	OSA	
		Pre	Post
Speed (m·s^-1^)	1.2 [1.1; 1.2]	1.2 [1.1; 1.3]	1.1 [1.1; 1.2]
Frequency (steps·min^-1^)	108.0 [104.0; 111.9]	108.9 [101.5; 116.3]	105.5 [99.9; 111.1]
Stride length (cm)	128.6 [119.7; 137.5]	134.7 [124.4; 145.0]	130.0 [122.4; 137.7]
Step width (cm)	12.3 [11.3; 13.2]	16.1 [14.2; 18.0] ***	15.5 [14.1; 17.0] ***
Stride time (s)	1.1 [1.1; 1.2]	1.1 [1.0; 1.2]	1.2 [1.1; 1.2]

Data are mean values [95% confidence interval for mean]. Between-group comparisons were performed using t-tests for independent samples. Pre- and post-CPAP comparisons in OSA group were conducted using paired-sample t-tests. OSA: Obstructive Sleep Apnoea group.

*** significantly different compared to CONTROLS (*p* = 0.001).

#### Treadmill gait

**Fig 7:**
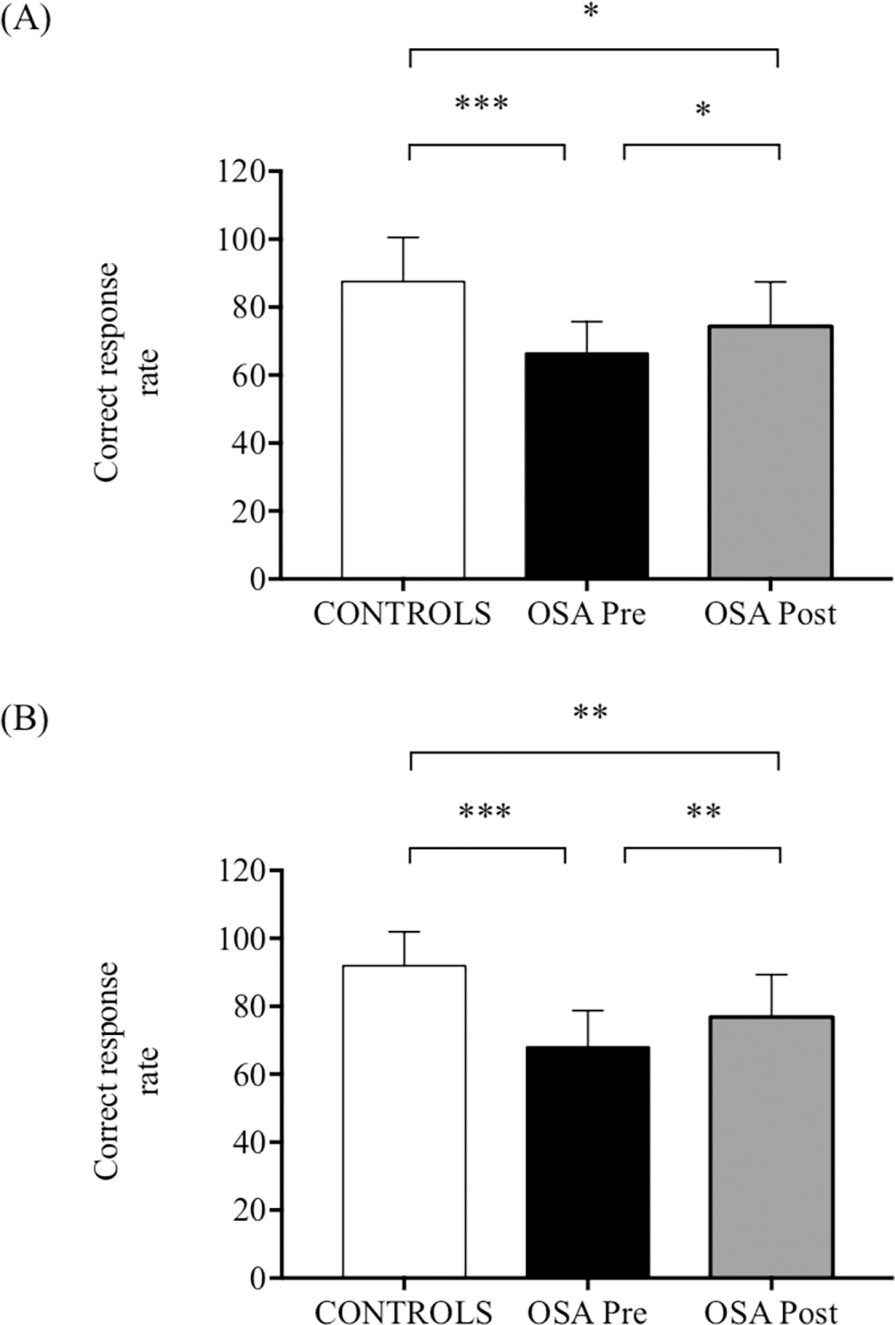
Stroop test performance under dual-task condition while walking on a treadmill. Stroop test performance was assessed by the correct response rate in control and in obstructive sleep apnoea (OSA) groups before and after 8 weeks of continuous positive airway pressure treatment. (A) At preferred walking speed. **(**B) At preferred walking speed+30%. Values are presented as mean ± SD. Between-group comparisons were performed using t-tests for independent samples. Pre- and post-CPAP comparisons in OSA group were conducted using paired-sample t-tests.

OSA participants spontaneously choose a significantly higher preferred walking speed compared to controls (1.1±0.3 m·s^-1^ versus 0.8±0.1 m·s^-1^, *p* = 0.004, _p_ƞ^2^ = 0.39). Neither significant group main effect nor group×task paradigm interaction was observed for STV both at preferred walking speed (respectively *p* = 0.60, _p_ƞ^2^ = 0.008 and *p* = 0.32, _p_ƞ^2^ = 0.026) and at preferred walking speed+30% (respectively *p* = 0.65, _p_ƞ^2^ = 0.006 and *p* = 0.73, _p_ƞ^2^ = 0.003) (Table 3). OSA participants showed lower correct response rate during DT compared to control at preferred walking speed (*p*<0.001, _p_ƞ^2^ = 0.46) (Fig 7A) and preferred walking speed+30% (*p*<0.001, _p_ƞ^2^ = 0.58) (Fig 7B).

**Table 3:** Stride time variability (STV) evaluation during treadmill gait assessment. Results are presented for control group (CONTROLS) and for obstructive sleep apnoea (OSA) group before (Pre) and after (Post) eight weeks of continuous positive airway pressure treatment. Evaluations were performed while walking on the treadmill only (single task) and while walking on the treadmill and simultaneously performing Stroop test (double task) at preferred walking speed and preferred walking speed+30%.

Conditions	CONTROLS	OSA	
		Pre	Post
Preferred walking speed			
Single task	2.6 [2.0; 3.2]	3.4 [1.6; 4.9]	2.5 [1.5; 3.6]
Dual task	3.8 [2.1; 5.4]	4.5 [0.9; 7.5]	3.4 [1.1; 5.7]
Preferred walking speed + 30%			
Single task	2.1 [1.3; 2.9]	2.3 [1.6; 2.7]	1.9 [1.4; 2.5]
Dual task	2.2 [1.6; 2.8]	2.0 [1.4; 2.3]	1.9 [1.4; 2.4]

Data are mean values [95% confidence interval for mean]. Between-group comparisons were performed using t-tests for independent samples. Pre- and post-CPAP comparisons in OSA group were conducted using paired-sample t-tests. OSA: Obstructive Sleep Apnoea group.

#### Standing postural control

**Fig 8:**
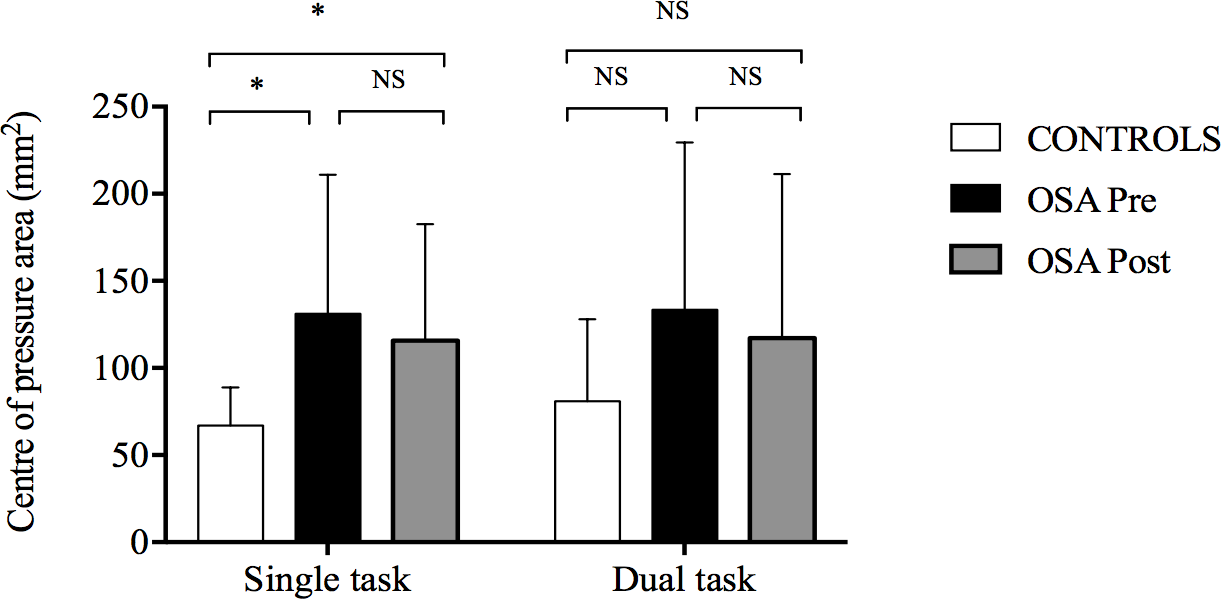
Changes in centre of pressure area. Centre of pressure data are presented for control and for obstructive sleep apnoea (OSA) groups before and after 8 weeks of continuous positive airway pressure treatment. while performing posture alone (single task) and while performing simultaneously posture and Stroop test. Values are presented as mean ± SD. Between-group comparisons were performed using t-tests for independent samples. Pre- and post-CPAP comparisons in OSA group were conducted using paired-sample t-tests.

**Fig 9:**
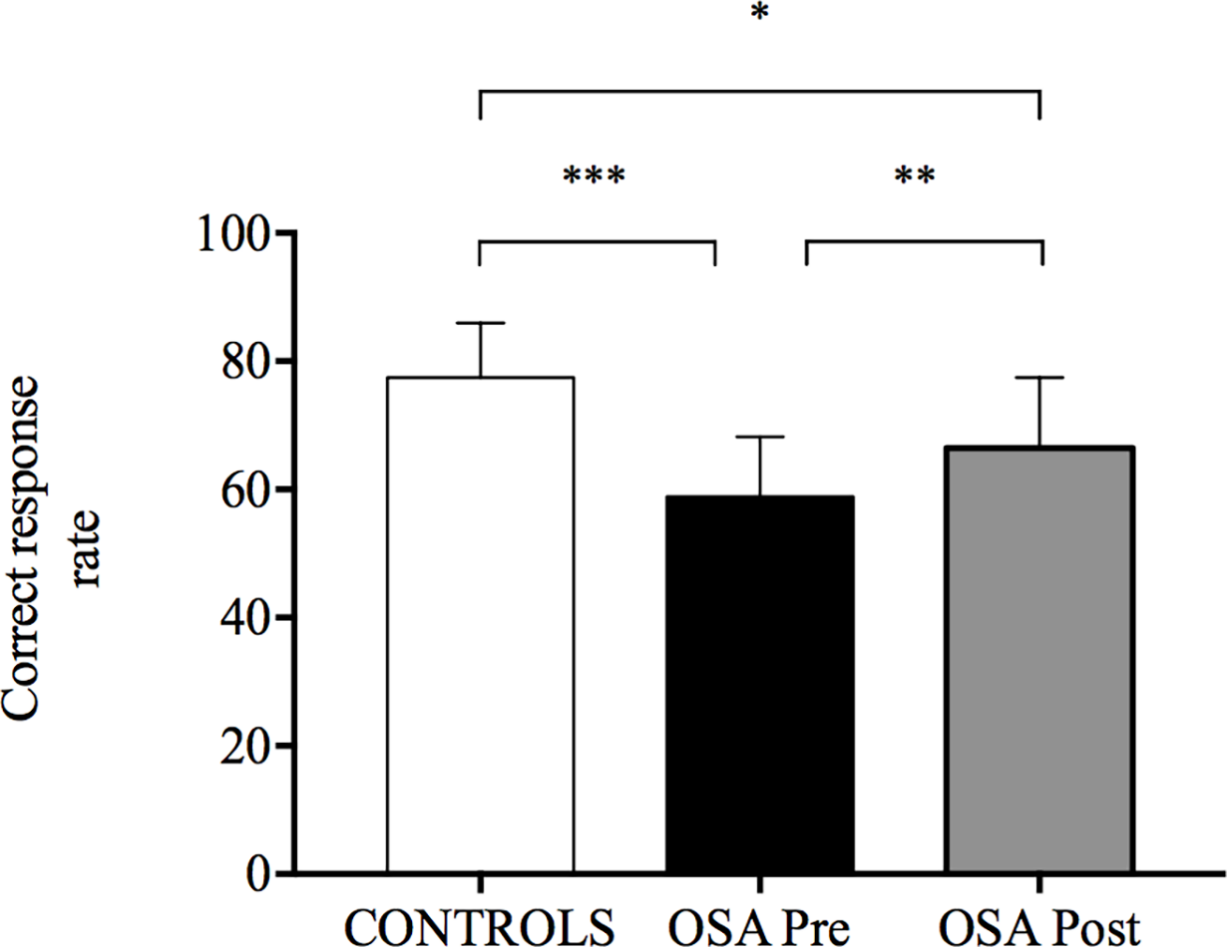
Stroop test performance under postural dual-task condition. Stroop test performance was assessed by the correct response rate in control and in obstructive sleep apnoea (OSA) groups before and after 8 weeks of continuous positive airway pressure. Values are presented as mean ± SD. Between-group comparisons were performed using t-tests for independent samples. Pre- and post-CPAP comparisons in OSA group were conducted using paired-sample t-tests.

A significant group effect was observed for CoP area (F(2, 40) = 7.57, *p* = 0.01, _p_ƞ^2^ = 0.16) without significant interaction between group and task paradigm (F(2, 40) = 0.08, *p* = 0.78, _p_ƞ^2^ = 0.002). OSA participants presented a larger CoP area compared to controls (Fig 8). OSA participants showed decreased correct response rate during DT compared to controls (*p*<0.001, _p_ƞ^2^ = 0.54) (Fig 9).

### Impact of CPAP treatment

#### Clinical characteristics

CPAP treatment induced no significant change in BMI (post-CPAP BMI = 27.5 ± 3.2 kg·m^-2^, *p* = 0.64). CPAP treatment improved significantly daytime sleepiness in OSA patients, with a mean Epworth Sleepiness Scale score post-CPAP treatment of 5.2 ± 2.7 (p = 0.007).

#### Overground gait and stride time variability assessment

CPAP treatment had no significant effect on spatiotemporal gait parameters (Table 2). In contrast, STV was significantly improved in OSA participants (*p* = 0.005, _p_ƞ^2^ = 0.52) and did not differ anymore compared to controls (*p* = 0.32, _p_ƞ^2^ = 0.05; Fig 6).

#### Treadmill gait

Neither treatment main effect nor treatment × task paradigm interaction was observed for STV both at preferred walking speed (respectively *p* = 0.16, _p_ƞ^2^ = 0.19 and *p* = 0.24, _p_ƞ^2^ = 0.14) and preferred walking speed+30% (respectively *p* = 0.26, _p_ƞ^2^ = 0.12 and *p* = 0.32, _p_ƞ^2^ = 0.10) (Table 3). OSA participants presented significant improvements in correct response rate during DT after CPAP treatment both at preferred walking speed (*p* = 0.01, _p_ƞ^2^ = 0.46) and at preferred walking speed+30% (*p* = 0.003, _p_ƞ^2^ = 0.58) but their performances remained significantly different compared to controls (*p* = 0.04, _p_ƞ^2^ = 0.20 and *p* = 0.01, _p_ƞ^2^ = 0.30, respectively) (Fig 7A and 7B).

#### Standing postural control

Neither treatment main effect nor treatment×task paradigm interaction was observed for CoP area (respectively *p* = 0.31, _p_ƞ^2^ = 0.095 and *p* = 0.92, _p_ƞ^2^ = 0.001) after treatment compared to baseline (Fig 8). OSA participants presented significant improvement in correct response rate during DT after treatment (*p* = 0.03, _p_ƞ^2^ = 0.36), but their performances remained significantly different compared to controls (*p* = 0.02, _p_ƞ^2^ = 0.25) (Fig 9).

## Discussion

This prospective, controlled study confirms the presence of gait control alterations [17, 18] in severe OSA patients. This study shows for the first time that gait control was significantly improved after 8 weeks of CPAP treatment. The originality of our study was to assess conjointly gait, postural control and EF performance, which were both altered in OSA patients compared to controls and partially normalized by treatment.

### Gait improvement following CPAP treatment: A brain-centred hypothesis

We reported a higher STV in our group of severe, mostly over-weight OSA participants before CPAP treatment compared to matched controls (Fig 6), suggesting impaired gait control. This result is consistent with a recent study that reported a positive correlation between OSA severity and STV [17]. If most of the mean spatiotemporal parameters did not differ between the two groups, our study report a significantly larger step width in OSA patients compared to controls (Table 2). Increased step width is a meaningful gait impairment occurring in many neurological conditions [33, 34]. Such an adaptive strategy is described as aiming to stabilize gait when subjects perceive a challenge to their balance [35]. We compared OSA participants to controls matched for age and BMI, two major factors of gait disturbances [36, 37]. Subjects performed five times a circuit designed to avoid perturbations induced by gait initiation or termination, which makes the assessment of spatiotemporal gait parameters and STV more sensitive and reliable [38]. Key cerebral regions, functionally related to EF that regulate gait variability and the control of gait [39] (i.e. frontal brain regions) are sites of hypotrophic changes (i.e. grey matter reduction) in OSA [5]. Also, cerebral perfusion alterations may impair substrates and oxygen delivery to the brain, especially during brain activation associated with walking or cognitive tasks for instance [2, 3, 40].

Significant improvements of step time and stance time were described following 8 weeks of CPAP in moderate-to-severe OSA patients [18]. This study however did not investigate the impact of CPAP on gait control impairments evaluated by STV, as suggested by Celle et al. [17]. Our results demonstrate a significant improvement of STV following 8 weeks of CPAP treatment which returns to control values. This improvement was associated with a reduction in daytime sleepiness but with no change in BMI. The role of such a moderate reduction of daytime sleepiness (mean reduction of Epworth Sleepiness Scale score = 4.67 ± 4.64) regarding the improvement in gait control is questionable. As suggested by Allali et al., metabolic and morphological brain changes described in the frontal lobes of OSA patients and their modification after CPAP treatment could contribute to the improvement of temporal gait parameters after CPAP treatment [18]. A significant increase in cerebrovascular reactivity [3] as well as white [41] and grey matter changes positively related to improvements in cognitive sub-domains [42] have been described following CPAP treatment. Hence, cerebrovascular and neuroanatomical changes induced by CPAP might underlie gait control improvements observed in the present study. From a clinical and pathophysiological point of view, it would have been informative to assess gait control according to patient phenotypes, e.g. depending on the severity of hypoxemia at baseline. Also, comparing patients according to their CPAP treatment compliance would have strengthened our interpretation of CPAP effect. However, due to our small sample size and the lack of statistical power, we were not able to investigate rigorously those aspects.

### Dual task performance

OSA participants and controls did not differ regarding STV in the two conditions of treadmill speed. Gait assessments in DT were performed on a treadmill to record a greater number of steps. This choice has probably limited the stride-to-stride variability by influencing the temporal rhythm of gait [38]. Nevertheless, OSA patients choose a greater preferred walking speed than controls. Higher gait speeds have been previously reported as being associated with a more coordinated walking pattern [43]. This suggests that OSA patients may have chosen higher gait speed on the treadmill to improve gait stability in such a challenging walking condition.

Our study evaluated conjointly gait and postural control in the same OSA group. Postural stability, as a marker of postural control efficiency, is a determinant of stride-to-stride gait variability [16]. Decreased postural performance was observed in SiT in OSA patients compared to controls, demonstrating an impaired postural stability, which is consistent with a previous cohort study [44].

DT paradigm is the most common method used to distinguish automatic and executive control of gait [11, 39]. Lower performances at the Stroop test in DT were observed in OSA participants compared to controls. This suggests that OSA participants were not able to perform as efficiently as the controls the two tasks simultaneously, gait and postural control being maintained during DT in OSA participants at the expense of EF performance. This supports the hypothesis of a “posture first” strategy that might have been spontaneously chosen by OSA participants [45] who may prioritize the motor task over the cognitive one to ensure safety while walking or standing.

DT performance essentially relies on the integrity of EF [18] suggesting impaired executive functioning in our group of OSA participants. Cognitive performance of OSA participants was at least partly improved by CPAP suggesting the persistence of an executive dysfunction even after two months of treatment and despite of the improvement of daytime sleepiness. This result is concordant with a recent study of our group that reported a partial effect of CPAP on cognitive impairments and in particular memory impairments, suggesting a complex OSA-neurocognitive relationship [46].An exhaustive neuropsychological examination of the patients both at baseline and after CPAP treatment is lacking in the present study to better understand the impact of the disease and of the treatment on cognitive and DT performances.

### Gait assessment in OSA syndrome: Clinical implications and perspectives

The present results may have several clinical implications. First, the recent description of gait and postural impairments in OSA encourages the development of OSA screening strategies in rehabilitation centres, especially in patients suffering from neurological diseases. Second, one major consequence of OSA is executive dysfunction which is insufficiently and subjectively assessed in clinical routine [5]. Further studies are needed to firmly validate the role of gait assessment as a potential objective biomarker of OSA-related subclinical brain injuries and their improvement following CPAP treatment.

### Limitations

The small sample size is the main limitation of this prospective study and could have caused limited power to detect significant interactions. However, the large effect sizes of the main results emphasize the significance of the differences observed between OSA patients and controls and from pre- to post-CPAP treatment. The two other major limitations of this study are the absence of an OSA group treated by sham-CPAP and the absence of a retest of the controls eight weeks after baseline assessment. These methodological limits restrict the strength of our interpretation of the effects of CPAP treatment. Further studies should consider the assessment of the effects of CPAP treatment on gait control in a randomized, controlled (effective vs. sham-CPAP), double-blind design. As we included only severe OSA participants, furthermore generalization of the study findings should be restricted to OSA patients with an AHI>30 events·hour^-1^.

Neuroimaging investigations associated with gait and cognitive assessments at baseline and their changes following CPAP treatment will be helpful in future studies to better understand the complex relationship between gait control and brain structural and functional alterations in OSA patients.

An 8-week CPAP treatment duration was implemented in the present study since previous studies showed that peripheral vascular and cerebrovascular functions are improved by CPAP following less than 8 weeks of treatment [47, 48]. Nevertheless, further studies should consider assessing the effects of CPAP treatment on gait control after longer exposures as a longer period of efficient CPAP therapy could have promote greater improvements in cognitive functioning [49].

## Conclusion

Using conjointly gait and postural assessments, our study confirms and extends the knowledge regarding gait impairments and CPAP efficacy in OSA. Our data provide a rationale for further mechanistic studies and the potential use of gait as a biomarker of OSA brain consequences.

## Supporting information

S1 Checklist TREND checklist. (PDF)

S1 Protocol Study protocol. Full length french version.
(PDF)

S2 Protocol Study protocol. English language summary.
(DOCX)

S1 File Ethical authorization. (PDF)

S1 Fig Pre-assessment visual testing, to ensure that subjects were able to properly distinguish colours by using the same screen setting as during the dual-task assessments. The four colours used in our Stroop test were consecutively displayed on the screen (A), then words written in the congruent font colour were displayed (B). Participants were instructed to give the right colour name (A) and to name the words font colour (B).
(TIF)
