# Supplementary material for: Continuous positive airway pressure improves gait control in severe obstructive sleep apnoea: A prospective study
Source: PLoS One. 2018 Feb 23;13(2):e0192442. doi: 10.1371/journal.pone.0192442 (PMC5825012; doi:10.1371/journal.pone.0192442)
Supplement: S1 Protocol — Full length french version. (PDF) [file pone.0192442.s002.pdf]

Le cerveau à l'effort : Effets de l'hypoxie chez le malade respiratoire

Titre court : NEUROX

Recherche clinique : Etude physiopathologique.

Personne autorisée à signer le protocole au nom du promoteur :

Mme Hélène SABBAH-GUILLAUME, Directrice de la Recherche et des Partenariats
Délégation à la Recherche Clinique et à l'Innovation

Promoteur :

CHU de Grenoble, BP 217, 38043 Grenoble Cedex 9.
Tél : 04-76-76-56 09 Fax : 04-76-76-52-21

Investigateur coordonnateur :

Pr. Patrick LEVY, Laboratoire d'Explorations Fonctionnelles Cardio-Respiratoires et Laboratoire du Sommeil, CHU de Grenoble. Tél : 04-76-76-55-16 Fax : 04-76-76-56-17
E-mail : plevy@chu-grenoble.fr
N° Conseil de l'Ordre : 38/03573
N° ADELI : 38 10 357 32

Investigateurs associés:

Dr. Bernard WUYAM, Pôle Rééducation & Physiologie, Clinique de Physiologie Sommeil, Exercice, UM Médecine du Sport & des Activités Physiques, Recherche sur l'exercice & Réhabilitation, CHU de Grenoble. Tél : 04-76-76-92-11 Fax : 04-76-76-56-17 E-mail : bwuyam@chu-grenoble.fr

Pr Jean-Louis PEPIN, Pôle de Rééducation et Physiologie, Laboratoire d'EFCR et Laboratoire du Sommeil, CHU Michallon, BP 217, 38043 Grenoble Cedex 9. Tél : 04.76.76.55.16 Fax : 04.76.76.55.86 E-mail : JPepin@chu-grenoble.fr

Dr Renaud TAMISIER, Pôle de Rééducation et Physiologie, Laboratoire d'EFCR et Laboratoire du Sommeil, CHU Michallon, BP 217, 38043 Grenoble Cedex 9. Tél : 04.76.76.55.16 Fax : 04.76.76.55.86 E-mail : RTamisier@chu-grenoble.fr

Pr Dominique PERENNOU, Pôle Rééducation & Physiologie, Pole Locomotion, Rééducation & Physiologie, Clinique Universitaire de Médecine Physique et Réadaptation. Institut de Rééducation, Hôpital Sud. Téléphone : 0476 76 60 84. E-mail DPerennou@chu-grenoble.fr.

<u>Responsable scientifique et associés à la recherche :</u>

Mr Samuel VERGES, Laboratoire HP2 (INSERM U1042) et UF Recherche sur l'Exercice, Université Joseph Fourier et CHU, Grenoble. Tel : 04-76-76-28-60 Fax : 04-76-76-56-17 E-mail : sverges@chu-grenoble.fr.

Pr. Guillaume MILLET, Laboratoire Physiologie de l'Exercice, Université Jean Monnet, Saint-Etienne, Tel : 04-77-12-03-66 Fax : 04-77-12-72-29 E-mail : guillaume.millet@univ-st-etienne.fr.

Mr Mathieu GRUET, Laboratoire HP2 (INSERM U1042) et UF Recherche sur l'Exercice, Université Joseph Fourier et CHU, Grenoble. Tel : 04-76-76-28-60 Fax : 04-76-76-56-17 Email : mgruet@chu-grenoble.fr.

Mr Thomas RUPP, Laboratoire HP2 (INSERM U1042) et UF Recherche sur l'Exercice, Université Joseph Fourier et CHU, Grenoble. Tel : 04-76-76-28-60 Fax : 04-76-76-56-17 Email : trupp@chu-grenoble.fr.

Pr. Stéphane PERREY, Laboratoire Efficience et Déficience motrice, Université Montpellier-1, Tel : 04-67-41-57-61 Fax : 04-67-41-57-08 E-mail : stephane.perrey@univ-montp1.fr

Mr Sébastien BAILLIEUL, Pôle Locomotion, Rééducation & Physiologie, UM Médecine du Sport & des Activités Physiques, Recherche sur l'exercice & Réhabilitation, CHU de Grenoble, Laboratoire HP2 (INSERM U1042) Université Joseph Fourier et CHU, Grenoble. Tel : 04-76-76-54-94. Email : sbaillieul@chu-grenoble.fr.

SOMMAIRE

1 - INTRODUCTION 8

1-1 FACTEURS LIMITANT LA PERFORMANCE : UN NOUVEAU PARADIGME 8
1-2 L'EXERCICE EN CONDITIONS HYPOXIQUES 8
1-3 LIMITATION A L'EFFORT DES PATIENTS HYPOXEMIQUES 9

2 - JUSTIFICATION DE L'ETUDE : DONNES DE LA LITTERATURE 10

2-1 PERTURBATIONS CEREBRALES ASSOCIEES A L'HYPOXIE : IMPLICATION POUR LA REPONSE A L'EFFORT 10
2-2 FLUX SANGUIN CEREBRAL, EXCITABILITE CORTICALE ET COMMANDE MOTRICE CHEZ LE PATIENT HYPOXEMIQUE 12

3 - OBJECTIFS DE L'ETUDE 13

4 - CRITERES D'EVALUATION 14

4-1 CRITERES DE JUGEMENT PRINCIPAL 14
4-2 CRITERES DE JUGEMENT SECONDAIRE 14
4-2 CRITERES DE SECURITE 14
4-2-1 Evénements indésirables (EvI) 15
4-2-2 Effet indésirable (EI) 15
4-2-3 Effet ou événement indésirable grave 15
4-2-4 Spécificités du protocole 15
4-2-5 Evénements non graves et liés soumis à déclaration immédiate 16
4-2-6 Evénements graves à ne pas déclarer immédiatement 16
4-2-7 Effet indésirable inattendu 16

5 - ELECTION DES PATIENTS 17

5-1 CALCUL DU NOMBRE DE SUJETS 17
5-2 SOURCE DE RECRUTEMENT DES PATIENTS 17
5-3 CRITERES D'INCLUSION 18
5-3-1 Patients BPCO 18
5-3-2 Patients SAOS 18
5-3-3 Sujets contrôles 18
5-4 CRITERES DE NON-INCLUSION 18
5-4-1 Patients BPCO et SAOS 18
5-4-2 Sujets contrôles 19

6 - PLAN EXPERIMENTAL 19

6-1 TYPE D'ETUDE 19
6-2 PROTOCOLE 19
6-3 VISITE DE SELECTION (J0 : J1 - 6 JOURS MINIMUM) 20
6-4 VISITE 1 - PATIENTS BPCO, SAOS ET SUJETS CONTROLES (J1 : EVALUATION DE LA MARCHE ET DE LA POSTURE, PUIS TACHE FATIGANTE N°1 : PEDALAGE SUR ERGOCYCLE) 21
6-5 VISITE 2 – PATIENTS BPCO, SAOS ET SUJETS CONTROLES (J1 + 1 SEMAINE MINIMUM : TACHE FATIGANTE N°2 : EXTENSION DE GENOU) 25
6-6 VISITE 3 PATIENTS BPCO, SAOS ET SUJETS CONTROLES (J2 + 2 JOURS MINIMUM : TEST DE REPONSE VENTILATOIRE ET CEREBRO-VASCULAIRE) 25

6-7 VISITE 4 PATIENTS BPCO (J2 + 1 SEMAINE MINIMUM : TACHE FATIGANTE N°1 : PEDALAGE SUR ERGOCYCLE AVEC INHALATION D'UN MELANGE HYPEROXIQUE) 26
6-8 VISITE 5 PATIENTS BPCO (J4 + 1 SEMAINE MINIMUM : TACHE FATIGANTE N°2 : EXTENSION DE GENOU AVEC INHALATION D'UN MELANGE HYPEROXIQUE) 26
6-9 VISITE 4 PATIENTS SAOS (POST TRAITEMENT PAR PPC D'UNE DUREE DE DEUX MOIS, J3 + 8 SEMAINES MINIMUM: EVALUATION DE LA LOCOMOTION ET DE LA POSTURE EN AIR AMBIANT ET TACHE FATIGANTE N°1 : PEDALAGE SUR ERGOCYCLE) 26
6-10 VISITE 5 PATIENTS SAOS (POST TRAITEMENT PAR PPC D'UNE DUREE DE DEUX MOIS, J4 + 1 SEMAINE MINIMUM : TACHE FATIGANTE N°2 : EXTENSION DE GENOU) 26
6-11 VISITE 6 PATIENTS SAOS (POST TRAITEMENT PAR PPC D'UNE DUREE DE DEUX MOIS, J5 + 2 JOURS MINIMUM : TEST DE REPONSE VENTILATOIRE ET CEREBRO-VASCULAIRE) 26
6-12 TRAITEMENT PAR PPC (PATIENTS SAOS) 27
6-13 PLANNING DES VISITES 27
6-13-1 Patients BPCO *28*
6-13-2 Patients SAOS *29*
6-13-3 Sujets contrôles *30*
6-14 ARRET PREMATURE DE L'ETUDE 31
6-15 CALENDRIER PREVISIONNEL 31

7 - GESTION DES DONNEES 31

8 – ANALYSE STATISTIQUE 32

9 - ASPECTS MATERIELS ET LEGAUX 33

9-1 BALANCE BENEFICE / RISQUE 33
9-2 CONSENTEMENT DU PATIENT 34
9-3 PROTECTION DES PERSONNES 34
9-4 COUTS POUR LE PATIENT 35
9-5 INFORMATIQUE ET LIBERTE 35
9-6 ACCES AUX RESULTATS 35
9-7 CONTRAT D'ASSURANCE 35
9-8 SECRET PROFESSIONNEL- CONFIDENTIALITE 36
9-9 PUBLICATIONS 36
9-10 ARCHIVAGE 36

11 - REFERENCES BILIOGRAPHIQUES 38

12 - ANNEXES 43

12-1 NOTES D'INFORMATION AU PATIENT 43
12-1-1 Patients BPCO *43*
12-1-2 Patients SAOS *48*
12-1-3 Sujets contrôles *55*
12-2 CONSENTEMENT PATIENT / TEMOIN 62
12-3 CV DES INVESTIGATEURS 65

Résumé du protocole

Le cerveau à l'effort : effets de l'hypoxie chez le malade respiratoire

Centre d'étude : C.H.U. de Grenoble

Investigateur coordonnateur: Pr. Patrick LEVY

Investigateurs associés :
Dr. Bernard WUYAM, Pr. Jean-Louis PEPIN, Dr. Renaud TAMISIER, Pr. Dominique PERENNOU.
Responsable scientifique et associés à la recherche :
Mr Samuel VERGES, Pr. Guillaume MILLET, Mr Mathieu GRUET, Mr Thomas RUPP, Pr. Stéphane PERREY, Mr Sébastien BAILLIEUL.

Promoteur : C.H.U. de Grenoble.

Personne autorisée à signer le protocole au nom du promoteur :

Mme Hélène SABBAH-GUILLAUME, Directrice de la Recherche et des Partenariats
Délégation à la Recherche Clinique et à l'Innovation

Objectifs

- Etudier les effets de l'hypoxie sur la fonction cérébrale chez des patients atteints de maladie chronique respiratoire (broncho-pneumopathie chronique obstructive : BPCO et syndrome d'apnée obstructive du sommeil : SAOS) en comparaison à des sujets sains.
- Evaluer les réponses neurophysiologiques du cerveau au repos et à l'exercice, incluant la perfusion et l'oxygénation cérébrale, l'excitabilité corticale et la commande motrice résultante, ainsi que les perturbations du contrôle non automatique de la marche et de la posture chez des sujets hypoxémiques (diurne pour les patients BPCO et nocturne pour les patients SAOS) avant et après un traitement visant à corriger les anomalies des gaz du sang (supplémentation en O_2 pour les patients BPCO et traitement par pression positive continue (PPC) pour les patients SAOS).

Méthodologie : Etude physiopathologique prospective, contrôlée et interventionnelle

Nombre total de sujets : 18 patients BPCO, 18 patients SAOS et 36 sujets sains contrôles

Critères d'inclusion :

Patients BPCO
- stades GOLD III – IV, indice de Tiffeneau VEMS/CVF < 0,7 et VEMS post bronchodilatateur < 50% des valeurs prédites
- BMI < 30 kg/m²
- Age entre 18 et 80 ans
- Non-fumeur ou ex-fumeur (arrêt depuis plus de 3 mois)
- État stable depuis plus de 3 mois
- $PaCO_2$ < 45 mmHg au repos en air ambiant
- Pas de diagnostic de SAOS.

Patients SAOS
- SAOS sévères récemment diagnostiqués (index apnée/hypopnée (AHI) >30)
- Score sur l'échelle de somnolence d'Epworth >10
- Age entre 18 et 80 ans
- BMI < 30 kg/m²

Sujets contrôles
- Age entre 18 et 80 ans
- BMI < 30 kg/m²
- Non-fumeur
- Sans pathologies chroniques respiratoires, cardiovasculaires, métaboliques, rénales ou neuromusculaires, vestibulaires et/ou de la vision.

Critères de non-inclusion :

Patients BPCO et SAOS
- Pathologies cardio-vasculaires, neuromusculaires, métaboliques, rénales
- Alcoolisme
- BMI > 30 kg/m²
- Pathologies psychiatriques ou ATCD de troubles du comportement, les troubles de la vision, la présence d'un syndrome vestibulaire, les pathologies neurologiques susceptibles de perturber le contrôle postural et la marche (Maladie de Parkinson et syndrome parkinsonien, accident vasculaire cérébral), les troubles cognitifs.
- Non-stabilité depuis moins de 3 mois.
- Contre-indication à l'application d'un champ magnétique.

Sujets contrôles
- Pathologies respiratoires, cardio-vasculaires, neuromusculaires, métaboliques, rénales
- Alcoolisme
- BMI > 30 kg/m²
- Pathologies psychiatriques ou ATCD de troubles du comportement
- Contre-indication à l'application d'un champ magnétique

Durée de participation pour un sujet : 8 semaines (patients BPCO) - 14 semaines (patients SAOS) - 4 semaines (sujets contrôles)

Durée totale de l'étude : 24 mois

Critères de jugement principal:
- Modification des quantités de désoxyhémoglobine et hémoglobine totale musculaires et cérébrales mesurées par spectroscopie proche infrarouge (NIRS) du repos à l'effort ou en réponse à l'inhalation de fractions variables d'O_2 et de CO_2,

Critères de jugement secondaire :
- Modification pré-post effort de l'amplitude du potentiel évoqué moteur ainsi que de la période de silence en réponse à une stimulation magnétique transcraniale (TMS),
- Modification des paramètres locomoteurs (cycle de marche, longueur du pas) et posturographiques (longueur maximale, surface et vitesse d'oscillation du centre de pression), en condition de simple et double tâche.
- Modification des paramètres d'oxygénation cérébrale et d'excitabilité corticale, de posture et de marche suite à une oxygénothérapie chez le patient BPCO et à un traitement par pression positive continue chez le patient SAOS.

**Critères de sécurité :** Recueil clinique des évènements indésirables
**Avis favorable du CPP Sud Est V à Grenoble**, le : 4/04/2012<br><br>**Autorisation de l'AFSSAPS**, le : 16/02/2012

1 - INTRODUCTION

1-1 FACTEURS LIMITANT LA PERFORMANCE : UN NOUVEAU PARADIGME

De nombreuses études physiologiques se sont intéressées aux facteurs limitant la performance à l'effort et en particulier aux causes de la fatigue. Certains travaux se sont notamment intéressés aux relations entre la performance en endurance et les facteurs circulatoires, métaboliques, musculaires ou nutritionnels(5, 8, 49). S**elon le paradigme classiquement accepté, les limites de l'endurance s'expliqueraient pour l'essentiel par des facteurs métaboliques à l'échelon musculaire.** Cependant, ce paradigme ne permet pas, dans certaines situations expérimentales d'identifier les facteurs limitant la performance physique, appelant ainsi à une approche alternative(33, 50). Certaines études ont apporté des arguments en faveur de l'hypothèse selon laquelle le système nerveux central (SNC) pouvait dans certaines situations présenter des difficultés à activer adéquatement les motoneurones, illustrant ainsi le concept de **fatigue centrale**(9, 35, 38, 70). Différentes théories impliquant une accumulation ou une déplétion de substances au niveau du cerveau ont été proposées pour expliquer cette fatigue(10, 19, 24). Seules quelques études récentes ont cependant évalué directement **les réponses cérébrales métaboliques ou neuro-humorales au cours d'un exercice fatigant**(18, 30, 51).

La chute de la force isométrique post exercice est l'une des caractéristiques de la fatigue musculaire squelettique pour laquelle l'implication de l'altération de la fonction contractile du fait de facteurs localisés dans le muscle est bien admise(79). De manière similaire, des troubles métaboliques, circulatoires, neuro-humoraux ou des perturbations de l'homéostasie cérébrale pourraient également conduire à une fatigue centrale. Le concept simplifié selon lequel «l'effort commence et se termine dans le cerveau» a ainsi été proposé(33). Noakes et al.(50) ont récemment suggéré l'existence d'une entité fonctionnelle, le «central governor», qui régulerait le recrutement des masses musculaires en termes d'intensité et de durée jusqu'à un seuil tolérable au-delà duquel des dommages pourraient survenir au sein des organes vitaux. Ce concept d'entité centrale demeure cependant très théorique, soulignant la nécessité d'une part de procéder à des mesures objectives capables de fournir une **description détaillée de la réponse cérébrale à l'exercice,** et d'autre part d'évaluer son **implication réelle dans des contextes environnementaux et pathologiques spécifiques.**

1-2 L'EXERCICE EN CONDITIONS HYPOXIQUES

Parmi les différentes situations ou le paradigme classique de la fatigue ne semble pas s'appliquer, **l'exercice en condition hypoxique** est certainement la plus remarquable. Les neurones centraux ont besoin d'un apport en O_2 régulier et suffisamment élevé afin d'assurer un fonctionnement cellulaire optimal. A effort maximal au cours d'un exercice intense réalisé en haute altitude, il n'y a que peu ou pas de signes de fatigue métabolique au niveau des muscles actifs(34) et le débit cardiaque n'atteint pas ses limites théoriques(78). Le SNC pourrait avoir un rôle important dans l'arrêt de l'effort en condition hypoxique avec comme objectif de préserver les différents organes vitaux. Certains travaux ont ainsi montré que **l'oxygénation cérébrale** à l'exercice pouvait être fortement modifiée en condition hypoxique comparativement à une situation normoxique(2, 62, 72). Il a été montré que **l'excitabilité corticale**, mesurée par stimulation magnétique transcranienne (TMS), pouvait être modifiée en situation d'hypoxie(22, 68, 69). Enfin, la prise en charge de cette hypoxie (par suplémentation en O_2) permet d'améliorer sensiblement la performance physique(48, 67). En

revanche, la relation entre les phénomènes d'altération de l'oxygénation cérébrale et de fatigue centrale demeure encore méconnue. Seuls quelques résultats récents obtenus chez le sujet sain au cours d'un exercice épuisant réalisé en condition hypoxique suggèrent une relation entre les deux phénomènes(57, 58). Ainsi, **les mécanismes impliqués dans la fatigue et en particulier leur composante cérébrale en condition hypoxique restent encore à éclaircir.**

1-3 LIMITATION A L'EFFORT DES PATIENTS HYPOXEMIQUES

La tolérance à l'effort est très fréquemment réduite chez des patients présentant une pathologie chronique respiratoire. De nombreuses études ont démontré le rôle crucial qu'occupe la **tolérance à un exercice physique dans l'évolution d'une maladie respiratoire chronique.** Par exemple, différents marqueurs de la tolérance à l'effort sont reliés avec le pronostic vital et l'espérance de vie chez des patients atteints de broncho-pneumopathie chronique obstructive (BPCO)(15). Chez des patients présentant un syndrome d'apnée obstructive du sommeil (SAOS), l'amélioration des aptitudes physiques permet d'obtenir des bénéfices non négligeables en terme de qualité de vie(4). Une meilleure compréhension du handicap à l'effort ainsi que l'amélioration de la tolérance à l'effort est aujourd'hui un objectif important pour la réhabilitation de ces patients(61). **L'évaluation et l'identification des facteurs potentiellement impliqués dans l'intolérance à l'effort** sont donc des étapes indispensables dans l'objectif d'optimiser les stratégies de prise en charge. Analyser les effets de l'hypoxie sur le cerveau à l'exercice est en particulier pertinent dans l'optique d'une compréhension exhaustive des mécanismes impliqués dans l'intolérance à l'effort des patients présentant une perturbation des gaz du sang. En effet, l'hypoxémie artérielle est une des conséquences communes à de nombreuses pathologies chroniques respiratoires. A la manière de l'altitude chez le sujet sain, elle est ainsi connue pour réduire la tolérance à l'effort mais les mécanismes sous-jacents demeurent encore discutés et nécessitent des investigations supplémentaires.

Si de nombreuses études se sont intéressées aux mécanismes régulateurs du débit sanguin cérébral (DSC) en haute altitude, peu de travaux ont analysé ses éventuelles perturbations associées aux maladies chroniques respiratoires(32). Seules quelques données existent concernant l'excitabilité corticale (telle qu'évaluée par TMS) chez des patients hypoxémiques chroniques(45, 53). Chez les patients SAOS par exemple présentant un pattern hypoxémique spécifique avec des séquences nocturnes de désoxygénation–réoxygénation, des perturbations de la circulation cérébrale(20, 59) et de la fonction neuromusculaire(17, 42) ont été rapportées. **En revanche, les conséquences chez ces patients de modifications de perfusion cérébrale, d'oxygénation et d'activité neuronale d'un point de vue fonctionnelle (e.g. intolérance à l'effort) restent à évaluer.** Des études complémentaires sont donc indispensables pour comprendre l'impact de la maladie sur la fonction cérébrale et son rôle dans le handicap à l'effort. Ainsi, les résultats de ce projet de recherche permettront de mieux comprendre les conséquences physiopathologiques des pathologies hypoxémiques et apporteront des informations importantes pour la prise en charge de ces malades.

2 - JUSTIFICATION DE L'ETUDE : DONNES DE LA LITTERATURE

2-1 PERTURBATIONS CEREBRALES ASSOCIEES A L'HYPOXIE : IMPLICATION POUR LA REPONSE A L'EFFORT

L'oxygénation cérébrale est déterminée par le produit de CaO_2 et du DSC, la modification de l'un ou l'autre pouvant affecter la disponibilité en O_2 neuronale. En plus de l'effet de la PaO_2, le DSC est également régulé par la $PaCO_2$(11). A l'exercice en hypoxie, la chute de la PaO_2 associée à une éventuelle diminution de la $PaCO_2$ et donc du DSC peut engendrer un déséquilibre important entre la demande et la fourniture en O_2 cérébral. Ceci a été confirmé par l'évaluation de l'oxygénation cérébrale chez le sujet sain par spectroscopie proche infrarouge (NIRS, permettant une mesure continue non invasive des quantités de désoxyhémoglobine et oxyhémoglobine tissulaire) à l'effort en hypoxie(22, 62, 66, 67). Une réduction de l'oxygénation et de la perfusion cérébrale lors de taches motrices non fatigantes en hypoxie a également été démontrée par techniques RMN(72). Ainsi **des perturbations significatives de la perfusion et de l'oxygénation cérébrales sont susceptibles de se produire à l'effort en hypoxie et pourraient constituer des mécanismes sous-jacents à l'altération de la commande motrice et de la performance à l'effort**. Cette hypothèse est d'autant plus probable que la fonction cérébrale apparait se détériorer lorsque son oxygénation est réduite de plus de 10%(21, 77), alors que les muscles squelettiques maintiennent leur fonctionnement malgré des désaturations en O_2 clairement supérieures à 10%(3).

Certaines études ont évaluée la fonction corticale en hypoxie chez le sujet sain en termes d'excitabilité et d'activation corticales. Après exposition hypoxique au repos pour une durée de quelques dizaines de minutes à plusieurs jours, il a été rapporté une altération de l'excitabilité corticale(44, 68). Récemment, Goodall et al.(22) ont montré que la réduction de performance lors d'un exercice d'extension du genou en hypoxie sévère était associée à une réduction de l'activation centrale telle qu'évaluée par TMS, c'est-à-dire à une moindre capacité à recruter de façon maximale les unités motrices du muscle sollicité. Ainsi, ces études utilisant la TMS comme technique d'évaluation directe de la fonction corticale suggèrent que **l'hypoxie puisse perturber de façon significative la réponse des neurones centraux ainsi que la production d'une commande motrice générée au niveau du cortex moteur**.

Une modification du « turnover » des neurotransmetteurs cérébraux serait également une conséquence de l'hypoxie sévère(16). Ainsi, des dysfonctions du SNC pourraient être à la fois le fruit de perturbations de l'homéostasie ionique dans les cellules nerveuses(26) mais également la conséquence de la libération extracellulaire de neurotransmetteurs affectant les membranes post-synaptiques(25). Une réduction de l'activité électro-encéphalographique (EEG) peut ainsi être observée chez des sujets réalisant une tache informatique et soumis à différents niveaux d'hypoxie (PaO_2 : 30 à 50 mmHg), comparativement à une situation normoxique(55). Enfin, une réduction similaire de l'activité EEG a également été retrouvée en altitude simulée (3000 m)(54), supportant **l'hypothèse d'une altération de la fonction électrique du cerveau en condition hypoxique.**

Certains protocoles manipulant la FiO_2 lors de tests d'effort chez le sujet sain fournissent des éléments supplémentaires de compréhension de l'impact de la fonction cérébrale sur la

performance à l'effort. Amann et al.(2) ont évalué des sujets sains lors d'un même exercice épuisant (pédalage à puissance constante) réalisé avec inhalation d'un gaz normoxique (air ambiant) ou d'un mélange hypoxique (FiO_2 = 0,10-0,15%). Juste avant l'arrêt de l'effort par épuisement, l'oxygénation artérielle était augmentée subrepticement en remplaçant le gaz inspiré par un mélange hyperoxique (FiO_2 = 0,30). Après réoxygénation dans la condition d'hypoxie sévère uniquement, l'oxygénation cérébrale augmentait et les sujets étaient immédiatement capables de continuer l'exercice pendant 3-4 minutes supplémentaires. Ce résultat est en accord avec d'autres études ayant analysé les effets de la réoxygénation dans des conditions d'hypoxie sévère, aiguë ou chronique(12, 34, 66, 67). L'ensemble de ces données suggère que **l'exercice en condition d'hypoxie sévère peut constituer une menace suffisante pour l'oxygénation du cerveau et la commande motrice, ceci ayant pour conséquence une diminution de la performance à l'effort.**

Les conséquences cliniques de l'hypoxie cérébrale notamment de l'hypoxie intermittente cyclique, restent cependant incomplètement caractérisées, et, parmi les éléments recherchés pouvant traduire une dysfonction du système nerveux central, la fatigabilité, et la somnolence diurne excessive ont un impact important sur les activités de la vie quotidienne, l'activité physique, ainsi que sur la qualité de vie des patients. Les complications neurologiques ont principalement été étudiées sous l'angle des accidents vasculaires cérébraux et de la fragmentation du sommeil(39). Par contre, l'étude des retentissements neuropsychologiques du SAOS est plus récente et concerne, pour majeure partie, les fonctions cognitives et en particulier les fonctions exécutives (7, 36, 39, 43, 47). Les fonctions exécutives sont définies comme étant la capacité à planifier et exécuter des comportements et tâches complexes orientés vers un but. Ce sont des fonctions indispensables au fonctionnement de l'individu dans la vie quotidienne. Elles impliquent les structures corticales préfrontales et sous corticales en interaction avec l'ensemble des aires associatives. Une atteinte des performances psychomotrices est également décrite (altération de la coordination motrice fine)(6). Bien que les résultats des études structurelles cérébrales utilisant des techniques d'imagerie et s'intéressant à la substance grise soient hétérogènes, l'atteinte de l'hippocampe y est rapportée de manière constante. La lésion cérébrale constatée est, outre le développement d'infarctus cérébraux, une réduction de volume de la substance grise(41). Les études se basant sur les techniques de morphométrie basée sur les Voxels (Voxel-Based Morphometry – VBM) retrouvent, outre une atteinte de l'hippocampe, une atteinte des régions frontale, pariétale, temporale et cérébelleuse (46, 52). Par ailleurs, Canessa et al.(13) ont rapporté une corrélation statistiquement significative entre la réduction quantitative localisée de substance grise et les performances sur le test de Stroop, corroborant l'hypothèse selon laquelle le syndrome dysexécutif a une place centrale dans l'atteinte neuropsychologique du SAOS. Une amélioration significative des performances cognitives (performances mnésiques et exécutives) corrélée à une augmentation de volume de la substance grise dans les régions de l'hippocampe, du cortex orbito-frontal médial et du gyrus frontal supérieur droit ont également été décrits dans cette étude après trois mois de traitement par appareillage en pression positive continue. Ces résultats restent cependant isolés, contradictoires avec les résultats antérieurs et mériteraient d'être étayés.

Marche et contrôle postural :

La marche et le maintien d'une posture sont des activités qui revêtent un caractère implicite : ce sont des processus automatisés, reposant sur des structures sous-corticales et médullaires.
Cet aspect automatique ne doit néanmoins pas occulter le caractère complexe de la marche, qui implique d'autres impératifs : propulsion du corps dans l'espace, équilibre stable

(coordination posture et mouvement) et permettre une adaptation aux contraintes de l'environnement extérieur Ainsi, équilibre, posture et marche sont à considérer comme fonctions exécutives élaborées, impliquant l'intégration de l'attention, de la planification, de la mémoire ainsi que des processus moteurs, cognitifs et sensoriels(80, 81). Une perturbation des paramètres spatio-temporels à la marche (notamment en situation de double tâche) ainsi qu'une majoration du risque de chute ont également été décrites dans des populations de sujets présentant une altération des fonctions cognitives(27). La double tâche est définie comme étant la réalisation simultanée de deux tâches, l'une dite « primaire » et l'autre « secondaire. En condition de double tâche, les perturbations des paramètres de marche constatées seraient liées à un défaut d'allocation des ressources attentionnelles entre la marche (alors tâche secondaire) et la tâche cognitive (alors tâche primaire) ces deux tâches mettant en jeu les mêmes structures corticales(80).

A notre connaissance, il n'a pas été à ce jour décrit de troubles moteurs dans cette pathologie, en particulier de troubles de la marche ou du contrôle postural. Le SAOS, de par ses atteintes cérébrales structurelles (cortex préfrontal) et fonctionnelles notamment neuropsychologiques (fonctions exécutives)(39) serait responsable d'une perturbation du contrôle postural et du schéma de marche. Ces perturbations s'accompagneraient d'un retentissement sur l'autonomie des sujets, leur performance physique, leur qualité de vie ainsi qu'un risque augmenté de chute. Une partie de l'objectif est donc : (i) de mettre en évidence et caractériser d'éventuels troubles de la marche et du contrôle postural chez des sujets présentant un SAOS avant instauration du traitement par PPC, (ii) d'évaluer l'impact du traitement par PPC sur le contrôle postural et le schéma de marche.

2-2 FLUX SANGUIN CEREBRAL, EXCITABILITE CORTICALE ET COMMANDE MOTRICE CHEZ LE PATIENT HYPOXEMIQUE

L'hypoxémie artérielle présente dans plusieurs pathologies chroniques respiratoires peut engendrer des nombreuses dysfonctions et participer à l'intolérance à l'effort. Alors que différents travaux ont analysé les mécanismes régulateurs du DSC et de l'oxygénation du cerveau chez des sujets sains en altitude, très peu d'études se sont intéressées aux **modifications centrales pouvant survenir en conséquence de l'hypoxémie chez des sujets présentant une pathologie chronique respiratoire.**

Chez le patient BPCO, la majorité des études indiquent un DSC inchangé au repos comparativement à des sujets contrôles(14, 73, 74). Ces résultats sont étonnants puisque l'hypoxémie marquée et l'absence d'hypocapnie sont des facteurs connus pour augmenter le DSC en situation d'hypoxie(1). Il semble donc probable que l'effet vasodilatateur liée à l'hypoxémie couplée à la relative hypercapnie soient contrecarrés par des facteurs visant à réduire le DSC. Dans la seule étude qui a évalué la perfusion et l'oxygénation cérébrale à l'exercice chez des patients hypoxémiques présentant une atteinte pulmonaire sévère(32), les auteurs ont constaté une réduction de l'oxygénation cérébrale et ce malgré une augmentation de la perfusion cérébrale, suggérant un **trouble spécifique de l'oxygénation du cerveau chez ces patients à l'effort, dont les causes doivent encore êtres élucidées.** Les données relatives aux effets de l'hypoxie sur l'excitabilité neuronale et sur la fonction du cortex moteur demeurent également très limitées chez ces patients. Dans l'une des rares études conduites chez des patients hypoxémiques chroniques (BPCO, PaO_2<65mmHg)(53), l'excitabilité corticale a été évaluée par TMS. L'étude a révélé que l'inhibition intra-corticale et la période de silence corticale de ces patients étaient significativement réduites comparativement à des sujets sains contrôles alors que l'amplitude des potentiels moteurs évoqués (MEP) était similaire dans les deux groupes. **Le fait que ces adaptations corticales soient partiellement**

réversibles chez ces patients BPCO après trois mois d'oxygénothérapie confirme qu'elles sont induites par l'hypoxie. Mohamaed-Hussain et al. (45) ont montré que l'exacerbation aiguë du patient BPCO était associée à une accentuation de l'altération des voies cortico-spinales et de l'excitabilité corticale. A partir de ces données et de celles suggérant une altération de la commande motrice volontaire chez le patient BPCO(29, 64, 76), il peut être suggéré que l'altération de la commande motrice chez ces patients puisse être une conséquence de l'altération de la conduction corticospinale et/ou de l'excitabilité des voies corticales inhibitrices et excitatrices. Malgré ces quelques études, les conséquences de l'hypoxie chronique sur le fonctionnement du cerveau demeurent largement sous documentées, notamment au cours de l'exercice. Des études complémentaires sont donc nécessaires pour apprécier les **réponses cérébrales du patient hypoxémique au cours d'un effort musculaire.**

Le SAOS est une pathologie répandue caractérisée par des arrêts répétés des flux respiratoires à l'origine d'une perturbation du sommeil et d'épisodes hypoxiques. Il a été montré chez ces patients une altération des réponses vasodilatatrices à l'hypoxie aiguë au niveau des muscles de l'avant bras(60) mais également au niveau de la circulation cérébrale(20). Ainsi, dans l'étude de Reichmuth et al.(59), les auteurs ont montré que les réponses vasodilatatrices cérébrales à l'hypoxie étaient réduites chez la majorité de ces patients comparativement à des sujets sains contrôles. Certaines études ont également rapporté une atténuation des réponses vasodilatatrices cérébrales à l'hypercapnie chez les patients SAOS(40). Il semblerait que l'importance de ces troubles de la régulation vasculaire soit reliée à la sévérité du SAOS. Il a enfin été montré que ces différents troubles pouvaient êtres significativement réduits en traitant le SAOS par ventilation en pression positive continue (CPAP)(59). Ainsi, il est clair que les patients SAOS présentent une altération de la régulation du DSC qui pourrait être partiellement améliorée après un traitement CPAP. En revanche, **la possibilité que ces altérations vasculaires puissent perturber la délivrance en O_2 et l'utilisation des substrats énergétiques au niveau du cerveau lors de situations physiologiques comme l'exercice physique nécessite d'être évaluée spécifiquement.** De la même manière, **les conséquences d'un traitement CPAP sur l'oxygénation et la perfusion cérébrale à l'exercice devront être étudiées.** Le pattern spécifique de désoxygénation-réoxygénation des patients SAOS peut également induire une production de radicaux libres et un stress oxydatif à l'origine **d'altérations neuromusculaires**(31, 42). Chien et al.(17) ont ainsi montré récemment que les patients SAOS présentaient une performance musculaire réduite, une altération de propagation du potentiel d'action et une fatigabilité accrue des muscles inspiratoires. Alors que cette étude publiée en 2010 est la seule à s'être intéressée à la fonction musculaire périphérique du patient SAOS, aucune autre à ce jour n'a évalué les mécanismes d'activation volontaire ou de fatigue centrale dans cette population. Etant donné que nombre de ces patients présentent une tolérance à l'effort réduite, il peut être suggéré qu'une **altération de la fonction cérébrale et de la commande motrice** puisse expliquer au moins en partie l'handicap fonctionnel à l'activité physique des patients SAOS.

3 - OBJECTIFS DE L'ETUDE

Ce projet vise à étudier les effets de l'hypoxie sur la fonction cérébrale chez des patients atteints de maladie chronique respiratoire. Nous proposons d'évaluer les réponses neurophysiologiques du cerveau au repos et à l'exercice, incluant la perfusion et l'oxygénation cérébrale, l'excitabilité corticale et la commande motrice résultante chez des sujets hypoxémiques (diurne pour les patients BPCO et nocturne pour les patients SAOS)

avant et après un traitement visant à corriger les anomalies des gaz du sang (supplémentation en O_2 pour les patients BPCO et CPAP pour les patients SAOS).

Afin de réaliser ces différents objectifs, une **approche méthodologique multidisciplinaire et complémentaire sera adoptée** : la NIRS pour apprécier la perfusion et l'oxygénation cérébrale, la TMS, les techniques de neurostimulation (NES) et l'électromyographie (EMG) pour apprécier l'excitabilité corticale, mesurer le niveau d'activation centrale et la commande motrice.

D'une manière plus spécifique, les objectifs de ce projet seront les suivants :

- Mesurer la perfusion et l'oxygénation cérébrale, l'excitabilité corticale, les mécanismes d'activation volontaire et de fatigue centrale à l'effort chez le **patient hypoxémique chronique (BPCO et SAOS)** en comparaison à des sujets sains contrôles;
- Analyser les perturbations des paramètres locomoteurs (paramètres temporels du cycle de marche, longueur du pas) et posturographiques (longueur maximale, surface et vitesse d'oscillation du centre de pression), en condition de simple et double tâche, impliquant des niveaux de tâche mentale associées variée.
- Analyser les **effets aigus d'une amélioration de l'oxygénation artérielle** (inhalation d'un mélange hyperoxique) **chez des patients BPCO** sur la perfusion et l'oxygénation cérébrale, l'excitabilité corticale, les mécanismes d'activation volontaire et de fatigue centrale ;
- Evaluer les effets d'un traitement par **CPAP chez des patients SAOS** sur ces mêmes paramètres.

4 - CRITERES D'EVALUATION

4-1 CRITERES DE JUGEMENT PRINCIPAL

- Modification des quantités de désoxyhémoglobine et hémoglobine totale musculaires et cérébrales mesurées par spectroscopie proche infrarouge (NIRS) du repos à l'effort ou en réponse à l'inhalation de fractions variables d'O_2 et de CO_2.

4-2 CRITERES DE JUGEMENT SECONDAIRE

- Modification pré-post effort de l'amplitude du potentiel évoqué moteur ainsi que de la période de silence en réponse à une stimulation magnétique transcraniale (TMS),
- Modification des paramètres locomoteurs (paramètres temporels du cycle de marche, longueur du pas) et posturographiques (longueur maximale, surface et vitesse d'oscillation du centre de pression), en condition de simple et double tâche.
- Modification des paramètres d'oxygénation cérébrale et d'excitabilité corticale suite à une oxygénothérapie chez le patient BPCO et à un traitement par pression positive continue chez le patient SAOS.

4-2 CRITERES DE SECURITE

La vigilance et les mesures urgentes de sécurité seront en conformité avec le décret n°2006-477 du 26/04/2006 section IV, ainsi qu'avec les arrêtés et les décisions s'y rapportant.

Tout évènement survenant au cours de cette étude (maladie, accident ou prise de médicament) qu'il paraisse ou non en relation avec cette étude sera signalé dans les plus brefs délais au médecin en charge de cette recherche (Pr Patrick Levy, CHU de Grenoble), qui prendra les mesures nécessaires et en informera le Promoteur.

Dans le cadre d'un suivi, un entretien par téléphone pourra être effectué concernant les symptômes ressentis dans les heures et les jours suivant les tests (courbatures, céphalées bénignes persistantes au-delà de la période de surveillance) et leur résolution. Le Pr Levy ainsi que les médecins investigateurs se chargeront du suivi de ces éventuels symptômes ressentis par les sujets jusqu'à leur entière résolution. En cas de symptômes musculaires et/ou de céphalées persistantes, toute mesure appropriée sera prise.

Seront recensés les :

4-2-1 Evénements indésirables (EvI)

Toute manifestation nocive survenant chez une personne qui se prête à une recherche biomédicale que cette manifestation soit liée ou non à la recherche ou au(x) médicament(s) expérimental (aux), **aux dispositifs médicaux, aux produits sanguins labiles, produits du corps humains utilisés à des fins thérapeutiques, produits de thérapie cellulaire, produits cosmétiques ou de tatouage sur** le(s)quel(s) porte cette recherche.

4-2-2 Effet indésirable (EI)

Toute réaction nocive et non désirée à un médicament expérimental quelle que soit la dose administrée. Pour l'effet indésirable d'un dispositif médical ou d'un dispositif médical de diagnostic in vitro, toute réaction nocive et non désirée à un dispositif médical ou tout incident qui aurait pu entraîner cette réaction si une action appropriée n'avait pas été effectuée, chez une personne qui se prête à la recherche ou chez l'utilisateur du dispositif médical ou tout effet lié à une défaillance ou une altération d'un dispositif médical de diagnostic in vitro et néfaste pour la santé d'une personne qui se prête à la recherche.

4-2-3 Effet ou événement indésirable grave

L'effet ou événement grave est un effet ou événement indésirable ayant entraîné :

- Le décès
- La mise en jeu du pronostic vital
- Une invalidité ou une incapacité importante ou durable
- Une hospitalisation ou prolongation d'hospitalisation
- Une anomalie ou malformation congénitale
- Autres: Tout effet indésirable jugé comme grave par le professionnel de santé, en particulier les événements nécessitant une intervention pour éviter l'une des conséquences notées ci-dessus, et certains résultats d'examens paracliniques.

4-2-4 Spécificités du protocole

Les effets secondaires prévisibles sont :

- des courbatures au niveau du quadriceps du fait de la tâche motrice ou du pédalage sur cycloergomètre,
- un inconfort lors de l'application des stimulations électrique; cette application est peu douloureuse, la survenue d'une contraction musculaire involontaire (lors de la stimulation) créant un léger désagrément ;
- des céphalées suite à l'application de la stimulation magnétique corticale ; l'application de stimulation unique sous-maximale (et non pas 'stimulation répétitive à haute fréquence' comme dans le cadre des traitements de certaines pathologies neurologiques par exemple) rend minimal le risque de ce type d'effet secondaire.
- Des courbatures au niveau des muscles du membre inférieur du fait des tests de marche et de maintien d'une posture,
- Une sensation de fatigue à l'issue des tests de marche et posture, légère sur le plan physique liée à la marche ou la posture, mais également intellectuelle, liée à l'évaluation neuropsychologique ainsi qu'à la condition de double tâche (marcher et analyser, se maintenir et analyser).
- Des risques de déséquilibre pouvant aller jusqu'à la chute lors des tests de posture ou lors de l'évaluation à la marche. Elles seront prévenues par une sécurité passive sur le tapis (harnais) et des barrières de sécurité.

Ces techniques ont été utilisées lors de récents protocoles de recherche clinique conduits par notre équipe, y compris chez le patient (protocole EEMUR, protocole CERVOX, protocole VALLOT 2011), sans effet secondaire. Pour la posturographie et les tests de marche, les tests seront réalisés à l'institut de Rééducation (Pr Dominic PERENNOU) et sont conformes aux procédures de sécurité utilisées pour les patients à risque de chute encore plus importants comme les patients après AVC constitués.

4-2-5 Evénements non graves et liés soumis à déclaration immédiate

Tous les événements indésirables non graves et/ou les résultats d'analyse anormaux, définis dans le protocole comme déterminants pour l'évaluation de la sécurité des personnes qui se prêtent à l'essai clinique, doivent être notifiés au promoteur par l'investigateur, conformément aux modalités et délais précisés dans le protocole.

4-2-6 Evénements graves à ne pas déclarer immédiatement

Sont recensés ici le plus exhaustivement possible les événements indésirables graves connus et attendus (événements liés aux interventions de l'étude) des différents produits expérimentaux et des examens pratiqués pendant l'étude.

4-2-7 Effet indésirable inattendu

L'effet inattendu est un effet indésirable dont la nature, la gravité ou l'évolution ne correspondent pas aux informations contenues dans le résumé des caractéristiques du produit, la brochure investigateur ou autre référentiel reconnu par les autorités.

La déclaration des événements indésirables sera effectuée conformément à la procédure interne du CHU de Grenoble CRPV-PRO-002.

Les événements indésirables graves seront immédiatement notifiés, respectivement par l'investigateur au promoteur et par le promoteur à l'autorité compétente AFSSAPS.
En cas de survenue d'un événement indésirable, l'investigateur avertira le centre dans les délais :

Edith Schir
Centre Régional de Pharmacovigilance
eschir@chu-grenoble.fr
Pharmacovigilance@chu-grenoble.fr
Fax : 04 76 76 51 45 ; Tel : 04 76 76 56 55

En cas d'événements ou d'effets indésirables graves, le patient bénéficiera d'un suivi médical par l'investigateur jusqu'à guérison des symptômes. L'investigateur informera dans les meilleurs délais la personne responsable de la vigilance des essais cliniques pour la tenir informée de l'évolution de l'événement indésirable grave ou de l'effet indésirable grave.

5 - ELECTION DES PATIENTS

5-1 CALCUL DU NOMBRE DE SUJETS

Dix-huit patients BPCO, 18 patients SAOS et 36 sujets sains contrôles (appariés pour l'âge, le sexe et le BMI) seront recrutés. L'effectif de ces groupes a été calculé sur la base des effets cérébraux de l'exposition hypoxique chez le sujet sain mesurés au sein de notre laboratoire lors d'un projet en cours de publication (Protocole CERVOX, projet Blanc ANR 2009) et sur des études ayant utilisées les mêmes méthodologies (excitabilité corticale évaluée par TMS(23, 68), oxygénation et perfusion cérébrale par NIRS à l'effort(67)).

5-2 SOURCE DE RECRUTEMENT DES PATIENTS

Les patients seront recrutés au Centre Hospitalier Universitaire de Grenoble lors des consultations au Laboratoire d'Exploration Fonctionnelle Cardio-Respiratoire et au Laboratoire du Sommeil. Les sujets contrôles (appariés aux patients pour l'âge, le sexe et le BMI) seront recrutés par voie d'affichage au sein du CHU et éventuellement par annonce dans la presse régionale. Le recrutement sera basé sur un examen clinique permettant de vérifier les différents critères d'inclusion et de non-inclusion.

La participation simultanée à une recherche clinique ou thérapeutique durant l'étude n'est pas interdite dans la mesure où elle n'interfère pas avec les réponses neuromusculaires et à l'effort mesurées dans le cadre du présent protocole. Après la fin de cette expérimentation, il n'existe pas de période d'exclusion pour la participation à une éventuelle autre recherche.

5-3 CRITERES D'INCLUSION

5-3-1 Patients BPCO

- Stades GOLD III – IV, indice de Tiffeneau VEMS/CVF < 0,7 et VEMS post bronchodilatateur < 50% des valeurs prédites
- BMI < 30 kg/m²
- Age entre 18 et 80 ans
- Non-fumeur ou ex-fumeur (arrêt depuis plus de 3 mois)
- État stable depuis plus de 3 mois
- $PaCO_2$ < 45 mmHg au repos en air ambiant
- Pas de diagnostic de SAOS
- Patients bénéficiant d'une couverture de la sécurité sociale

5-3-2 Patients SAOS

- SAOS sévères récemment diagnostiqués (index apnée/hypopnée (AHI) >30)
- Score sur l'échelle de somnolence d'Epworth >10
- Age entre 18 et 80 ans
- BMI < 30 kg/m²
- Patients bénéficiant d'une couverture de la sécurité sociale

5-3-3 Sujets contrôles

- Age entre 18 et 80 ans
- BMI < 30 kg/m²
- Non fumeur
- Sans pathologie chronique respiratoire, cardiovasculaire, métabolique, rénales ou neuromusculaire, vestibulaires et/ou de la vision.

5-4 CRITERES DE NON-INCLUSION

5-4-1 Patients BPCO et SAOS

- Pathologies cardio-vasculaires, neuromusculaires, métaboliques, rénales
- Alcoolisme
- BMI > 30 kg/m²
- Pathologies psychiatriques ou ATCD de troubles du comportement, les troubles de la vision, la présence d'un syndrome vestibulaire, les pathologies neurologiques susceptibles de perturber le contrôle postural et la marche (Maladie de Parkinson et syndrome parkinsonien, accident vasculaire cérébral), les troubles cognitifs.

- Non-stabilité depuis moins de 3 mois
- Patients sous tutelle ou curatelle
- Femmes qui allaitent
- Patients non affiliés à la sécurité sociale
- Personne privée de liberté, majeur protégé par la loi, personne hospitalisée
- Personne sous traitement vasoactif

5-4-2 Sujets contrôles

- Pathologies respiratoires, cardio-vasculaires, neuromusculaires, métaboliques, rénales
- Alcoolisme
- BMI > 30 kg/m²
- Pathologies psychiatriques ou ATCD de troubles du comportement
- Contre-indication à l'application d'un champ magnétique
- Sujets sous tutelle ou curatelle
- Femmes qui allaitent
- Sujets non affiliés à la sécurité sociale
- Personne privée de liberté, majeur protégé par la loi, personne hospitalisée
- Personne sous traitement vasoactif

6 - PLAN EXPERIMENTAL

6-1 TYPE D'ETUDE

Etude physiopathologique prospective, contrôlée et interventionnelle

6-2 PROTOCOLE

La fonction cérébrale ainsi que la fatigue centrale des **patients BPCO, SAOS et des sujets contrôles** sont évaluées avant, pendant et après deux types de tâches motrices fatigantes standardisées :

- La première consiste en un exercice de pédalage sur cycloergomètre à 80% de la puissance maximale aérobie, conduit jusqu'à épuisement. La puissance maximale aérobie est déterminée au préalable par un test d'effort incrémental réalisé lors de la visite de sélection.
Pour l'évaluation du contrôle de la marche et de la posture, l'exercice est de faible intensité, représentant une marche sur tapis roulant à la vitesse de marche de confort du patient, en situation de simple mais aussi parfois de double tâche (tache mentale associée) et

accélérations/décélérations de +1km/h par rapport à la marche de confort. L'évaluation de la posture est réalisée sur plateforme de posture, avec situation d'instabilité à corriger, en simple et double tâche.

- La deuxième consiste en des contractions isolées du quadriceps (extension isométrique du genou, 5 secondes de contraction / 5 secondes de repos à 50% de la force maximale volontaire) conduites jusqu'à épuisement.

Chez le patient BPCO, les réponses cérébrales et la fatigue centrale associée aux tâches motrices sont appréciées dans deux conditions, lorsque l'effort est réalisé en inhalant un mélange normoxique (air ambiant) ou un mélange hyperoxique (FiO_2 = 0,4).
Chez les patients SAOS, les deux tâches motrices sont réalisées en air ambiant à deux reprises de façon identique, de même que l'évaluation de la marche et de la posture, avant et après 2 mois de traitement par PPC.

6-3 VISITE DE SELECTION (J0 : J1 - 6 jours minimum)

Les patients correspondant aux critères d'inclusion et ne répondant à aucun des critères de non-inclusion se verront proposer la participation au protocole. Ils reçoivent les informations concernant le protocole et signent le consentement s'ils le désirent.

Un examen clinique est réalisé au cours de cette visite, comportant un interrogatoire sur tout critère possible de non-inclusion, l'auscultation cardio-pulmonaire, ainsi qu'une mesure de la pression artérielle après 10 minutes repos.

Un enregistrement polysomnographique est également réalisé pour les patients SAOS. Il s'agit de l'examen de référence utilisé pour le diagnostic des arrêts respiratoires nocturnes tel que réalisé au sein du laboratoire du Sommeil du CHU de Grenoble. Il s'inscrit dans le bilan normal de tout patient suspect d'avoir cette maladie. Il associe le recueil de l'électroencéphalogramme, de l'électromyogramme, et de l'électro-oculogramme pour différencier les différents stades de sommeil (uniquement pour la polysomnographie). La quantification du débit aérien est faite par thermistances et par mesure de la pression nasale. L'effort respiratoire est apprécié par une sangle thoracique et abdominale associée à une mesure du temps de transit du pouls. Les conséquences des apnées et hypopnées sont appréciées en mesurant la saturation en oxygène (SaO_2) et l'électrocardiogramme. Cet examen est indolore (les capteurs sont collés ou fixés par du sparadrap sur la peau pendant la durée de l'enregistrement).

Il est pratiqué une exploration fonctionnelle respiratoire, un électrocardiogramme de repos et un test d'effort incrémental (selon le protocole habituel au sein du laboratoire EFCR : 2 min à 10-20W, puis +10-20W/min) avec mesure de la perception de l'effort (échelle de Borg), mesure des échanges gazeux, surveillance continue de l'ECG 12 pistes, établissement d'un profil tensionnel d'effort, mesure continue de la SaO_2 et mesure directe de la PaO_2, $PaCO_2$ par prélèvement capillaire artérialisé en fin d'échauffement et fin d'effort.

L'exploration fonctionnelle respiratoire sera réalisée lors de l'ensemble des visites, notamment pour s'assurer de la stabilité des paramètres pulmonaires.

Les **critères d'arrêt de l'effort** correspondent à l'impossibilité du sujet de poursuivre la tâche ou à la survenue d'un des critères d'arrêt des tests à l'effort : pâleur ou dyspnée brutale même inexpliquée, inconfort ou sensation d'angoisse même inexpliqués, modification tensionnelle (correspondant à l'absence d'élévation de la pression artérielle au cours de l'effort sur deux paliers consécutifs) à fortiori la baisse de la pression artérielle de plus de 20 mm Hg sur deux paliers consécutifs, l'existence de modifications de l'ECG d'effort (modification du segment ST, apparition de troubles du rythme ventriculaires surtout si les ESV ont tendance à s'aggraver, devenir plus nombreuses, polymorphes, avec des doublets, triplets, avec un R/T précoce, tous critères reconnus comme critères habituels d'un arrêt de l'effort dans un test d'effort incrémental.

Ce test d'effort servira également à déterminer les **80% de la puissance maximale aérobie** pour le protocole à charge constante sur ergocycle.

De plus, en cas d'accord de participation, les différentes évaluations sont présentées aux sujets et des procédures de familiarisation sont effectuées (stimulation magnétique, tâches motrices, etc.).

Enfin, pour les sujets porteurs d'OSAS, il sera, ensuite, réalisé une évaluation neuro-psychologique :
L'évaluation neuropsychologique des sujets permet une estimation de leurs performances cognitives, la passation des tests est effectuée par une neuropsychologue DE. Une information orale sur le déroulement des tests est délivrée au préalable aux sujets. La batterie de tests proposée qui leur est proposée permet une étude des fonctions les plus fréquemment altérées dans ce type de population. Les résultats de ces tests étant dépendant des conditions environnementales de leur passation, les sujets seront tous évalués à la même heure de la journée, à T0 et T1. La batterie de tests utilisée dans notre étude est composée :

Pour l'évaluation de la **mémoire épisodique** : Epreuve de rappel (indicé, libre)

Pour l'évaluation des **fonctions exécutives** : Test de STROOP (attention)
Trail Making Test (flexibilité mentale et vitesse de traitement de l'information (VTI))
Tour de Hanoï (capacités de planification)
Empan de chiffres (mémoire de travail)
Paced Auditory Serial Addition Test (attention soutenue)
Codes de la Wechsler Adult Intelligence Scale (VTI). La durée de l'évaluation neuropsychologique est d'une heure.

6-4 VISITE 1 - Patients BPCO, SAOS et sujets contrôles (J1 : évaluation de la marche et de la posture, puis Tâche fatigante n°1 : pédalage sur ergocycle)

(i) Evaluation posturographique :

L'évaluation des performances posturales est réalisée sur une plateforme TECHNOCONCEPT®, modèle Feetest 6©. Cet outil de mesure est composé de deux sabots de stabilométrie indépendants permettant un recueil des paramètres posturographiques tels que la longueur de déplacement du centre de pression, la surface et la vitesse d'oscillation du centre de pression.

Les sujets sont initialement familiarisés avec l'outil de mesure au cours de 2 acquisitions pré-test à T0 et T1. Chaque session d'évaluation est composée de la série d'acquisitions suivante, la durée de chaque acquisition définie *a priori* étant de trente secondes :

Première condition : les sujets sont positionnés debout, les bras le long du corps, un pied sur chaque sabot de mesure, tous deux alignés dans le plan frontal et écartés de 270 millimètres (distance inter-malléollaire interne), selon un angle de 15° de rotation externe par rapport au plan sagittal. Pour cette première condition de mesure, les sabots sont disposés sur un plan stable. Il est alors demandé aux sujets de maintenir cette posture en évitant dans la limite des leurs capacités les mouvements parasites, interférant avec les mesures. Des rampes d'appui sont placées de part et d'autre de la plateforme d'évaluation permettant aux sujets de se retenir en cas de déséquilibre.

Seconde condition : les sujets sont positionnés debout, les bras le long du corps, un pied sur chaque sabot de mesure, tous deux alignés dans le plan frontal et écartés de 270 millimètres (distance inter-malléollaire interne), selon un angle de 15° de rotation externe par rapport au plan sagittal. Pour cette deuxième condition de mesure, les sabots sont disposés sur une plateforme instable dite de BESSOU. La plateforme est disposée pour permettre des oscillations dans le plan frontal, selon un rayon de 550 millimètres. Il est alors demandé aux sujets de stabiliser la plateforme parallèlement au plan du sol, en évitant dans la limite de leurs capacités les mouvements parasites interférant avec les mesures. Des rampes d'appui sont placées de part et d'autre de la plateforme d'évaluation permettant aux sujets de se retenir en cas de déséquilibre.

Les mesures sont effectuées pour chaque condition, en situation de simple (quatre acquisitions) et de double tâche (quatre acquisitions). En condition de double tâche, la tâche secondaire utilisée est le test de STROOP, tâche de type attentionnelle, qui est ici utilisée dans une version informatisée. L'écran utilisé pour afficher le test sera placé à hauteur de regard. La réponse au test sera verbale, un recueil du nombre d'erreur sera effectué par l'évaluateur. L'ordre de passation des évaluations (première ou seconde condition, et pour chacune d'entre elle, en situation de simple ou de double tâche) est randomisé pour chaque sujet pour prévenir l'apparition de phénomènes de fatigue (coût attentionnel) et d'apprentissage inhérents à la situation de double tâche. Chaque session d'évaluation comportera au total 16 mesures pour une durée de 8 minutes.

(ii) Evaluation locomotrice :

L'évaluation des performances à la marche est réalisée sur tapis de marche BIODEX™, modèle Gait Trainer 3™. Les sujets sont placés dans un harnais de marche, assurant leur sécurité, et autorisant un schéma de marche naturel en préservant le ballant du bras. Des barrières de sécurité sont disposées de part et d'autre du tapis de marche, un bouton pressoir permettant un arrêt d'urgence en cas de survenue d'un événement indésirable est placé sur l'une d'elle. Ce dernier est couplé à l'OPTOGAIT™, système de détection optique composé d'une barre émettrice et d'une autre réceptrice, chacune étant dotée de 96 LEDS. Chaque barre est disposée de part et d'autre du tapis de marche, permettant ainsi un recueil des paramètres de marche (stride ou longueur du pas, durée de la phase de double appui, de la phase oscillante, du cycle de marche…).

La vitesse de marche naturelle des sujets est définie *a priori* au cours d'un test de marche de six minutes et servira de référence pour l'évaluation des paramètres locomoteurs à T0 et à T1. Les sujets sont familiarisés avec l'outil de mesure au cours de 2 acquisitions pré-test à T0 et T1.

Chaque session d'évaluation est composée de la série d'acquisitions suivante, la durée d'acquisition, définie *a priori* étant de 30 secondes :

Première condition : Les sujets marchent à vitesse naturelle (VN) (vitesse définie au préalable par la réalisation d'un test de marche de 6 minutes). Il est demandé aux sujets de marcher le plus naturellement possible, en regardant droit devant eux.

Seconde condition : Les sujets marchent à VN. Une perturbation de la marche, définie pour notre protocole comme étant une majoration de 1km/h de la VN survient de manière aléatoire à 7,5, 15 ou 22,5 secondes. La survenue de cette perturbation permettra de mettre en évidence pour chaque sujet d'une part les stratégies et d'autre part d'éventuelles difficultés d'adaptation des paramètres de marche et de son schéma chez nos sujets, tout en se rapprochant de conditions écologiques. Il est demandé aux sujets de marcher le plus naturellement possible, en regardant droit devant eux.

Les mesures sont effectuées pour chaque condition, en situation de simple (quatre acquisitions) et de double tâche (quatre acquisitions). En condition de double tâche, la tâche secondaire utilisée est le test de STROOP, tâche de type attentionnelle, qui est ici utilisée dans une version informatisée. L'écran utilisé pour afficher le test sera placé à hauteur de regard. La réponse au test sera verbale, un recueil du nombre d'erreur sera effectué par l'évaluateur. L'ordre de passation des évaluations (première ou seconde condition, et pour chacune d'entre elle, en situation de simple ou de double tâche) est randomisé pour chaque sujet pour prévenir l'apparition de phénomènes de fatigue (coût attentionnel) et d'apprentissage inhérents à la situation de double tâche. Chaque session d'évaluation comportera au total 16 mesures pour une durée de 8 minutes.

Tous les sujets réalisent ensuite, de façon identique une **tâche motrice standardisée de pédalage** sur ergocycle consistant à pédaler à 80% de la puissance maximale aérobie jusqu'à épuisement. Lors de ce test, la fréquence cardiaque, la tension artérielle ainsi que l'activité électrique du quadriceps (signal EMG) et l'oxygénation du quadriceps et du cerveau (NIRS) sont enregistrées en continu.

Immédiatement avant et après cet exercice fatigant est réalisé un protocole permettant de mesurer la modification d'excitabilité corticale et d'activation centrale induite par la tâche.

Un ergomètre amagnétique spécifique est utilisé pour ce protocole pré – post fatigue. Il permet de positionner la jambe droite de façon standardisée (angle du genou à 90°) alors que le sujet est assis et de mesurer la force d'extension au niveau de la cheville avec une jauge de contrainte reliée à un amplificateur et un système d'enregistrement. La réponse du cerveau avant et après l'exercice fatigant est évaluée d'une part par mesure du signal EMG de surface (calcul du root mean square (RMS) lors des contractions volontaires) et du signal NIRS musculaire et cérébral, et d'autre part par mesure des réponses mécaniques (amplitude de force évoquée) et EMG (amplitude du potentiel moteur évoqué) lors d'une stimulation corticale par TMS (cortex moteur, quelques centimètres à distance du vertex) ou lors d'une stimulation électrique (ENS) du nerf moteur (nerf fémoral au niveau du triangle fémoral) selon les techniques récemment décrites(37, 65, 75) et utilisées par notre équipe dans plusieurs protocoles de recherche clinique récents (protocoles EEMUR, CERVOX, VALLOT 2011).

L'activité EMG est enregistrée en continu par dérivation bipolaire en utilisant des électrodes de surface auto-adhésives. Les électrodes sont placées sur le vaste latéral selon les recommandations de la SENIAM(28).

Des stimulations électriques simples sont délivrées au niveau du nerf fémoral à l'aide d'un stimulateur (Digitimer DS7, Hertfordshire, UK). Des stimulations simples et doubles par TMS sont délivrées au niveau de la zone du cortex moteur correspondant aux extenseurs du genou à l'aide d'un stimulateur magnétique et d'une bobine en cône en forme de 8 (Magstim 200, Magstim Dyfeld, UK). Les zones de stimulation optimales au niveau du nerf moteur ainsi qu'au niveau cortical sont définies par ajustements successifs de l'électrode ou de la bobine de stimulation et mesure simultanée de la réponse mécanique et EMG. Le site de stimulation permettant d'obtenir la réponse la plus importante au niveau du muscle d'intérêt est marqué sur la peau du sujet afin de stimuler sur le même site tout au long du protocole. L'intensité de stimulation optimale est également définie lors de la stimulation du nerf moteur de façon à obtenir une stimulation supramaximale (120% de l'intensité permettant d'obtenir une réponse maximale) et lors de la stimulation magnétique corticale de façon à obtenir une onde M d'amplitude maximale lors d'une contraction à 50% de la force maximale volontaire (généralement de 40 à 60% de la puissance maximale du stimulateur).

Les stimulations électriques (NES) du nerf fémoral et magnétique (TMS) du cortex moteur permettent une mesure des propriétés contractiles des extenseurs du genou, de la transmission neuromusculaire (onde M), du niveau d'activation volontaire (par stimulation périphérique et corticale), des potentiels moteurs évoqués (MEP), de la période de silence corticale (SP) et de l'excitabilité des voies inhibitrices et excitatrices (par double stimulation TMS).

Après 10 min de mesures de repos, la force maximale du sujet est d'abord mesurée au cours d'un effort maximal. Ensuite, la réponse mécanique et EMG à une stimulation du nerf moteur ou de la zone du cortex moteur correspondant au muscle d'intérêt est mesurée à différents niveaux de contraction volontaire, à 0, 25, 50, 75 et 100% de la force maximale volontaire précédemment mesurée. Le sujet dispose d'un feedback visuel continu concernant l'intensité des contractions. Immédiatement après l'exercice de pédalage sur ergocycle, cette mesure sera répétée intégralement de façon à mesurer la modification d'excitabilité corticale et d'activation centrale induite par la tâche.

L'oxygénation cérébrale et musculaire est mesurée en transcutanée, en continu et de manière simultanée en utilisant un système multicanaux haute résolution (Oxymon, ARTINIS, Pays Bas) selon les techniques précédemment décrites(56, 63). Les modifications des concentrations en oxyhémoglobine (O_2Hb), désoxyhémoglobine (HHb) et hémoglobine totale (THb) musculaires et cérébrales sont ainsi enregistrées. Les sondes optiques NIRS, composées d'émetteurs et de récepteurs, sont fixées par adhésif double face sur la tête et la cuisse du sujet. Pour apprécier l'oxygénation cérébrale, la sonde de détection est positionnée au niveau de la zone corticale préfrontale, entre Fp1 et F3 et au niveau du cortex moteur centré en C1, selon le système international modifié EEG 10-20. Pour l'oxygénation musculaire, la sonde est placée au niveau du vaste latéral de la jambe droite, sur le ventre du muscle, parallèlement à la direction des fibres musculaires. Les données sont échantillonnées à une fréquence de 125 Hz. Une distance inter-optode de 4 cm est respectée. Cette technique est totalement indolore pour le patient.

Les investigateurs et équipes associées au présent projet ont une expertise importante quant aux techniques de stimulation magnétique transcranienne d'une part, le Pr Guillaume Millet et le Dr Bernard Wuyam revenant tous deux de séjours récents de formation l'un en Australie et l'autre à l'Hôpital Pitié Salepétrière, alors que Samuel Vergès et Thomas Rupp

utilisent cette technique depuis 2 ans pour des protocoles de recherche clinique au sein du CHU de Grenoble sur la fatigue centrale. De telles techniques diagnostiques sont utilisées dans l'exploration clinique de pathologies neurologiques ou dans l'exploration physiopathologique de certains états associés à une fatigue centrale. La stimulation nerveuse périphérique est maîtrisée au CHU de Grenoble en Physiologie & Rééducation depuis plusieurs années, sans effets indésirables. Concernant les méthodes de NIRS d'autre part, Stéphane Perrey et Thomas Rupp disposent d'une expérience de plusieurs années de réalisation de ce type de mesure dans différentes conditions et en particulier à l'effort physique et représentent à ce titre des experts de premier plan en Europe quant à ce type d'investigation.

6-5 VISITE 2 – Patients BPCO, SAOS et sujets contrôles (J1 + 1 semaine minimum : Tâche fatigante n°2 : extension de genou)

Cette visite se déroule de façon identique à la visite 1, la seule différence étant que la tâche fatigante est à présent un exercice d'extensions du genou réalisé en condition isométrique comprenant 5 secondes de contraction et 5 secondes de repos à 50% de la force maximale volontaire et maintenu jusqu'à épuisement. Le même ergomètre amagnétique sera utilisé que dans les conditions expérimentales décrites précédemment (visite 1).

Au cours de cette tâche, les signaux NIRS (vaste latéral et zone corticale préfrontale) et EMG (vaste latéral) sont mesurés en continu (selon la même méthodologie que décrite ci-dessus). **Immédiatement avant et après ainsi qu'à intervalle régulier pendant** cet exercice fatigant est réalisé le même protocole que lors de la première visite permettant de mesurer la modification d'excitabilité corticale et d'activation centrale induite par la tâche.

6-6 VISITE 3 Patients BPCO, SAOS et sujets contrôles (J2 + 2 jours minimum : Test de réponse ventilatoire et cérébro-vasculaire)

Cette visite débute par la réalisation d'un test de réponses ventilatoire et cérébro-vasculaire (évaluée par NIRS) à l'inhalation de différents mélanges gazeux avec des fractions d'O_2 et de CO_2 variables. Ce test doit permettre d'évaluer la chémosensibilité des patients au repos. Cette mesure de la réponse ventilatoire et cérébro-vasculaire sera basée sur les recommandations proposées récemment par Teppema et al.(71). Brièvement, le sujet est testé en décubitus dorsal, la tête surélevée inclinée à 30°. Le sujet est au repos, au calme, idéalement les yeux clos (réduction de la commande ventilatoire liée à l'éveil). Le sujet est installé dans une pièce sans bruit avec un casque antibruit sur la tête permettant de standardiser l'ambiance sonore. Les paramètres suivants sont mesurés via un embout buccal : la SaO_2, les pressions partielles de fin d'expiration en O_2 et CO_2 cycle par cycle ($P_{ET}CO_2$ et $P_{ET}O_2$), la ventilation (V_E). Le système permettant de délivrer un mélange gazeux avec une fraction inspirée d'O_2 et de CO_2 cible via un embout buccal est le même que celui permettant la réalisation de l'épreuve d'effort en hyperoxie lors des visites 4 et 5 pour les patients BPCO (Altitrainer, société SMTEC, Nyon, Suisse). Le sujet est d'abord maintenu à une $P_{ET}O_2$ de 100 mmHg pendant 10 min ; ensuite la $P_{ET}O_2$ est progressivement diminuée pendant 5 min pour atteindre 40-45 mmHg ; ce niveau de $P_{ET}O_2$ est alors maintenu pendant 5 min additionnelles. Cette procédure est répétée par 3 fois, avec une $P_{ET}CO_2$ maintenue à +2, +5 ou +12 mmHg par rapport à la valeur de repos en ventilation de repos en air ambiant. 15 min de repos en air ambiant sont respectées entre chaque mesure. Cette méthode permet d'une part de mesurer la réponse hypoxique aigue et d'autre part, du fait que cette réponse est obtenue à

différents niveaux de PCO_2, d'obtenir une caractérisation complète de l'interaction O_2-CO_2. Ce test a été utilisé lors d'un protocole précédemment mené par notre équipe (protocole VALLOT 2011) ainsi qu'en consultation de médecine de Montagne et est bien toléré.

6-7 VISITE 4 Patients BPCO (J2 + 1 semaine minimum : Tache fatigante n°1 : pédalage sur ergocycle avec inhalation d'un mélange hyperoxique)

Cette visite se déroule de façon identique à la visite 1, mais le sujet inhale cette fois un mélange gazeux hyperoxique (FiO_2 = 0.4). Le mélange gazeux avec une fraction d'oxygène de 40% est délivré au patient via un embout buccal par un système commercial destiné à la réalisation de telles épreuves d'effort en hypoxie ou hyperoxie utilisé lors des consultations de médecine du sport et de montagne (Altitrainer, société SMTEC, Nyon, Suisse).
L'ordre des visites 1 et 4 pour les patients BPCO est randomisé.

6-8 VISITE 5 Patients BPCO (J4 + 1 semaine minimum : Tache fatigante n°2 : extension de genou avec inhalation d'un mélange hyperoxique)

Cette visite se déroule de façon identique à la visite 2, mais le sujet inhale cette fois un mélange gazeux hyperoxique (FiO_2 = 0.4) comme lors de la visite 3.
L'ordre des visites 2 et 5 pour les patients BPCO est randomisé.

6-9 VISITE 4 Patients SAOS (Post traitement par PPC d'une durée de deux mois, J3 + 8 semaines minimum: évaluation de la locomotion et de la posture en air ambiant et Tache fatigante n°1 : pédalage sur ergocycle)

Cette visite prend place après le traitement par PPC et se déroule de façon identique à la visite 1.

6-10 VISITE 5 Patients SAOS (Post traitement par PPC d'une durée de deux mois, J4 + 1 semaine minimum : Tache fatigante n°2 : extension de genou)

Cette visite prend place après le traitement par PPC et se déroule de façon identique à la visite 2.

6-11 VISITE 6 Patients SAOS (Post traitement par PPC d'une durée de deux mois, J5 + 2 jours minimum : Test de réponse ventilatoire et cérébro-vasculaire)

Cette visite prend place après le traitement par PPC et se déroule de façon identique à la visite 3.

6-12 TRAITEMENT PAR PPC (patients SAOS)

Le traitement par PPC des patients SAOS nouvellement diagnostiqués sera réalisé au domicile du patient selon les modalités standards de prise en charge des patients SAOS et mis en place par un prestataire médico-technique à la demande du médecin investigateur. Un rapport d'observance à 1 mois et à 2 mois sera recueilli et permettra d'évaluer l'utilisation de l'appareillage par les patients.

6-13 PLANNING DES VISITES

Tableau synoptique de la chronologie des visites et des examens

6-13-1 Patients BPCO

Visites	J0	J1	J2	J3	J4	J5
Calendrier	J1 - 6 jours min	J1	J1 + 1 sem min	J2 + 2 j min	J2 + 1 sem min	J4 + 1 sem min
Remise de la note d'information	X					
Critères inclusion/exclusion; examen clinique	X					
Epreuve d'effort maximale	X					
Recueil du consentement	X					
Exploration fonctionnelle respiratoire	X	X	X	X	X	X
Test de réponse ventilatoire et cérébro-vasculaire				X		
Tâche motrice n°1: pédalage sur ergocycle		X			X	
Tâche motrice n°2: extensions de genou			X			X
Evaluations ENS, TMS, NIRS et EMG		X	X		X	X
Condition tâche motrice : air ambiant		X	X			
Condition tâche motrice hyperoxie (inhalation mélange gazeux)					X	X

6-13-2 Patients SAOS

Visites	J0	J1	J2	J3	J4	J5	J6
Calendrier	J1 - 6 jours min	J1	J1 + 1 sem min	J2 + 2 j min	J3 + 8 sem min	J4 + 1 sem min	J5 + 2 j min
Remise de la note d'information	X						
Critères inclusion/exclusion; examen clinique	X						
Epreuve d'effort maximale	X						
Recueil du consentement	X						
Test de réponse ventilatoire et cérébro-vasculaire				X			X
Examen neuropsychologique	X				X		
Evaluation de la marche et de la posture		X			X		
Tâche motrice: Pédalage sur ergocycle		X			X		
Tâche motrice : Extensions de genou			X			X	
Evaluations ENS, TMS, NIRS et EMG		X	X		X	X	

6-13-3 Sujets contrôles

Visites	J0	J1	J2	J3
Calendrier	J1 - 6 jours min	J1	J1 + 1 sem min	J2 + 2 j min
Remise de la note d'information	X			
Critères inclusion/exclusion; examen clinique	X			
Epreuve d'effort maximale	X			
Recueil du consentement	X			
Test de réponse ventilatoire et cérébro-vasculaire				X
Evaluation neuropsychologique	X		X	
Evaluation de la marche et de la posture		X	X	
Tâche motrice: Pédalage sur ergocycle		X		
Tâche motrice : Extensions de genou			X	
Evaluations ENS, TMS, NIRS et EMG		X	X	

6-14 ARRET PREMATURE DE L'ETUDE

Critères d'arrêt de l'étude pour un sujet qui y participe

- Sujet qui retire son consentement de participation à l'étude.

Arrêt de l'étude par le promoteur **:** Le promoteur peut arrêter l'étude à tout moment, pour les raisons suivantes :

- Incapacité de l'investigateur à inclure les sujets selon le calendrier prévu.
- Absence de consentement signé.
- Violations majeures au protocole.
- Données incomplètes ou erronées.

Arrêt de l'étude par l'investigateur : En cas d'événement indésirable jugé sévère par l'investigateur et pouvant mettre en jeu la santé des sujets, l'investigateur peut arrêter l'étude en accord avec le promoteur.

Un document sera transmis daté et signé par l'investigateur pour informer le promoteur de l'arrêt anticipé en indiquant les raisons.

6-15 CALENDRIER PREVISIONNEL

Temps nécessaire à la réalisation de l'étude :	24 mois
Date prévue pour le début de la recherche :	Avril 2012
Date prévue pour la fin de la recherche :	Avril 2014
Durée de participation pour un sujet :	7-8 semaines (BPCO)<br>13-14 semaines (SAOS)<br>3-4 semaines (sujets contrôles)

7 - GESTION DES DONNEES

Toutes les données des examens devront être recueillies dans un cahier de recueil des données individuelles et anonyme, spécifique au protocole, consultable par le promoteur et qui constituera les données sources pour cette étude.

Les cahiers d'observations seront remplis, par les investigateurs et collaborateurs scientifiques de cette étude (Mr Samuel Vergès et Mr Mathieu Gruet) et signés par l'investigateur.

Par son accord de participation, l'investigateur s'engage au strict respect du protocole expérimental, des "Bonnes Pratiques Cliniques" et de la législation en vigueur. Il se porte garant de l'authenticité des données recueillies dans le cadre de l'étude et accepte les

dispositions légales autorisant le Promoteur de l'étude à mettre en place un contrôle de la qualité.

L'identification des sujets sur ces cahiers sera faite exclusivement à l'aide des initiales des noms et prénoms des sujets et de leur numéro d'inclusion.

Un monitorage des données sera effectué sur les données recueillies correspondant aux critères primaires et secondaires par une Attaché de recherche Clinique au sein de l'UM Médecine du Sport & des Activités Physiques (Hôpital Sud), Mme Béatrice Leprohon, pour les données physiologiques.
L'ensemble des consentements sera vérifié, ainsi que les critères d'inclusion/non inclusion, et les évènements indésirables.
Le monitorage des données portera sur l'ensemble des cahiers d'observation. Un compte rendu de monitorage sera rédigé par l'ARC et conservé dans le dossier d'étude.

Les codes d'identification seront utilisés pour répertorier les données recueillies par informatique. Les données seront transférées sur PC dans des fichiers propres à l'étude. Une version papier de chacun des examens physiologiques (tests exercice) sera éditée et archivée.

En fin d'étude, les données seront archivées par l'investigateur coordonnateur dans l'UM Médecine du Sport & des Activités Physiques, Hôpital SUD, Avenue de Kimberley, 38434 Echirolles-Cedex.

L'analyse statistique ne sera effectuée qu'après vérification de la saisie et de la cohérence des données.

8 – ANALYSE STATISTIQUE

Il s'agira de comparer d'une part l'effet de la fatigue induite à l'exercice sur les différents paramètres de la fonction cérébrale entre les groupes de sujets et d'autre part l'effet d'interventions (supplémentation en O_2 ou traitement par PPC) sur ces paramètres. Les principaux paramètres analysés seront :

- Modifications d'oxyhémoglobine, de désoxyhémoglobine et d'hémoglobine totale entre le repos (avant effort) et l'effort (à un même temps d'effort, à une même intensité d'effort ou à effort maximal), ou en réponse à différents niveaux de fractions inspirés d'O_2 et de CO_2,
- Amplitude de MEP, des durées de SP et du niveau d'activation volontaire et leurs modifications entre avant et après les tâches fatigantes.

Les tests statistiques seront interprétés avec un risque de première espèce α fixé à 5% en situation bilatérale. L'analyse statistique sera réalisée par les collaborateurs experts de l'étude, avec le soutien de Mme Nathalie ARNOL, statisticienne au Laboratoire HP2 (recherche clinique).

Tous les sujets inclus dans cette étude constitueront la population saisie. Les sujets présentant une déviation majeure seront listés et ne seront pas inclus dans la population retenue pour l'analyse statistique. Les données non recueillies ne seront pas remplacées. Le sujet ne sera

pas exclu de l'étude, et une analyse des données disponibles sera réalisée sur les autres paramètres.

Les statistiques descriptives de chaque paramètre analysé seront les suivantes :

- Pour les variables quantitatives, les moyennes, écart-types, valeurs minimales et maximales, médianes, effectifs et nombres de valeurs manquantes seront présentés sous forme de tableaux.
- Pour les variables qualitatives, les effectifs, nombres de valeurs manquantes et pourcentages seront présentés dans les tableaux de contingence.

La comparaison des différences entre conditions sera effectuée :

- Pour les facteurs sous forme qualitative par un test du Chi2 ou un test exact de Fisher selon les conditions d'application du test du Chi2
- Pour les facteurs sous forme quantitative par analyse de variance (ANOVA) et test apparié de Student ou tests de Friedman et Wilcoxon.

Des corrélations seront recherchées en particulier entre modifications des gaz du sang et paramètres de la fonction cérébrale.

9 - ASPECTS MATERIELS ET LEGAUX

9-1 BALANCE BENEFICE / RISQUE

Les contraintes sont celles liées au fait de se rendre 3 à 6 fois à l'hôpital pour réaliser les différents tests pour une durée de 2-3 h à chaque visite. Les bénéfices pour le sujet sont inexistants de façon directe. Les patients BPCO et SAOS bénéficieront cependant d'un bilan individualisé de leur réponse cérébrale et musculaire à l'effort. Le bénéfice pour la collectivité est la connaissance des mécanismes de limitation de l'activité physique à l'hypoxie chronique et des adaptations physiologiques cérébrales en situation d'hypoxie et de demande énergétique forte liée à l'exercice. Les résultats de ce projet doivent avoir des retombées : **fondamentales** en offrant une description détaillée de la réponse cérébrale au repos et à l'effort, lors d'efforts de la vie courante (marche et posture) et au cours d'exercice plus intenses de patients hypoxémiques et **pratiques** en analysant la pertinence de stratégies luttant contre l'hypoxémie artérielle (supplémentation en O_2 chez le patient BPCO et PPC chez le patient SAOS) pour réduire les effets de l'hypoxie chronique sur le cerveau et l'intolérance à l'exercice physique. Les risques sont ceux associées à la pratique d'un exercice physique comme l'intolérance hémodynamique qui seront prévenus par le strict respect des critères de non-inclusion, le dépistage préalable des intolérances et anomalies cardio-vasculaires au cours des tests d'effort réalisés lors de la visite d'inclusion, le monitorage de la FC, SpO2 et TA au cours de l'exercice, la disponibilité à proximité des moyens de prise en charge d'urgence du CHU, et toute procédure adaptée au sein du CHU sous la responsabilité des investigateurs. Ces tests et leurs conditions de réalisation correspondent aux évaluations approfondies de la réponse physiologique à l'effort telle qu'elles sont réalisées quotidiennement en consultation au laboratoire d'Exploration Fonctionnelle Cardio-Respiratoire et en Médecine du Sport (1100 épreuves d'effort maximal par an CHU de Grenoble).

Les effets secondaires prévisibles sont :
- Des courbatures au niveau du quadriceps du fait des tâches motrices, ainsi que des exercices de marche et de posture.
- Un inconfort lors de l'application de la stimulation électrique; cette application est peu douloureuse, la survenue d'une contraction musculaire involontaire (lors de la stimulation) créant un léger désagrément,
- Des céphalées suite à l'application de la stimulation magnétique corticale ; l'application de stimulation unique sous-maximale (et non pas 'stimulation répétitive à haute fréquence' comme dans le cadre des traitements de certaines pathologies neurologiques par exemple) rend minimal le risque de ce type d'effet secondaire.
- Une sensation de fatigue à l'issue des tests de marche et posture, légère sur le plan physique liée à la marche ou la posture, mais également intellectuelle, liée à l'évaluation neuropsychologique ainsi qu'à la condition de double tâche (marcher et analyser, se maintenir et analyser).
- Des risques de déséquilibre pouvant aller jusqu'à la chute lors des tests de posture ou lors de l'évaluation à la marche. Elles seront prévenues par une sécurité passive sur le tapis (harnais) et des barrières de sécurité.

9-2 CONSENTEMENT DU PATIENT

Conformément aux Bonnes Pratiques Cliniques et aux dispositions légales en vigueur, tout sujet présélectionné sera préalablement informé par l'investigateur des objectifs de l'étude, de sa méthodologie, de sa durée, de ses contraintes et des risques prévisibles, des alternatives thérapeutiques possibles, et des modalités de prise en charge médicale prévues en fin de recherche y compris en cas d'arrêt de l'étude avant son terme. Il sera notamment précisé au sujet qu'il est entièrement libre de refuser de participer à l'étude ou de retirer son consentement à tout moment sans encourir aucune responsabilité ni aucun préjudice de ce fait. Une Lettre d'Information résumant les renseignements donnés par l'investigateur et le formulaire de consentement lui seront remis lors de la visite de sélection initiale.
Après s'être assuré de la bonne compréhension des informations fournies, l'investigateur sollicitera de la part du sujet, lors de la première visite de sélection (J0), son consentement écrit pour participer à l'étude. S'il accepte, le sujet signera le formulaire de consentement préalablement à la réalisation de l'étude.

9-3 PROTECTION DES PERSONNES

Le protocole de cette étude a reçu un avis favorable du Comité de Protection des Personnes Sud-Est V de Grenoble, le …………………… et l'autorisation de l'AFSAPS le ....

Tel qu'exigé par le code de la santé publique, le rapport bénéfice/risque est favorable pour les personnes qui s'y prêtent. Cette étude sera précédée d'un examen médical des sujets impliqués et les résultats de cet examen leur seront communiqués préalablement à l'expression de leur consentement par l'intermédiaire du médecin de leur choix. Tous les participants à l'étude seront affiliés à un régime de sécurité sociale. Aucune recherche ne sera effectuée sur une personne sans son accord préalable.

9-4 COUTS POUR LE PATIENT

Pendant la durée de l'étude, il ne sera facturé aucun frais au sujet pour la totalité des examens prévus au protocole.

9-5 INFORMATIQUE ET LIBERTE

Un traitement informatisé des données recueillies dans cette étude sera réalisé de façon strictement **ANONYME**.

Le présent protocole se propose de bénéficier de la Déclaration Simplifiée MR 001.

Afin de protéger les libertés individuelles, la loi prévoit que :

- Le sujet dispose d'un droit d'accès aux informations le concernant contenues dans ce fichier informatique, et d'un droit de rectification le cas échéant et de la vérification de leur destruction au terme du délai d'un an prévu par l'article R.2045.
- Ce droit d'accès aux informations le concernant s'exerce soit auprès d'un médecin de son choix soit directement. (Depuis la loi de mars 2002).
- Ce droit d'accès s'exerce au Centre Hospitalier de Grenoble, et toute rectification éventuelle sera effectuée dans la journée ouvrable suivant sa demande.
- Ces données extraites du fichier informatisé seront exploitées selon des critères de stricte confidentialité, sauf opposition de la part du participant, formulée auprès du médecin de son centre diagnostique.

9-6 ACCES AUX RESULTATS

Les sujets peuvent avoir un accès direct aux résultats de l'examen médical (cf. Article L1121-11 du Code la Santé Publique.

L'accès aux informations contenues dans le dossier médical est possible directement par le patient depuis la loi n° 2002-303 du 4 mars 2002.

Au terme de cette recherche, le CHU de Grenoble mettra à la disposition des sujets les résultats globaux de l'étude. S'il le souhaite, le sujet pourra être informé de ces résultats sur simple demande auprès du médecin investigateur de son choix.

9-7 CONTRAT D'ASSURANCE

Le promoteur de cette étude a souscrit un contrat d'assurance auprès de la Société Hospitalière d'Assurance Mutuelle (SHAM) – 18 rue Edouard Rochet 69372 LYON cedex 08, sous le numéro : 135 751

9-8 SECRET PROFESSIONNEL- CONFIDENTIALITE

Le sujet se prêtant à la recherche Biomédicale du présent protocole autorise l'accès de ses données uniquement aux personnes en charge de l'investigation. L'investigateur est tenu au respect du secret professionnel. Les données recueillies, y compris les résultats des analyses, seront rendues anonymes par tout moyen approprié. Le promoteur et ses représentants sont soumis aux mêmes obligations de secret professionnel que l'investigateur.

Le présent document et ses annexes ne doivent être remis ou communiqués qu'aux personnes nommément impliquées dans l'essai avec l'accord ou à la demande de l'investigateur coordonnateur.

9-9 PUBLICATIONS

Toutes les données recueillies au cours de cette étude sont la propriété du promoteur de l'étude et ne peuvent être communiquées en aucun cas à une tierce personne sans l'accord écrit de l'investigateur et du Promoteur. Toute publication ou communication (orale ou écrite) sera décidée d'un commun accord entre les investigateurs et respectera les recommandations internationales: "Uniforms Requirements for Manuscripts Submitted to Biomedical Journals" (http://www.cma.ca/publications/mwc/uniform.htm). L'étude sera enregistrée dans le répertoire public 'Clinical trial' afin d'éviter les biais de publications.

9-10 ARCHIVAGE

L'ensemble des dossiers de l'étude sera archivé pour une durée de 15 ans, sous la responsabilité du promoteur. Les documents source, les cahiers d'observation, les originaux des formulaires de consentement, le protocole signé devront être conservés par l'investigateur pendant la durée minimale de 15 ans à compter de la fin de l'étude.
L'investigateur coordonnateur organise, au nom du promoteur de l'étude, le stockage dans des locaux appropriés (au sein de l'UM Médecine du Sport & des Activités Physiques, Hôpital SUD, Avenue de Kimberley, 38434 Echirolles-Cedex) les documents suivants :

- Protocole avec annexes, amendements.
- Cahiers d'observation (originaux) avec documents annexes.
- Document de suivi de l'étude clinique.
- Toutes les pièces administratives et correspondances liées à l'étude.
- Rapport d'étude.

10 - DATES ET SIGNATURES

Ce protocole a été lu et approuvé à la date notée en en-tête

Investigateur coordonnateur

Pr Patrick LEVY

Laboratoire EFCR

CHU de Grenoble

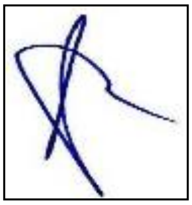

Pour le promoteur

Mme Hélène SABBAH-GUILLAUME

Direction de la Recherche Clinique et de l'Innovation

CHU de Grenoble

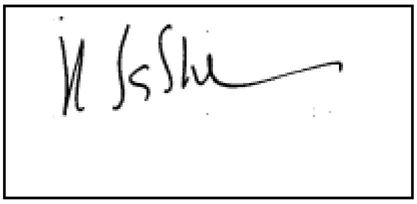

11 - REFERENCES BILIOGRAPHIQUES

1. **Ainslie PN, and Poulin MJ**. Ventilatory, cerebrovascular, and cardiovascular interactions in acute hypoxia: regulation by carbon dioxide. *J Appl Physiol* 97: 149-159, 2004.
2. **Amann M, Romer LM, Subudhi AW, Pegelow DF, and Dempsey JA**. Severity of arterial hypoxaemia affects the relative contributions of peripheral muscle fatigue to exercise performance in healthy humans. *J Physiol* 581: 389-403, 2007.
3. **Astrand PO, Cuddy TE, Saltin B, and Stenberg J**. Cardiac Output during Submaximal and Maximal Work. *J Appl Physiol* 19: 268-274, 1964.
4. **Barnes M, Goldsworthy UR, Cary BA, and Hill CJ**. A diet and exercise program to improve clinical outcomes in patients with obstructive sleep apnea--a feasibility study. *J Clin Sleep Med* 5: 409-415, 2009.
5. **Bassett DR, Jr., and Howley ET**. Limiting factors for maximum oxygen uptake and determinants of endurance performance. *Med Sci Sports Exerc* 32: 70-84, 2000.
6. **Bedard MA, Montplaisir J, Richer F, Rouleau I, and Malo J**. Obstructive sleep apnea syndrome: pathogenesis of neuropsychological deficits. *J Clin Exp Neuropsychol* 13: 950-964, 1991.
7. **Beebe DW, Groesz L, Wells C, Nichols A, and McGee K**. The neuropsychological effects of obstructive sleep apnea: a meta-analysis of norm-referenced and case-controlled data. *Sleep* 26: 298-307, 2003.
8. **Bergh U, Ekblom B, and Astrand PO**. Maximal oxygen uptake "classical" versus "contemporary" viewpoints. *Med Sci Sports Exerc* 32: 85-88, 2000.
9. **Bhambhani Y, Malik R, and Mookerjee S**. Cerebral oxygenation declines at exercise intensities above the respiratory compensation threshold. *Respir Physiol Neurobiol* 156: 196-202, 2007.
10. **Blomstrand E**. Amino acids and central fatigue. *Amino Acids* 20: 25-34, 2001.
11. **Brugniaux JV, Hodges AN, Hanly PJ, and Poulin MJ**. Cerebrovascular responses to altitude. *Respir Physiol Neurobiol* 158: 212-223, 2007.
12. **Calbet JA, Boushel R, Radegran G, Sondergaard H, Wagner PD, and Saltin B**. Why is VO2 max after altitude acclimatization still reduced despite normalization of arterial O2 content? *Am J Physiol Regul Integr Comp Physiol* 284: R304-316, 2003.
13. **Canessa N, Castronovo V, Cappa SF, Aloia MS, Marelli S, Falini A, Alemanno F, and Ferini-Strambi L**. Obstructive sleep apnea: brain structural changes and neurocognitive function before and after treatment. *Am J Respir Crit Care Med* 183: 1419-1426.
14. **Cannizzaro G, Garbin L, Clivati A, and Pesce LI**. Correction of hypoxia and hypercapnia in COPD patients: effects on cerebrovascular flow. *Monaldi Arch Chest Dis* 52: 9-12, 1997.
15. **Celli BR**. Predictors of mortality in COPD. *Respir Med* 104: 773-779, 2010.
16. **Chaudhuri A, and Behan PO**. Fatigue and basal ganglia. *J Neurol Sci* 179: 34-42, 2000.
17. **Chien MY, Wu YT, Lee PL, Chang YJ, and Yang PC**. Inspiratory muscle dysfunction in patients with severe obstructive sleep apnoea. *Eur Respir J* 35: 373-380, 2010.
18. **Dalsgaard MK**. Fuelling cerebral activity in exercising man. *J Cereb Blood Flow Metab* 26: 731-750, 2006.
19. **Davis JM, Alderson NL, and Welsh RS**. Serotonin and central nervous system fatigue: nutritional considerations. *Am J Clin Nutr* 72: 573S-578S, 2000.

20. **Foster GE, Hanly PJ, Ostrowski M, and Poulin MJ**. Effects of continuous positive airway pressure on cerebral vascular response to hypoxia in patients with obstructive sleep apnea. *Am J Respir Crit Care Med* 175: 720-725, 2007.
21. **Gonzalez-Alonso J, Dalsgaard MK, Osada T, Volianitis S, Dawson EA, Yoshiga CC, and Secher NH**. Brain and central haemodynamics and oxygenation during maximal exercise in humans. *J Physiol* 557: 331-342, 2004.
22. **Goodall S, Ross EZ, and Romer LM**. Effect of graded hypoxia on supraspinal contributions to fatigue with unilateral knee-extensor contractions. *J Appl Physiol* 109: 10, 2010.
23. **Goodall S, Ross EZ, and Romer LM**. Effect of graded hypoxia on supraspinal contributions to fatigue with unilateral knee-extensor contractions. *J Appl Physiol* 109: 1842-1851, 2010.
24. **Guezennec CY, Abdelmalki A, Serrurier B, Merino D, Bigard X, Berthelot M, Pierard C, and Peres M**. Effects of prolonged exercise on brain ammonia and amino acids. *Int J Sports Med* 19: 323-327, 1998.
25. **Haddad GG, and Jiang C**. O2 deprivation in the central nervous system: on mechanisms of neuronal response, differential sensitivity and injury. *Prog Neurobiol* 40: 277-318, 1993.
26. **Hansen AJ**. Effect of anoxia on ion distribution in the brain. *Physiol Rev* 65: 101-148, 1985.
27. **Herman T, Mirelman A, Giladi N, Schweiger A, and Hausdorff JM**. Executive control deficits as a prodrome to falls in healthy older adults: a prospective study linking thinking, walking, and falling. *J Gerontol A Biol Sci Med Sci* 65: 1086-1092.
28. **Hermens HJ, Freriks B, Disselhorst-Klug C, and Rau G**. Development of recommendations for SEMG sensors and sensor placement procedures. *J Electromyogr Kinesiol* 10: 361-374, 2000.
29. **Hopkinson NS, Sharshar T, Ross ET, Nickol AH, Dayer MJ, Porcher R, Jonville S, Moxham J, and Polkey MI**. Corticospinal control of respiratory muscles in chronic obstructive pulmonary disease. *Respir Physiol Neurobiol* 141: 1-12, 2004.
30. **Ide K, and Secher NH**. Cerebral blood flow and metabolism during exercise. *Prog Neurobiol* 61: 397-414, 2000.
31. **Jackson MJ**. Molecular mechanisms of muscle damage. *Mol Cell Biol Hum Dis Ser* 3: 257-282, 1993.
32. **Jensen G, Nielsen HB, Ide K, Madsen PL, Svendsen LB, Svendsen UG, and Secher NH**. Cerebral oxygenation during exercise in patients with terminal lung disease. *Chest* 122: 445-450, 2002.
33. **Kayser B**. Exercise starts and ends in the brain. *Eur J Appl Physiol* 90: 411-419, 2003.
34. **Kayser B, Narici M, Binzoni T, Grassi B, and Cerretelli P**. Fatigue and exhaustion in chronic hypobaric hypoxia: influence of exercising muscle mass. *J Appl Physiol* 76: 634-640, 1994.
35. **Kent-Braun JA**. Central and peripheral contributions to muscle fatigue in humans during sustained maximal effort. *Eur J Appl Physiol Occup Physiol* 80: 57-63, 1999.
36. **Lal C, Strange C, and Bachman D**. Neurocognitive impairment in obstructive sleep apnea. *Chest* 141: 1601-1610.
37. **Lee M, Gandevia SC, and Carroll TJ**. Cortical voluntary activation can be reliably measured in human wrist extensors using transcranial magnetic stimulation. *Clin Neurophysiol* 119: 1130-1138, 2008.
38. **Lepers R, Maffiuletti NA, Rochette L, Brugniaux J, and Millet GY**. Neuromuscular fatigue during a long-duration cycling exercise. *J Appl Physiol* 92: 1487-1493, 2002.

39. **Levy P, Tamisier R, Minville C, Launois S, and Pepin JL**. Sleep apnoea syndrome in 2011: current concepts and future directions. *Eur Respir Rev* 20: 134-146.

40. **Loeppky JA, Miranda FG, and Eldridge MW**. Abnormal cerebrovascular responses to CO2 in sleep apnea patients. *Sleep* 7: 97-109, 1984.

41. **Macey PM, Henderson LA, Macey KE, Alger JR, Frysinger RC, Woo MA, Harper RK, Yan-Go FL, and Harper RM**. Brain morphology associated with obstructive sleep apnea. *Am J Respir Crit Care Med* 166: 1382-1387, 2002.

42. **Mayer P, Dematteis M, Pepin JL, Wuyam B, Veale D, Vila A, and Levy P**. Peripheral neuropathy in sleep apnea. A tissue marker of the severity of nocturnal desaturation. *Am J Respir Crit Care Med* 159: 213-219, 1999.

43. **Mazza S, Pepin JL, Naegele B, Plante J, Deschaux C, and Levy P**. Most obstructive sleep apnoea patients exhibit vigilance and attention deficits on an extended battery of tests. *Eur Respir J* 25: 75-80, 2005.

44. **Miscio G, Milano E, Aguilar J, Savia G, Foffani G, Mauro A, Mordillo-Mateos L, Romero-Ganuza J, and Oliviero A**. Functional involvement of central nervous system at high altitude. *Exp Brain Res* 194: 157-162, 2009.

45. **Mohamed-Hussein AA, Hamed SA, and Abdel-Hakim N**. Cerebral cortical dysfunction in chronic obstructive pulmonary disease: role of transcranial magnetic stimulation. *Int J Tuberc Lung Dis* 11: 515-521, 2007.

46. **Morrell MJ, and Twigg G**. Neural consequences of sleep disordered breathing: the role of intermittent hypoxia. *Adv Exp Med Biol* 588: 75-88, 2006.

47. **Naegele B, Thouvard V, Pepin JL, Levy P, Bonnet C, Perret JE, Pellat J, and Feuerstein C**. Deficits of cognitive executive functions in patients with sleep apnea syndrome. *Sleep* 18: 43-52, 1995.

48. **Nielsen HB, Boushel R, Madsen P, and Secher NH**. Cerebral desaturation during exercise reversed by O2 supplementation. *Am J Physiol* 277: H1045-1052, 1999.

49. **Noakes TD**. Physiological models to understand exercise fatigue and the adaptations that predict or enhance athletic performance. *Scand J Med Sci Sports* 10: 123-145, 2000.

50. **Noakes TD, Peltonen JE, and Rusko HK**. Evidence that a central governor regulates exercise performance during acute hypoxia and hyperoxia. *J Exp Biol* 204: 3225-3234, 2001.

51. **Nybo L, Moller K, Pedersen BK, Nielsen B, and Secher NH**. Association between fatigue and failure to preserve cerebral energy turnover during prolonged exercise. *Acta Physiol Scand* 179: 67-74, 2003.

52. **O'Donoghue FJ, Wellard RM, Rochford PD, Dawson A, Barnes M, Ruehland WR, Jackson ML, Howard ME, Pierce RJ, and Jackson GD**. Magnetic resonance spectroscopy and neurocognitive dysfunction in obstructive sleep apnea before and after CPAP treatment. *Sleep* 35: 41-48.

53. **Oliviero A, Corbo G, Tonali PA, Pilato F, Saturno E, Dileone M, Versace V, Valente S, and Di Lazzaro V**. Functional involvement of central nervous system in acute exacerbation of chronic obstructive pulmonary disease A preliminary transcranial magnetic stimulation study. *J Neurol* 249: 1232-1236, 2002.

54. **Ozaki H, Watanabe S, and Suzuki H**. Topographic EEG changes due to hypobaric hypoxia at simulated high altitude. *Electroencephalogr Clin Neurophysiol* 94: 349-356, 1995.

55. **Papadelis C, Kourtidou-Papadeli C, Bamidis PD, Maglaveras N, and Pappas K**. The effect of hypobaric hypoxia on multichannel EEG signal complexity. *Clin Neurophysiol* 118: 31-52, 2007.

56. **Perrey S**. Non-invasive NIR spectroscopy of human brain function during exercise. *Methods* 45: 289-299, 2008.

57. **Rasmussen P, Dawson EA, Nybo L, van Lieshout JJ, Secher NH, and Gjedde A**. Capillary-oxygenation-level-dependent near-infrared spectrometry in frontal lobe of humans. *J Cereb Blood Flow Metab* 27: 1082-1093, 2007.
58. **Rasmussen P, Nielsen J, Overgaard M, Krogh-Madsen R, Gjedde A, Secher NH, and Petersen NC**. Reduced muscle activation during exercise related to brain oxygenation and metabolism in humans. *J Physiol* 588: 1985-1995, 2010.
59. **Reichmuth KJ, Dopp JM, Barczi SR, Skatrud JB, Wojdyla P, Hayes D, Jr., and Morgan BJ**. Impaired vascular regulation in patients with obstructive sleep apnea: effects of continuous positive airway pressure treatment. *Am J Respir Crit Care Med* 180: 1143-1150, 2009.
60. **Remsburg S, Launois SH, and Weiss JW**. Patients with obstructive sleep apnea have an abnormal peripheral vascular response to hypoxia. *J Appl Physiol* 87: 1148-1153, 1999.
61. **Ries AL**. Pulmonary rehabilitation: summary of an evidence-based guideline. *Respiratory care* 53: 1203-1207, 2008.
62. **Rupp T, and Perrey S**. Effect of severe hypoxia on prefrontal and muscle oxygentaion responses at rest and during isometric exhausting exercise. *Adv Exp Med Biol* 645: 329-334, 2009.
63. **Rupp T, and Perrey S**. Effect of severe hypoxia on prefrontal and muscle oxygentaion responses at rest and during isometric exhausting exercise. *Adv Exp Med Biol* in press: 2009.
64. **Sassoon CS, Gruer SE, and Sieck GC**. Temporal relationships of ventilatory failure, pump failure, and diaphragm fatigue. *J Appl Physiol* 81: 238-245, 1996.
65. **Sidhu SK, Bentley DJ, and Carroll TJ**. Locomotor exercise induces long-lasting impairments in the capacity of the human motor cortex to voluntarily activate knee extensor muscles. *J Appl Physiol* 106: 556-565, 2009.
66. **Subudhi AW, Dimmen AC, and Roach RC**. Effects of acute hypoxia on cerebral and muscle oxygenation during incremental exercise. *J Appl Physiol* 103: 177-183, 2007.
67. **Subudhi AW, Lorenz MC, Fulco CS, and Roach RC**. Cerebrovascular responses to incremental exercise during hypobaric hypoxia: effect of oxygenation on maximal performance. *Am J Physiol Heart Circ Physiol* 294: H164-171, 2008.
68. **Szubski C, Burtscher M, and Loscher WN**. The effects of short-term hypoxia on motor cortex excitability and neuromuscular activation. *J Appl Physiol* 101: 1673-1677, 2006.
69. **Szubski C, Burtscher M, and Loscher WN**. Neuromuscular fatigue during sustained contractions performed in short-term hypoxia. *Med Sci Sports Exerc* 39: 948-954, 2007.
70. **Taylor JL, Allen GM, Butler JE, and Gandevia SC**. Supraspinal fatigue during intermittent maximal voluntary contractions of the human elbow flexors. *J Appl Physiol* 89: 305-313, 2000.
71. **Teppema LJ, and Dahan A**. The ventilatory response to hypoxia in mammals: mechanisms, measurement, and analysis. *Physiol Rev* 90: 675-754, 2010.
72. **Tuunanen PI, and Kauppinen RA**. Effects of oxygen saturation on BOLD and arterial spin labelling perfusion fMRI signals studied in a motor activation task. *Neuroimage* 30: 102-109, 2006.
73. **Van de Ven MJ, Colier WN, Van der Sluijs MC, Kersten BT, Oeseburg B, and Folgering H**. Ventilatory and cerebrovascular responses in normocapnic and hypercapnic COPD patients. *Eur Respir J* 18: 61-68, 2001.
74. **van de Ven MJ, Colier WN, van der Sluijs MC, Oeseburg B, Vis P, and Folgering H**. Effects of acetazolamide and furosemide on ventilation and cerebral blood volume in normocapnic and hypercapnic patients with COPD. *Chest* 121: 383-392, 2002.

75. **Verges S, Maffiuletti NA, Kerherve H, Decorte N, Wuyam B, and Millet GY**. Comparison of electrical and magnetic stimulations to assess quadriceps muscle function. *J Appl Physiol* 106: 701-710, 2009.

76. **Vivodtzev I, Flore P, Levy P, and Wuyam B**. Voluntary activation during knee extensions in severely deconditioned patients with chronic obstructive pulmonary disease: benefit of endurance training. *Muscle Nerve* 37: 27-35, 2008.

77. **Volianitis S, Fabricius-Bjerre A, Overgaard A, Stromstad M, Bjarrum M, Carlson C, Petersen NT, Rasmussen P, Secher NH, and Nielsen HB**. The cerebral metabolic ratio is not affected by oxygen availability during maximal exercise in humans. *J Physiol* 586: 107-112, 2008.

78. **Wagner PD**. Reduced maximal cardiac output at altitude--mechanisms and significance. *Respir Physiol* 120: 1-11, 2000.

79. **Westerblad H, Allen DG, Bruton JD, Andrade FH, and Lannergren J**. Mechanisms underlying the reduction of isometric force in skeletal muscle fatigue. *Acta Physiol Scand* 162: 253-260, 1998.

80. **Woollacott M, and Shumway-Cook A**. Attention and the control of posture and gait: a review of an emerging area of research. *Gait Posture* 16: 1-14, 2002.

81. **Yogev-Seligmann G, Hausdorff JM, and Giladi N**. The role of executive function and attention in gait. *Mov Disord* 23: 329-342; quiz 472, 2008.

12 - ANNEXES

12-1 NOTES D'INFORMATION AU PATIENT

12-1-1 Patients BPCO

==

NOTE D'INFORMATION AU PATIENT BPCO

==

Document constitué en application du Code de Santé Publique

Investigateur :
Pr. Patrick LEVY
Laboratoire EFCR, CHU de Grenoble
BP 217
38043 GRENOBLE cedex
Tél. : +33 0(4) 76 76 55 16

Promoteur : CHU de Grenoble

Titre identifiant la recherche :

Le cerveau à l'effort: effets de l'hypoxie chez le malade respiratoire

Le Dr. .... m'a proposé de participer à une recherche organisée par le C.H.U. de GRENOBLE portant sur une étude physiopathologique comportant une série d'évaluations du cerveau et des muscles au repos et à l'effort en respirant l'air ambiant ou un mélange gazeux enrichi en oxygène.

Il m'a précisé que j'ai le droit de refuser de participer à cette recherche ou de retirer mon consentement, à tout moment sans encourir aucune responsabilité ni aucun préjudice de ce fait (article L1122-1 du code de la santé publique). Les données recueillies précédemment à cet arrêt seront utilisées sauf si je ne le souhaite pas. Je suis donc libre d'accepter ou de refuser. Cela ne changera pas ma prise en charge habituelle. J'ai bien reçu et j'ai bien compris les informations suivantes.

Aucun frais ne sera facturé du fait de la participation à cette étude.

BUT DE L'ETUDE

Il s'agit d'évaluer l'effet de l'hypoxie (manque d'oxygène dans le sang) chronique caractéristique des maladies respiratoires telles que la BPCO ou le syndrome d'apnée du sommeil, sur les perturbations cérébrales associées à la réalisation d'un exercice fatigant. L'objectif de l'étude est d'évaluer l'oxygénation, la perfusion et l'activation du cerveau, la réponse des neurones, ainsi que la commande du cerveau vers les muscles avant, pendant et après un exercice physique fatigant réalisé en respirant l'air ambiant ou un mélange gazeux enrichi en oxygène.

METHODOLOGIE

Au cours d'une visite préalable dite de sélection, une vérification des critères d'inclusion ainsi qu'un examen clinique seront réalisés. L'investigateur vous remettra cette lettre d'information accompagnée du formulaire de consentement.

Si vous acceptez de participer, vous serez évalué en cinq occasions. Chacune sera espacée d'au moins 2 jours d'intervalle. Chaque session (hormis la session 3 qui sera sans effort) consistera en la réalisation d'un exercice standardisé de la jambe, suffisamment intense pour fatiguer votre muscle. L'exercice sera réalisé en position assise sur une chaise spéciale ou sur une bicyclette. Avant et après ces exercices fatigants seront réalisées des mesures par stimulation magnétique d'une partie de votre cerveau correspondant à la commande de votre jambe.

DEROULEMENT DE L'ETUDE

Un bilan médical préalable à l'étude sera réalisé. Les résultats de cet examen vous seront communiqués à l'issue de la visite de sélection par le médecin de votre choix. Le déroulement global de cette étude sera organisé en cinq visites à l'hôpital de 2-3 h chacune, séparées d'au moins deux jours.

Le détail des examens réalisés à l'hôpital vous est présenté ci-après :

Sur un plan pratique :

Vous serez convoqué pour chaque visite au CHU de Grenoble.

Lors de la **première visite de sélection**, vous serez convoqué à l'UF Recherche sur l'Exercice du CHU de Grenoble. Après vérification de la signature du consentement, un examen clinique et les mesures anthropométriques (taille, poids, etc) seront réalisés. Ensuite, vous réaliserez un test d'effort sur bicyclette : ce test consiste à pédaler de façon continue alors que vous respirez librement dans un embout permettant de mesurer votre souffle. L'intensité de l'effort augmentera progressivement et des encouragements verbaux vous seront donnés jusqu'à ce que vous ne parveniez plus à pédaler. Régulièrement au cours de l'effort, il vous sera demandé d'évaluer sur une échelle de 1 à 10 l'intensité de votre essoufflement et l'intensité de votre fatigue des jambes. A la fin de ce test il sera possible de vous communiquer la puissance maximale que vous avez développée sur la bicyclette.

Suite à cette visite, **5 autres visites au total seront réalisées**, chacune espacées d'au moins deux semaines. Elles se dérouleront toutes à l'UF Recherche sur l'exercice.

La première de ces quatre visites consistera à l'évaluation de votre fonction cérébrale avant pendant et après un effort sur bicyclette réalisé à 80% de votre puissance maximale maintenu jusqu'à épuisement.
Avant et après cet effort fatigant, votre quadriceps sera artificiellement stimulé par un courant électrique qui sera appliqué juste en amont du muscle sur le nerf fémoral ou par une stimulation magnétique qui sera appliquée au niveau de votre crâne sur la zone de votre cerveau correspondant au muscle concerné. Ce genre de stimulation est sans douleur et induit une contraction involontaire de faible amplitude. Afin de mesurer la réaction de votre muscle, des électrodes seront placées sur la peau. Votre fréquence cardiaque, votre tension artérielle ainsi que l'oxygénation de votre cerveau et de votre cuisse seront mesurées et contrôlées en continu.

Lors de la seconde visite, nous procéderons à l'évaluation de votre fonction cérébrale avant et pendant et après un exercice d'extensions du genou, votre genou étant fléchi à 90°, la contraction de votre cuisse devant tenter d'étendre votre jambe droite alors que votre cheville

sera fixée à un capteur de force. L'exercice consistera en 5 secondes d'effort (à un niveau prédéterminé de 50% de votre force maximale préalablement mesurée et qui vous sera indiqué par un signal visuel) suivi de 5 secondes de relâchement, jusqu'à épuisement. Avant, pendant et après cet effort fatigant seront réalisés les mêmes tests que lors de la première visite.

Lors de troisième visite, vous serez installé dans un fauteuil et vous respirerez à travers un embout buccal des mélanges gazeux avec différents contenus d'oxygène et de dioxyde de carbone. Ce test durera 1h30 environ, vous aurez simplement à rester éveillé et calme.

Lors de la quatrième visite, vous réaliserez les mêmes tests que lors de la première, à la différence que vous respirerez un mélange gazeux enrichi en oxygène.

Lors de la cinquième visite, vous réaliserez les mêmes tests que lors de la deuxième, à la différence que vous respirerez un mélange gazeux enrichi en oxygène.

L'ordre des visites 1 et 4 et des visites 2 et 5 sera tiré au sort.

BENEFICES

Aucun bénéfice individuel direct n'est attendu. Les tests réalisés vous permettront cependant d'avoir plusieurs indices relatifs à votre tolérance à l'effort, à l'adaptation de votre cerveau et de vos muscles à l'effort.

DUREE DE L'ETUDE

Vous serez inclus dans l'étude pour une durée variable allant de 7 à 8 semaines.

Vous êtes libre de participer à une autre étude clinique durant votre inclusion dans le présent projet dans la mesure où celle-ci n'interfère pas avec cette recherche.

CONTRAINTES ET RISQUES

Les contraintes sont celles liées au fait de se rendre cinq fois à l'hôpital pour réaliser les différents tests.

Les effets secondaires prévisibles sont :
- Des courbatures au niveau du quadriceps du fait du pédalage,
- Un inconfort lors de l'application de la stimulation électrique ou magnétique; l'application de la stimulation électrique est peu douloureuse, la survenue d'une contraction musculaire involontaire (lors de la stimulation) créant un léger désagrément ; l'application d'une stimulation magnétique de votre cerveau n'est pas douloureuse, mais peu éventuellement causer la survenue de céphalées.

Les risques prévisibles sont ceux liés aux tests à l'effort : ces tests sont réalisés par un médecin et un expérimentateur ou technicien expérimenté. Très rarement, les tests à l'effort sont associés à des complications cardiaques ou des baisses de tension qui pourraient être graves, et qui sont prévenues par l'application de procédures de surveillance et d'arrêt

anticipé de l'exercice strictes. Aucune complication de ce type n'est survenue sur ce lieu de recherche considéré du CHU de Grenoble, ces tests étant régulièrement pratiqués (1100/an) dans des situations médicales comparables à celles des participants à cette étude.

PROTECTION DES PERSONNES

Le protocole a été examiné et approuvé par le Comité de Protection des Personnes Sud-Est V de Grenoble qui a donné son avis favorable le :…………
Le CHU de Grenoble a pris toutes les dispositions prévues par la loi sur la protection des personnes et a souscrit un contrat d'assurance relatif à cette étude auprès de la SHAM.

Vous avez le droit de refuser de participer à cette recherche et vous pouvez retirer votre consentement à tout moment, sans encourir aucune responsabilité ni aucun préjudice de ce fait (Art.L.1121-1 du Code de la Santé Publique). Votre accord ou votre refus de participer à cette étude n'affecteront en aucun votre suivi et vous bénéficierez de la prise en charge hospitalière habituelle si nécessaire.

Un exemplaire de cette fiche d'information vous est destiné.

Une information sur les résultats globaux de l'étude vous sera communiquée à l'issue de l'étude, soit par l'intermédiaire d'un fascicule-résumé envoyé par courrier, soit lors d'un entretien en consultation, selon vos préférences et disponibilités.

CONFIDENTIALITE

Compte tenu des nécessités de la recherche et de son analyse ultérieure, les données recueillies feront l'objet d'un traitement informatisé par le CHU de Grenoble, dans un but scientifique. La confidentialité sera garantie par le fait que seul un numéro d'anonymat figurera dans les analyses et documents informatisés et qu'aucune donnée nominative n'apparaîtra. Le fichier informatisé utilisé pour la saisie et le traitement des données fera l'objet d'une déclaration à la Commission Nationale Informatique et Libertés. Conformément à la loi informatique et liberté, vous disposerez d'un droit d'accès, de rectification et d'opposition aux données vous concernant. Vous pourrez exercer votre droit de consultation, de modification ou d'opposition des données vous concernant auprès de l'investigateur ayant procédé à votre inclusion. Le Pr. Levy au CHU de Grenoble est la personne auprès de qui vous pourrez exercer votre droit de consultation, de modification ou d'opposition des données vous concernant. Vous pouvez également avoir directement accès aux données. Vous avez la possibilité de vérifier auprès du ministre chargé de la santé de l'exactitude des données vous concernant présentes dans le fichier et de leur destruction au terme du délai d'un an prévu.

Pour tout renseignement, vous pouvez contacter :
Pr. Patrick LEVY, Laboratoire EFCR, Hôpital Michalon, CHU Grenoble
Tel : 04 76 76 89 56

12-1-2 Patients SAOS

==

NOTE D'INFORMATION AU PATIENT SAOS

==

Document constitué en application du Code de Santé Publique

Investigateur :
Pr. Patrick LEVY
Laboratoire EFCR, CHU de Grenoble
BP 217
38043 GRENOBLE cedex
Tél. : +33 0(4) 76 76 55 16

Promoteur : CHU de Grenoble

Titre identifiant la recherche :

Le cerveau à l'effort: effets de l'hypoxie chez le malade respiratoire

Le Dr. .... m'a proposé de participer à une recherche organisée par le C.H.U. de GRENOBLE portant sur une étude physiopathologique comportant une série d'évaluations neuromusculaires et de tests à l'exercice, avant et après traitement par pression positive continue (PPC).

Il m'a précisé que j'ai le droit de refuser de participer à cette recherche ou de retirer mon consentement, à tout moment sans encourir aucune responsabilité ni aucun préjudice de ce fait (article L1122-1 du code de la santé publique). Les données recueillies précédemment à cet arrêt seront utilisées sauf si je ne le souhaite pas. Je suis donc libre d'accepter ou de refuser. Cela ne changera pas ma prise en charge habituelle. J'ai bien reçu et j'ai bien compris les informations suivantes.
Aucun frais ne sera facturé du fait de la participation à cette étude.

BUT DE L'ETUDE

Il s'agit d'évaluer l'effet de l'hypoxie (manque d'oxygène dans le sang) chronique caractéristique des maladies respiratoires telles que la BPCO ou le syndrome d'apnée du sommeil, sur les perturbations cérébrales associées à la réalisation d'un exercice fatigant. L'objectif de l'étude est d'évaluer l'oxygénation, la perfusion et l'activation du cerveau, la réponse des neurones, ainsi que la commande du cerveau vers les muscles avant, pendant et après un exercice physique fatigant réalisé avant et après traitement par PPC.

METHODOLOGIE

Au cours d'une visite préalable dite de sélection, une vérification des critères d'inclusion ainsi qu'un examen clinique seront réalisés. L'investigateur vous remettra cette lettre d'information accompagnée du formulaire de consentement.

Si vous acceptez de participer, vous serez évalué en six occasions, trois avant traitement par PPC et trois après. Chaque session (hormis la session 3 qui sera sans effort) consistera en la réalisation d'un exercice standardisé de la jambe, suffisamment intense pour fatiguer votre muscle. L'exercice sera réalisé en position assise sur une chaise spéciale ou sur une bicyclette. Avant et après ces exercices fatigants seront réalisées des mesures par stimulation magnétique d'une partie de votre cerveau correspondant à la commande de votre jambe.

DEROULEMENT DE L'ETUDE

Un bilan médical préalable à l'étude sera réalisé. Les résultats de cet examen vous seront communiqués à l'issue de la visite de sélection par le médecin de votre choix. Le déroulement global de cette étude sera organisé en cinq visites à l'hôpital de 2-3h chacune, séparées d'au moins deux jours.
Le détail des examens réalisés à l'hôpital vous est présenté ci-après.

Sur un plan pratique :

Vous serez convoqué pour chaque visite au CHU.

Lors de la **première visite de sélection**, vous serez convoqué à l'UF Recherche sur l'Exercice et à l'Institut de Rééducation du CHU-Sud de Grenoble. Après vérification de la signature du consentement et de vos derniers résultats d'enregistrement polysomnographique du sommeil, un examen clinique et les mesures anthropométriques seront réalisés.

Elle comportera :

a. une **évaluation neuropsychologique**, qui correspond à une évaluation de vos capacités de mémoire, d'attention et de vitesse de traitement de l'information. La passation de ces tests sera effectuée par une neuropsychologue, la durée de l'évaluation est d'environ une heure.

b. dans un second temps, une **étude de différents paramètres caractérisant votre marche** sera effectuée. Le test se déroulera sur un tapis roulant, vous serez installé dans un harnais qui vous laissera libre de vos mouvements mais empêchera toute chute. Vous serez évalué sous deux conditions :

- La première à votre vitesse de marche naturelle,
- La seconde également à votre vitesse de marche naturelle mais vous serez alors confronté à une perturbation, définie pour notre protocole comme étant une augmentation de votre vitesse naturelle de marche de 1 km/h. Cette perturbation surviendra de manière aléatoire.

Dans les deux cas il vous sera demandé de marcher de la manière la plus naturelle possible. Dans la seconde condition nous évaluerons votre capacité d'adaptation à la survenue d'une perturbation alors que vous vous déplacerez. Quatre mesures d'une durée de trente secondes chacune seront réalisées pour chaque condition, soit huit mesures au total pour une durée de quatre minutes.
Pour compléter cette évaluation, il vous sera demandé d'effectuer de nouveau les deux mêmes séries de mesure mais en réalisant cette fois simultanément un test dit de STROOP qui

sollicitera votre attention. Ce test consiste en la lecture à voie haute de noms de couleurs écrits dans une couleur différente. Ce type d'évaluation couplant marche et tâche attentionnelle est appelé double tâche. Le test de STROOP sera diffusé sur un écran disposé devant vous, à hauteur de regard. Un recueil du nombre d'erreur sera effectué par l'examinateur. Il vous sera demandé de marcher le plus naturellement possible tout au long des évaluations. En condition de double tâche, quatre mesures de trente secondes chacune seront réalisées pour chaque condition de marche, soit huit évaluations au total pour une durée de quatre minutes.
Ainsi pour le test de marche, 16 mesures différentes seront réalisées pour une durée totale de huit minutes.

c. Pour finir, nous réaliserons une **évaluation de vos capacités posturales**. Les mesures seront réalisées à l'aide de deux sabots de mesure, un pour chaque pied. Comme pour l'évaluation à la marche, nous réaliserons deux séries de mesure correspondant à deux conditions d'évaluation :

- Pour la première, il vous sera demandé de rester en position debout, les bras le long du corps, un pied sur chaque sabot de mesure alors disposés à même le sol et distant l'un de l'autre de 270 millimètres, en essayant de rester le plus immobile possible.
- Pour la seconde, il vous sera demandé de rester dans la même position mais les sabots de mesure seront placés sur une plateforme instable, oscillant d'avant en arrière. Vous devrez alors essayer de maintenir la plateforme instable le plus possible à l'horizontale, en contrôlant dans la limite de vos possibilités les oscillations.

Quatre mesures seront réalisées pour chaque condition, le temps d'acquisition sera de trente secondes pour chaque mesure, soit huit mesures au total pour une durée de quatre minutes.
Comme pour la marche, ces mesures seront répétées en situation de double tâche, selon les mêmes modalités. Quatre mesures de trente secondes chacune seront réalisées pour chaque condition posturale, soit huit évaluations au total pour une durée totale de quatre minutes.
Ainsi pour l'évaluation de la posture, 16 mesures seront réalisées pour une durée totale de huit minutes.

d. Ensuite, vous réaliserez **un test d'effort sur bicyclette** : ce test consiste à pédaler de façon continue, alors que vous respirez librement dans un embout permettant de mesurer votre souffle. L'intensité de l'effort augmentera progressivement et des encouragements verbaux vous seront donnés jusqu'à ce que vous ne parveniez plus à pédaler. Régulièrement au cours de l'effort, il vous sera demandé d'évaluer sur une échelle de 1 à 10 l'intensité de votre essoufflement et l'intensité de votre fatigue des jambes. A la fin de ce test il sera possible de vous communiquer la puissance maximale que vous avez développée sur la bicyclette.

Suite à cette visite, **5 autres visites au total seront réalisées**, chacune espacées d'au moins deux jours. Elles se dérouleront toutes à l'UF Recherche sur l'exercice.

La première de ces cinq visites consistera à l'évaluation de votre fonction cérébrale avant pendant et après un effort de pédalage sur bicyclette réalisé à 80% de votre puissance maximale maintenu jusqu'à épuisement.
Avant et après cet effort fatigant, votre quadriceps sera artificiellement stimulé par un courant électrique qui sera appliqué juste en amont du muscle sur le nerf fémoral ou par une stimulation magnétique qui sera appliquée au niveau de votre crâne sur la zone correspondant

au muscle concerné. Ce genre de stimulation est sans douleur et induit une contraction involontaire de faible amplitude. Afin de mesurer la réaction de votre muscle, des électrodes seront placées sur la peau. Votre fréquence cardiaque, votre tension artérielle ainsi que l'oxygénation de votre cerveau et de votre cuisse seront mesurées et contrôlées en continu.

Lors de la seconde visite, nous procéderons à l'évaluation de votre fonction cérébrale avant et pendant et après une tâche d'extension du genou, votre genou étant fléchi à 90°, la contraction de votre cuisse devant tenter d'étendre votre jambe droite alors que votre cheville sera fixée à un capteur de force. L'exercice consistera en 5 secondes d'effort (à un niveau prédéterminé de 50% de votre force maximale préalablement mesurée et qui vous sera indiqué par un signal visuel) suivi de 5 secondes de relâchement, jusqu'à épuisement. Avant, pendant et après ce test fatigant seront réalisés les mêmes tests que lors de la première visite.

Lors de troisième visite, vous serez installé dans un fauteuil et vous respirerez à travers un embout buccal des mélanges gazeux avec différents contenus d'oxygène et de dioxyde de carbone. Ce test durera 1h30 environ, vous aurez simplement à rester éveillé et calme.

A l'issue de cette troisième visite, vous bénéficierez d'un **traitement par pression positive continue** à domicile d'une durée de deux mois selon la prise en charge habituelle des patients apnéiques du sommeil.

Lors de la quatrième visite (post traitement), vous réaliserez les mêmes tests que lors de la première visite.

Lors de la cinquième visite, vous réaliserez les mêmes tests que lors de la seconde visite.

Lors de la sixième visite, vous réaliserez les mêmes tests que lors de la troisième visite.

BENEFICES

Aucun bénéfice individuel direct n'est attendu. Les tests réalisés vous permettront cependant d'avoir plusieurs indices relatifs à votre tolérance à l'effort, à l'adaptation de votre cerveau et de vos muscles à l'effort.

DUREE DE L'ETUDE

Vous serez inclus dans l'étude pour une durée variable allant de 13 à 14 semaines.
Vous êtes libre de participer à une autre étude clinique durant votre inclusion dans le présent projet dans la mesure où celle-ci n'interfère pas avec cette recherche.

CONTRAINTES ET RISQUES

Les contraintes sont celles liées au fait de se rendre six fois à l'hôpital pour réaliser les différents tests.
Les effets secondaires prévisibles sont :

- Des courbatures au niveau du quadriceps du fait du pédalage, ainsi que des exercices de marche et de posture.
- Un inconfort lors de l'application de la stimulation électrique ou magnétique; l'application de la stimulation électrique est peu douloureuse, la survenue d'une contraction musculaire involontaire (lors de la stimulation) créant un léger désagrément ; l'application d'une stimulation magnétique de votre cerveau n'est pas douloureuse, mais peu éventuellement causer la survenue de céphalées.
- Une sensation de fatigue à l'issue des tests de marche et posture, légère sur le plan physique liée à la marche ou la posture, mais également intellectuelle, liée à l'évaluation neuropsychologique ainsi qu'à la condition de double tâche (marcher et analyser, se maintenir et analyser).
- Des risques de déséquilibre pouvant aller jusqu'à la chute lors des tests de posture ou lors de l'évaluation à la marche. Elles seront prévenues par une sécurité passive sur le tapis (harnais) et des barrières de sécurité.

Les risques prévisibles sont ceux liés aux tests à l'effort : ces tests sont réalisés par un médecin et un expérimentateur ou technicien expérimenté. Très rarement, les tests à l'effort sont associés à des complications cardiaques ou des baisses de tension qui pourraient être graves, et qui sont prévenues par l'application de procédures de surveillance et d'arrêt anticipé de l'exercice strictes. Aucune complication de ce type n'est survenue sur ce lieu de recherche considéré du CHU de Grenoble, ces tests étant régulièrement pratiqués (1100/an) dans des situations médicales comparables à celles des participants à cette étude.

PROTECTION DES PERSONNES

Le protocole a été examiné et approuvé par le Comité de Protection des Personnes Sud-Est V de Grenoble qui a donné son avis favorable le :…………
Le CHU de Grenoble a pris toutes les dispositions prévues par la loi sur la protection des personnes et a souscrit un contrat d'assurance relatif à cette étude auprès de la SHAM.

Vous avez le droit de refuser de participer à cette recherche et vous pouvez retirer votre consentement à tout moment, sans encourir aucune responsabilité ni aucun préjudice de ce fait (Art.L.1121-1 du Code de la Santé Publique). Votre accord ou votre refus de participer à cette étude n'affecteront en aucun votre suivi et vous bénéficierez de la prise en charge hospitalière habituelle si nécessaire.
Un exemplaire de cette fiche d'information vous est destiné.
Une information sur les résultats globaux de l'étude vous sera communiquée à l'issue de l'étude, soit par l'intermédiaire d'un fascicule-résumé envoyé par courrier, soit lors d'un entretien en consultation, selon vos préférences et disponibilités.

CONFIDENTIALITE

Compte tenu des nécessités de la recherche et de son analyse ultérieure, les données recueillies feront l'objet d'un traitement informatisé par le CHU de Grenoble, dans un but scientifique. La confidentialité sera garantie par le fait que seul un numéro d'anonymat figurera dans les analyses et documents informatisés et qu'aucune donnée nominative n'apparaîtra. Le fichier informatisé utilisé pour la saisie et le traitement des données fera l'objet d'une déclaration à la Commission Nationale Informatique et Libertés. Conformément

à la loi informatique et liberté, vous disposerez d'un droit d'accès, de rectification et d'opposition aux données vous concernant. Vous pourrez exercer votre droit de consultation, de modification ou d'opposition des données vous concernant auprès de l'investigateur ayant procédé à votre inclusion. Le Pr. Levy au CHU de Grenoble est la personne auprès de qui vous pourrez exercer votre droit de consultation, de modification ou d'opposition des données vous concernant. Vous pouvez également avoir directement accès aux données. Vous avez la possibilité de vérifier auprès du ministre chargé de la santé de l'exactitude des données vous concernant présentes dans le fichier et de leur destruction au terme du délai d'un an prévu.

Pour tout renseignement, vous pouvez contacter :
Pr. Patrick LEVY, Laboratoire EFCR, Hôpital Michalon, CHU Grenoble
Tel : 04 76 76 89 56

12-1-3 Sujets contrôles

===

NOTE D'INFORMATION AU TEMOIN

===

Document constitué en application du Code de Santé Publique

Investigateur :
Pr. Patrick LEVY
Laboratoire EFCR, CHU de Grenoble
BP 217
38043 GRENOBLE cedex
Tél. : +33 0(4) 76 76 55 16

Promoteur : CHU de Grenoble

Titre identifiant la recherche :

Le cerveau à l'effort: effets de l'hypoxie chez le malade respiratoire

Le Dr. .... m'a proposé de participer à une recherche organisée par le C.H.U. de GRENOBLE portant sur une étude physiopathologique comportant une série d'évaluations neuromusculaires et de tests à l'exercice

Il m'a précisé que j'ai le droit de refuser de participer à cette recherche ou de retirer mon consentement, à tout moment sans encourir aucune responsabilité ni aucun préjudice de ce fait (article L1122-1 du code de la santé publique). Les données recueillies précédemment à cet arrêt seront utilisées sauf si je ne le souhaite pas. Je suis donc libre d'accepter ou de refuser. Cela ne changera pas ma prise en charge habituelle. J'ai bien reçu et j'ai bien compris les informations suivantes.
Aucun frais ne sera facturé du fait de la participation à cette étude.

BUT DE L'ETUDE

Il s'agit d'évaluer l'effet de l'hypoxie (manque d'oxygène dans le sang) chronique caractéristique des maladies respiratoires telles que la BPCO ou le syndrome d'apnée du sommeil, sur les perturbations cérébrales associées à la réalisation d'un exercice fatigant. L'objectif de l'étude est d'évaluer l'oxygénation, la perfusion et l'activation du cerveau, la réponse des neurones, ainsi que la commande du cerveau vers les muscles avant, pendant et après un exercice physique fatigant réalisé en respirant l'air ambiant ou un mélange gazeux enrichi en oxygène.

METHODOLOGIE

Au cours d'une visite préalable dite de sélection, une vérification des critères d'inclusion ainsi qu'un examen clinique seront réalisés. L'investigateur vous remettra cette lettre d'information accompagnée du formulaire de consentement.

Si vous acceptez de participer, vous serez évalué en trois occasions. Chaque session (hormis la session 3 qui sera sans effort) consistera en la réalisation d'un exercice standardisé de la jambe, suffisamment intense pour fatiguer votre muscle. L'exercice sera réalisé en position assise sur une chaise spéciale ou sur une bicyclette. Avant et après ces exercices fatigants seront réalisées des mesures par stimulation magnétique d'une partie de votre cerveau correspondant à la commande de votre jambe.

DEROULEMENT DE L'ETUDE

Un bilan médical préalable à l'étude sera réalisé. Les résultats de cet examen vous seront communiqués à l'issue de la visite de sélection par le médecin de votre choix. Le déroulement global de cette étude sera organisé en trois visites à l'hôpital de 2-3 h chacune, séparées d'au moins deux jours.

Le détail des examens réalisés à l'hôpital vous est présenté ci-après :

Sur un plan pratique :

Vous serez convoqué pour chaque visite au CHU.

Lors de la **première visite de sélection**, vous serez convoqué à l'UF Recherche sur l'Exercice du CHU de Grenoble et à l'Institut de Rééducation du CHU-Sud de Grenoble. Après vérification de la signature du consentement, un examen clinique et les mesures anthropométriques (taille, poids, etc) seront réalisés.

Elle comportera :

a. une **évaluation neuropsychologique**, qui correspond à une évaluation de vos capacités de mémoire, d'attention et de vitesse de traitement de l'information. La passation de ces tests sera effectuée par une neuropsychologue, la durée de l'évaluation est d'environ une heure.

b. dans un second temps, une **étude de différents paramètres caractérisant votre marche** sera effectuée. Le test se déroulera sur un tapis roulant, vous serez installé dans un harnais qui vous laissera libre de vos mouvements mais empêchera toute chute. Vous serez évalué sous deux conditions :
   - La première à votre vitesse de marche naturelle,
   - La seconde également à votre vitesse de marche naturelle mais vous serez alors confronté à une perturbation, définie pour notre protocole comme étant une augmentation de votre vitesse naturelle de marche de 1 km/h. Cette perturbation surviendra de manière aléatoire.

Dans les deux cas il vous sera demandé de marcher de la manière la plus naturelle possible. Dans la seconde condition nous évaluerons votre capacité d'adaptation à la survenue d'une perturbation alors que vous vous déplacerez. Quatre mesures d'une durée de trente secondes chacune seront réalisées pour chaque condition, soit huit mesures au total pour une durée de quatre minutes.

Pour compléter cette évaluation, il vous sera demandé d'effectuer de nouveau les deux mêmes séries de mesure mais en réalisant cette fois simultanément un test dit de STROOP qui

sollicitera votre attention. Ce test consiste en la lecture à voie haute de noms de couleurs écrits dans une couleur différente. Ce type d'évaluation couplant marche et tâche attentionnelle est appelé double tâche. Le test de STROOP sera diffusé sur un écran disposé devant vous, à hauteur de regard. Un recueil du nombre d'erreur sera effectué par l'examinateur. Il vous sera demandé de marcher le plus naturellement possible tout au long des évaluations. En condition de double tâche, quatre mesures de trente secondes chacune seront réalisées pour chaque condition de marche, soit huit évaluations au total pour une durée de quatre minutes.
Ainsi pour le test de marche, 16 mesures différentes seront réalisées pour une durée totale de huit minutes.

c. Pour finir, nous réaliserons une **évaluation de vos capacités posturales**. Les mesures seront réalisées à l'aide de deux sabots de mesure, un pour chaque pied. Comme pour l'évaluation à la marche, nous réaliserons deux séries de mesure correspondant à deux conditions d'évaluation :

- Pour la première, il vous sera demandé de rester en position debout, les bras le long du corps, un pied sur chaque sabot de mesure alors disposés à même le sol et distant l'un de l'autre de 270 millimètres, en essayant de rester le plus immobile possible.
- Pour la seconde, il vous sera demandé de rester dans la même position mais les sabots de mesure seront placés sur une plateforme instable, oscillant d'avant en arrière. Vous devrez alors essayer de maintenir la plateforme instable le plus possible à l'horizontale, en contrôlant dans la limite de vos possibilités les oscillations.

Quatre mesures seront réalisées pour chaque condition, le temps d'acquisition sera de trente secondes pour chaque mesure, soit huit mesures au total pour une durée de quatre minutes.
Comme pour la marche, ces mesures seront répétées en situation de double tâche, selon les mêmes modalités. Quatre mesures de trente secondes chacune seront réalisées pour chaque condition posturale, soit huit évaluations au total pour une durée totale de quatre minutes.
Ainsi pour l'évaluation de la posture, 16 mesures seront réalisées pour une durée totale de huit minutes.

d. Ensuite, vous réaliserez **un test d'effort sur bicyclette :** ce test consiste à pédaler de façon continue alors que vous respirez librement dans un embout permettant de mesurer votre souffle. L'intensité de l'effort augmentera progressivement et des encouragements verbaux vous seront donnés jusqu'à ce que vous ne parveniez plus à pédaler. Régulièrement au cours de l'effort, il vous sera demandé d'évaluer sur une échelle de 1 à 10 l'intensité de votre essoufflement et l'intensité de votre fatigue des jambes. A la fin de ce test il sera possible de vous communiquer la puissance maximale que vous avez développée sur la bicyclette.

Suite à cette visite, **3 autres visites au total seront réalisées**, chacune espacées d'au moins deux jours. Elles se dérouleront toutes à l'UF Recherche sur l'exercice.

La première de ces trois visites consistera à l'évaluation de votre fonction cérébrale avant pendant et après un effort sur bicyclette réalisé à 80% de votre puissance maximale maintenu jusqu'à épuisement.
Avant et après cet effort fatigant, votre quadriceps sera artificiellement stimulé par un courant électrique qui sera appliqué juste en amont du muscle sur le nerf fémoral ou par une stimulation magnétique qui sera appliquée au niveau de votre crâne sur la zone de votre

cerveau correspondant au muscle concerné. Ce genre de stimulation est sans douleur et induit une contraction involontaire de faible amplitude. Afin de mesurer la réaction de votre muscle, des électrodes seront placées sur la peau. Votre fréquence cardiaque, votre tension artérielle ainsi que l'oxygénation de votre cerveau et de votre cuisse seront mesurées et contrôlées en continu.

Lors de la seconde visite, vous réaliserez les mêmes tests que lors de la visite de sélection. Nous procéderons par la suite à l'évaluation de votre fonction cérébrale avant, pendant et après un exercice d'extensions du genou, votre genou étant fléchi à 90°, la contraction de votre cuisse devant tenter d'étendre votre jambe droite alors que votre cheville sera fixée à un capteur de force. L'exercice consistera en 5 secondes d'effort (à un niveau prédéterminé de 50% de votre force maximale préalablement mesurée et qui vous sera indiqué par un signal visuel) suivi de 5 secondes de relâchement, jusqu'à épuisement. Avant, pendant et après ce test fatigant seront réalisés les mêmes tests que lors de la première visite.

Lors de troisième visite, vous serez installé dans un fauteuil et vous respirerez à travers un embout buccal des mélanges gazeux avec différents contenus d'oxygène et de dioxyde de carbone. Ce test durera 1h30 environ, vous aurez simplement à rester éveillé et calme.

BENEFICES

Aucun bénéfice individuel direct n'est attendu. Les tests réalisés vous permettront cependant d'avoir plusieurs indices relatifs à votre tolérance à l'effort, à l'adaptation de votre cerveau et de vos muscles à l'effort.

DUREE DE L'ETUDE

Vous serez inclus dans l'étude pour une durée variable allant de 3 à 4 semaines.

Vous êtes libre de participer à une autre étude clinique durant votre inclusion dans le présent projet dans la mesure où celle-ci n'interfère pas avec cette recherche.

CONTRAINTES ET RISQUES

Les contraintes sont celles liées au fait de se rendre six fois à l'hôpital pour réaliser les différents tests.

Les effets secondaires prévisibles sont :

- Des courbatures au niveau du quadriceps du fait du pédalage, ainsi que des exercices de marche et de posture.
- Un inconfort lors de l'application de la stimulation électrique ou magnétique; l'application de la stimulation électrique est peu douloureuse, la survenue d'une contraction musculaire involontaire (lors de la stimulation) créant un léger désagrément ; l'application d'une stimulation magnétique de votre cerveau n'est pas douloureuse, mais peu éventuellement causer la survenue de céphalées.

- Une sensation de fatigue à l'issue des tests de marche et posture, légère sur le plan physique liée à la marche ou la posture, mais également intellectuelle, liée à l'évaluation neuropsychologique ainsi qu'à la condition de double tâche (marcher et analyser, se maintenir et analyser).
- Des risques de déséquilibre pouvant aller jusqu'à la chute lors des tests de posture ou lors de l'évaluation à la marche. Elles seront prévenues par une sécurité passive sur le tapis (harnais) et des barrières de sécurité.

Les risques prévisibles sont ceux liés aux tests à l'effort : ces tests sont réalisés par un médecin et un expérimentateur ou technicien expérimenté. Très rarement, les tests à l'effort sont associés à des complications cardiaques ou des baisses de tension qui pourraient être graves, et qui sont prévenues par l'application de procédures de surveillance et d'arrêt anticipé de l'exercice strictes. Aucune complication de ce type n'est survenue sur ce lieu de recherche considéré du CHU de Grenoble, ces tests étant régulièrement pratiqués (1100/an) dans des situations médicales comparables à celles des participants à cette étude.

PROTECTION DES PERSONNES

Le protocole a été examiné et approuvé par le Comité de Protection des Personnes Sud-Est V de Grenoble qui a donné son avis favorable le :…………
Le CHU de Grenoble a pris toutes les dispositions prévues par la loi sur la protection des personnes et a souscrit un contrat d'assurance relatif à cette étude auprès de la SHAM.

Vous avez le droit de refuser de participer à cette recherche et vous pouvez retirer votre consentement à tout moment, sans encourir aucune responsabilité ni aucun préjudice de ce fait (Art.L.1121-1 du Code de la Santé Publique). Votre accord ou votre refus de participer à cette étude n'affecteront en aucun votre suivi et vous bénéficierez de la prise en charge hospitalière habituelle si nécessaire.

Un exemplaire de cette fiche d'information vous est destiné.

Une information sur les résultats globaux de l'étude vous sera communiquée à l'issue de l'étude, soit par l'intermédiaire d'un fascicule-résumé envoyé par courrier, soit lors d'un entretien en consultation, selon vos préférences et disponibilités.

CONFIDENTIALITE

Compte tenu des nécessités de la recherche et de son analyse ultérieure, les données recueillies feront l'objet d'un traitement informatisé par le CHU de Grenoble, dans un but scientifique. La confidentialité sera garantie par le fait que seul un numéro d'anonymat figurera dans les analyses et documents informatisés et qu'aucune donnée nominative n'apparaîtra. Le fichier informatisé utilisé pour la saisie et le traitement des données fera l'objet d'une déclaration à la Commission Nationale Informatique et Libertés. Conformément à la loi informatique et liberté, vous disposerez d'un droit d'accès, de rectification et d'opposition aux données vous concernant. Vous pourrez exercer votre droit de consultation, de modification ou d'opposition des données vous concernant auprès de l'investigateur ayant procédé à votre inclusion. Le Pr. Levy au CHU de Grenoble est la personne auprès de qui

vous pourrez exercer votre droit de consultation, de modification ou d'opposition des données vous concernant. Vous pouvez également avoir directement accès aux données. De plus, vous serez inscrit sur le fichier national des personnes se prêtant à des recherches biomédicales. Vous avez la possibilité de vérifier auprès du ministre chargé de la santé de l'exactitude des données vous concernant présentes dans le fichier et de leur destruction au terme du délai d'un an prévu.

Pour tout renseignement, vous pouvez contacter :

Pr. Patrick LEVY, Laboratoire EFCR, Hôpital Michalon, CHU Grenoble
Tel : 04 76 76 89 56

12-2 CONSENTEMENT PATIENT / TEMOIN

==

FORMULAIRE DE CONSENTEMENT

==

Investigateurs :
Pr. Patrick LEVY
Laboratoire EFCR, CHU de Grenoble
BP 217
38043 GRENOBLE cedex
Tél. : +33 0(4) 76 76 55 16

Promoteur : CHU de Grenoble

Titre identifiant la recherche :

Le cerveau à l'effort: effets de l'hypoxie chez le malade respiratoire

NOM : ....

Prénom :...

Je reconnais avoir pris connaissance de la façon dont se déroule l'étude.

L'intérêt, la durée et les modalités de cette étude m'ont été présentés par le docteur ……………………….. qui m'a remis une note d'information.

Il m'a précisé que je suis libre d'accepter ou de refuser ; cela ne changera pas le traitement ou les soins qui me seront appliqués.
Il m'a précisé que je serai libre à tout moment d'arrêter ma participation sans encourir de responsabilités.

J'ACCEPTE DE PARTICIPER A CETTE ETUDE DANS LES CONDITIONS PRECISEES CI-DESSUS

Si je le désire, je serai libre à tout moment d'arrêter ma participation sans encourir aucune responsabilité, ni aucun préjudice de ce fait. J'en informerai alors le Pr. LEVY.
Les données qui me concernent resteront strictement confidentielles. Je n'autorise leur consultation que par des personnes soumises au secret professionnel et collaborant à cette recherche.
Je pourrai à tout moment demander toute information complémentaire auprès des médecins investigateurs.

J'accepte que les données enregistrées à l'occasion de cette étude puissent faire l'objet d'un traitement informatisé, après l'anonymat, par le promoteur ou pour son compte. J'ai bien noté que mon droit d'accès prévu par la loi informatique et liberté s'exerce à tout moment.
J'ai reçu une fiche d'information détaillée. J'ai reçu copie du présent document, j'ai été informé(e) qu'une copie sera également conservée par les organisateurs dans des conditions garantissant la confidentialité, et y consens. J'ai été informé que conformément à la réglementation sur les études cliniques, le CPP de Grenoble a rendu un AVIS FAVORABLE pour la réalisation de cette recherche, en date du …./…./……..L'Agence Française de Sécurité Sanitaire des Produits de Santé (AFSSAPS) a donné son autorisation.

J'ai pris bonne note que les données que je fournirai seront traitées de façon strictement anonyme et confidentielle. Je n'autorise leur consultation que par des personnes qui collaborent à la recherche, désignées par l'organisateur.

Je pourrai à tout moment demander toute information complémentaire au
Pr. Patrick LEVY
Tel : 04 76 76 89 56
E-mail : plevy@chu-grenoble.fr

<u>Personne se prêtant à la recherche</u>	<u>Médecin investigateur</u>
Date : _____________	Date : _____________
Nom et Prénom : _____________________	Nom et Prénom : ____________________
Signature :	Signature :

12-3 CV DES INVESTIGATEURS

Tableau d'identification des investigateurs

Prénom, Nom	Adresse professionnelle	Numéro d'inscription au Conseil de l'Ordre des Médecins
Pr. Patrick LEVY	Laboratoire d'Explorations Fonctionnelles Cardio-Respiratoires et Laboratoire du Sommeil, Hôpital Michalon, 38043 Grenoble cedex	38/03573
Dr. Bernard WUYAM	UF Recherche sur l'exercice, Hôpital Sud, avenue Kimberley, 38400 Echirolles	38/ 5162
Pr Jean-Louis PEPIN	Pôle de Rééducation et Physiologie, Laboratoire d'EFCR et Laboratoire du Sommeil, CHU Michallon, BP 217, 38043 Grenoble Cedex 9	38/ 05405
Dr. Renaud TAMISIER	Pôle de Rééducation et Physiologie, Laboratoire d'EFCR et Laboratoire du Sommeil, CHU Michallon, BP 217, 38043 Grenoble Cedex 9	38/ 08265
Pr. Dominique PERENNOU	Clinique de Médecine Physique et Réadaptation<br>Laboratoire TimC-IMAG UMR UJF CNRS 5525<br>Equipe Motricité – Plasticité<br>Institut de Rééducation – Hôpital sud CHU<br>BP 338, 38434 Echirolles cedex	RPPS 10002184769

Patrick Lévy

**Thématiques de recherche**	Physiopathologie et prise en charge clinique des troubles respiratoires nocturnes

Diplômes

N° Conseil de l'Ordre : 38/03573
N° ADELI : 38 10 357 32

- Doctorat en Médecine
- Certificat d'Etudes Spéciales de Pneumo-phtisiologie
- Diplôme d'Etudes Approfondies de Biologie et Physiologie animale, Université Louis Pasteur, Strasbourg
- Doctorat de Biologie, Université Joseph Fourier, Grenoble
- Diplôme d'Habilitation à Diriger la Recherche : "Hypoxie chronique et Sommeil"

Titres

- Maître de Conférences des Universités - Praticien Hospitalier, (Physiologie, option biologique) 1/10/1989 au 31/08/1997
- Membre du PRETA (TimC UMR CNRS 5525) depuis 1989
- Directeur du PRETA (TimC UMR CNRS 5525) depuis 1998
- Responsable scientifique et médical du Secteur Sommeil et Respiration, CHU de Grenoble, depuis novembre 1990
- Professeur des Universités depuis le 1/09/97, 1ère classe en 09/02
- Chef de service du Laboratoire d'EFCR, CHU de Grenoble, depuis le 1/06/01
- Président de la Délégation Régionale à la Recherche Clinique depuis 2003
- Coordinateur du groupe Recherche de l'UFR Médecine UJF depuis 1999
- Membre du Conseil d'Administration de l'Université Joseph Fourier depuis 1999
- Membre de la Commission de la Recherche des Sciences de la Vie et de la Santé, Université Joseph Fourier, 2000
- Directeur du Laboratoire HP2 depuis janvier 2002, Faculté de Médecine de Grenoble
- Médecin responsable du Pôle Rééducation-Physiologie du CHU de Grenoble, depuis le 1/01/2007

Principales publications

J. ARGOD, J.L. PEPIN, R. SMITH, **P. LEVY.** Comparison of esophageal pressure (Pes) and Pulse transit time (PTT) as a measure of respiratory effort for scoring obstructive non-apneic respiratory events (ONAREs). *Am J Respir Crit Care Med,* 2000;162:87-93.

R. TAMISIER, J.L. PEPIN, B. WUYAM, R. SMITH, J. ARGOD, **P. LEVY.**

Characterization of Pharyngeal Resistance during Sleep in a spectrum of Sleep-Disordered Breathing. *J Appl Physiol*, 2000;89:120-30.

C CRACOWSKI, JL PEPIN, B WUYAM, **P LEVY**. **C**haracterization of obstructive non-apneic respiratory events (ONAREs) in moderate sleep apnea syndrome. *Am J Respir Crit Care Med,* 2001;164:944-948

P LEVY, JL PEPIN. Autoadjusting continuous positive airway pressure. What can we expect? (editorial) *Am J Respir Crit Care Med*, 2001;16:1295-6

M DEMATTEIS, JL PEPIN, M JEANMART, C DESCHAUX, A LABARRE-VILA, **P LEVY**. Systematic association between Charcot-Marie-Tooth disease (CMT1A) and Sleep Apnoea Syndrome: a family study. *Lancet*, 2001;357:267–72

F.X. PETIT, J.L. PÉPIN, G. BETTEGA, H. SADEK, B. RAPHAEL, **P. LEVY**. Mandibular advancement devices (MAD): Contraindications and rate of primary indications in 100 consecutive OSA patients. *Am J Respir Crit Care Med,* 2002;166:274-278

S MAZZA, PEPIN, J.L., DESCHAUX, C., NAEGELE, B, **LEVY, P**. Analysis of errors occuring during the Osler TEST: A sensitive mean of detecting fluctuations in vigilance in OSAS patients. *Am J Respir Crit Care Med*, , 2002;166:474-478

Baguet J-Ph, Hammer L., **Lévy P**., Pierre H., Launois S., Mouret S., Mallion J-M, Pépin JL. The severity of oxygen desaturation is prdictive of carotid wall thickening and plaques occurrence. Chest 2005;128:3407-3412

Mazza S., Pépin JL, Naëgelé B., Rauch E., Deschaux C., Ficheux P, **Lévy**

P. Sleep apnoea patients driving ability before and after CPAP treatment. Evaluation on a road safety platform. Eur Respir J 2006;28:1020-8

GARRIGUE S., PÉPIN JL., DEFAYE P., MURGATROYD F., POEZEVARA Y. CLEMENTY J., **LÉVY P.** High Prevalence of Sleep Apnea Syndrome in Chronically Paced Patients: The European Multicenter Polysomnographic Study. Circulation 2007; 115:1703-09

G. DEVOUASSOUX**, P. LÉVY**, E ROSSINI, I PIN, M FIOR-GOZLAN MD, M. HENRY, D SEIGNEURIN, JL- PÉPIN**.** Sleep apnea is associated with bronchial inflammation and continuous positive airway pressure-induced. airway hyperresponsiveness. J Allergy Clin Immun 2007;119:597-603

Dr Bernard WUYAM

Thématiques de recherche

Recherche sur l'exercice musculaire et l'hypoxie

Diplômes

N° Conseil de l'Ordre : 38 / 5162; N° ADELI : 38 10 5162 2

- **Docteur en Médecine**. Faculté de Médecine de Grenoble (1988).
- **DEA de Physiologie et Physiopathologie Respiratoire**. Université René Descartes. Paris (1988).
- **Thèse d'Université** de l'Université Joseph Fourier -Grenoble I, spécialité : Biologie, option Physiologie. (1992)Faculté de Médecine. UJF.
- **Habilitation à Diriger les Recherches** [Université Joseph Fourier] **2005**

Titres & fonctions

- **Ancien Assistant des hôpitaux de Grenoble : 1988-91**
- **Ancien Interne des Hôpitaux de Grenoble : 1982 –88**
- **Ancien-Chef de clinique** à la Faculté de Médecine de Grenoble (1988-91).
- **Praticien Hospitalier 1993 - :**
  Laboratoire d'Exploration Fonctionnelle Cardio-Respiratoire. CHU de Grenoble. Laboratoire Physiopathologie Respiratoire. Hôpital A. Michallon. **Responsable de l'UF '*Recherche clinique sur l'exercice - SUD'*** depuis le **1° Janvier 2004**.
- Responsable Laboratoire Recherche Exercice Santé **REx-S, Equipe émergente, IFR-1_CHU-Sud. UJF**, depuis le 1/1/2007. Equipe **Exercice Hypoxie HP2. INSERM ERI 017**.

Formation

- Médicale à Lyon
- Interne et Assistant Chef de clinique : CHU de Grenoble. Thèse Médecine Grenoble 1988.
- Thèse d'Université Joseph Fourier en Biologie, option Physiologie (1992).
- Séjour post-doctoral (1992-93): Charing-Cross & Westminster Medical School, **Londres** (1992-93). Pr Abraham GUZ**.** Lauréat d'une **Bourse de formation INSERM** & **d'un Wellcome Trust International Fellowship**.
- **Professeur Invité AP-HP Pitié Salpetrière**, Laboratoire de Physiopathologie Respiratoire Pr Similowski en 2004 .

Principales publications

(5 dernières années)

Flore P, Tonini J, Pepin JL, Levy P, **Wuyam B**. Obstructive sleep apnoea:hypoapnoea syndrome reversibly depresses cardiac response to exercise. ***Eur Heart J***. 2006 Jul;27(13):1632-3. Epub 2006 May 25.

Vivodtzev I, Pépin JL, Vottero G, Mayer V, Porsin B, Lévy P, **Wuyam B.** Improvement in quadriceps strength and dyspnea in daily tasks after 1 month of electrical stimulation in severely deconditioned and malnourished COPD. ***Chest***. 2006 Jun;129(6):1540

Verges S, Tonini J, **Flore P**, Favre-Juvin A, Lévy P, **Wuyam B**. Exhaled nitric oxide in single and repetitive prolonged exercise. ***J Sports Sci***. 2006 Nov;24(11):1157

Chouri-Pontarollo N, Borel JC, Tamisier R, **Wuyam B**, Levy P, Pépin JL.

Impaired objective daytime vigilance in obesity-hypoventilation syndrome: impact of noninvasive ventilation. ***Chest***. 2007 Jan;131(1):148-55.

Vivodtzev I, **Flore P**, Levy P & **Wuyam B**. *IFR-1_CHU-Sud, REX-S* Voluntary activation during knee extensions in severely deconditioned patients with chronic obstructive pulmonary disease: Benefit of endurance training. ***Muscle Nerve***. 2007 Oct 2; [Epub ahead of print] .

Michallet AS, Tonini J, Regnier J, Guinot M, Favre-Juvin A, Bricout V, Halimi S, Wuyam B, Flore P. ***Diabetes Metab***. 2008 Nov;34(5):514-23. Epub 2008 Sep 26.

Michallet AS, Tonini J, Regnier J, Guinot M, Favre-Juvin A, Bricout V, Halimi S, Wuyam B, Flore P. Methodological aspects of crossover and maximum fat-oxidation rate point determination**Diabetes Metab**. 2008 Nov;34(5):514-23. Epub 2008 Sep 26.

Decorte N, Verges S, Flore P, Guinot M, Wuyam B. Effects of acute salbutamol inhalation on quadriceps force and fatigability. **Med Sci Sports Exerc**. 2008 Jul;40(7):1220-7.

Vaillant J, Meunier D, Caillat-Miousse JL, Virone G, Wuyam B, Juvin R. Impact of nociceptive stimuli on cervical kinesthesia **Ann Readapt Med Phys**. 2008 May;51(4):257-62. Epub 2008 Apr 29.

Borel JC, Wuyam B, Chouri-Pontarollo N, Deschaux C, Levy P, Pépin JL. During exercise non-invasive ventilation in chronic restrictive respiratory failure. **Respir Med**. 2008 May;102(5):711-9. Epub 2008 Feb 15.

Flore P, Bricout VA, van Biesen D, Guinot M, Laporte F, Pépin JL, Eberhard Y, Favre-Juvin A, Wuyam B, van de Vliet P, Faure P. Oxidative stress and metabolism at rest and during exercise in persons with Down syndrome. **Eur J Cardiovasc Prev Rehabil**. 2008 Feb;15(1):35-42.

Verges S, Maffiuletti NA, Kerherve H, Decorte N, Wuyam B, Millet GY. Comparison of electrical and magnetic stimulations to assess quadriceps muscle function. **J Appl Physiol**. 2009 Feb;106(2):701-10. Epub 2008 Aug 28.

Laza F, Tamisier R, Wuyam B, Borel JC, Saint Raymond C, Labarre-Vila A, Lévy P, Pépin JL. Chronic respiratory failure and Steinert's myotonia. **Rev Mal Respir**. 2009 Jan;26(1):83-6.

Vivodtzev I, Minet C, Wuyam B, Borel JC, Vottero G, Monneret D, Baguet JP, Lévy P, Pépin JL. Significant improvement in arterial stiffness after endurance training in COPD patients.
Chest. 2009 Oct 31. [Epub ahead of print]

Borel JC, Verges S, Pepin JL, Vivodtzev I, Levy P, Wuyam B. Home exercise

training with non-invasive ventilation in thoracic restrictive respiratory disorders: a randomised study.
Respir Physiol Neurobiol. 2009 Jun 30;167(2):168-73. Epub 2009 Apr 8.

Vergès S, Flore P, Nantermoz G, Lafaix PA, Wuyam B. Respiratory muscle training in athletes with spinal cord injury. **Int J Sports Med**. 2009 Jul;30(7):526-32. Epub 2009 Mar 19.

CURRICULUM VITAE ABREGE

Jean-Louis PEPIN

Date de naissance : 24 septembre 1959 (Le Puy, France). Marié, trois enfants
N° Conseil de l'Ordre : 38/05405
N° ADELI : 381054055

- Diplômes (intitulés et dates d'obtention)

- Certificat d'Etudes Spéciales en Pneumologie (Montpellier), **1987**
- Doctorat en Médecine (Montpellier), **1989**
- Diplôme Inter Universitaire Veille-Sommeil, Montpellier (Major de promotion), **1990**
- D.E.A. de Neurosciences, Université Claude Bernard (Lyon), **1993**
- Doctorat en sciences de l'Université Joseph Fourier (Diagnostic du Syndrome d'Apnées du Sommeil), Grenoble, **2001**
- Habilitation à Diriger des Recherches (Diagnostic et traitement du Syndrome d'Apnées du Sommeil), Grenoble Décembre **2002**

- Fonctions hospitalières

- Depuis 1990 : Activité clinique et de recherche clinique au sein du service de Pneumologie et du Laboratoire du Sommeil (CHU Michallon, Grenoble)
- 1995 : Praticien Hospitalier, Service de Pneumologie, CHU Michallon, Grenoble
- 1995-2002 : Membre du Laboratoire PRETA TIMC UMR CNRS 5525 (Physiologie Respiratoire Expérimentale, Théorique et Appliquée). Université Joseph Fourier, Faculté de Médecine-pharmacie de Grenoble
- 2000 : Médecin responsable de l'unité Insuffisance respiratoire et troubles respiratoires au cours du sommeil (24 lits) CHU Grenoble
- 1996 : Directeur médical et Vice-président (2000) de l'Association Régionale des Insuffisants Respiratoires chroniques (AGIR À DOM) Grenoble (9000 patients suivis à domicile)
- 2002 : Membre du Laboratoire HP2 : Hypoxie : PhysioPathologies : INSERM ERI 017. Actuellement, Responsable de l'équipe « recherche clinique ».
- 2004 : Professeur des Universités (Physiologie Clinique) depuis le 1/09/2004
- 2007 : Médecin responsable de la Clinique de Physiologie, Sommeil et Exercice du Pôle Hospitalier Rééducation – Physiologie du CHU de Grenoble
- 2007 : Expert consultant du Laboratoire du sommeil de Genève

- Formations de recherche fréquentées en France et/ou à l'étranger (dates, lieu et responsable)

"Visiting professor": Laboratoire de Physiologie respiratoire - Université d'Harvard, Dr J.W. WEISS, BOSTON, USA, Sept-Oct 1999

- Dernières publications

Pépin JL , Chouri-Pontarollo N., Tamisier R., Lévy P. Cheyne-Stokes respiration (CSR) with central sleep apnoea (CSA) [CSR-CSA] in chronic heart failure: Proposals for a diagnostic and therapeutic strategy. Sleep Med Rev 2006;10:33-47

K Palombi, E Renard, P Levy, C Chiquet, Ch Deschaux, JP Romanet, JL Pépin. Nonarteritic Anterior Ischemic Optic Neuropathy is nearly systematically associated with obstructive sleep apnea. Br J Ophthalmol 2006;90: 879-82

Mazza S., Pépin JL, Naëgelé B., Rauch E., Deschaux C., Ficheux P, Lévy P. Sleep apnoea patients driving ability before and after CPAP treatment. Evaluation on a road safety platform. Eur Respir J 2006;28:1020-8

Vivodtzev I, Pépin JL, Vottero G, Mayer V, Porsin B, Lévy P, Wuyam B. Improvement in quadriceps strength and dyspnea in daily tasks after 1 month of electrical stimulation in severely deconditioned and malnourished COPD. Chest. 2006;129:1540-8

Flore P, Tonini J, Pépin JL, Lévy P, Wuyam B. Obstructive sleep apnoea: hypopnoea syndrome reversibly depresses cardiac response to exercise. Eur Heart J. 2006;27:16323

Naegele B, Launois SH, Mazza S, Feuerstein C, Pépin JL, Levy P. Which memory processes are affected in patients with obstructive sleep apnea? An evaluation of 3 types of memory. Sleep. 2006;29:533-44.

G. Devouassoux, P. Lévy, E Rossini, I Pin, M Fior-Gozlan MD, M. Henry, D Seigneurin, JL-Pépin. Sleep apnea is associated with bronchial inflammation and continuous positive airway pressure-induced airway hyperresponsiveness. J Allergy Clin Immun 2007;119:597-603

N Chouri-Pontarollo, JC Borel, R Tamisier, B Wuyam, P Levy, JL Pépin. Impaired objective daytime vigilance in obesity hypoventilation syndrome: Impact of non-invasive ventilation. Chest 2007;131:148-155

Launois S., Pépin JL, Levy P. Sleep apnea in the elderly: A specific entity ? Sleep Medicine Reviews 2007;11:87-97

Garrigue S., Pépin JL., Defaye P., Murgatroyd F., Poezevara Y. Clementy J., Lévy P. High Prevalence of Sleep Apnea Syndrome in Chronically Paced Patients: The European Multicenter Polysomnographic Study. Circulation 2007 ; 115 :1703-09

TAMISIER Renaud

Laboratoire d'Exploration Fonctionnelle Respiratoire
Centre Hospitalier Universitaire de Grenoble BP 217
38043 Grenoble Cedex 9
Tel : +33(0)476767575 post 64206
Fax : +33(0)476765617
E-mail: rtamisier@chu-grenoble.fr
N° inscription à l'ordre des médecins : **38 08265 ;** n° ADELI **: 381082650**

Etudes:
- Thèse de médecine, Faculté de Médecine, Nice 9 juin 1998

Diplômes:
- DEA en physiologie respiratoire: Mesure des résistances pharyngées au cours du sommeil. Creteil 1997.
- Diplôme d'Etude Spécialisée en PNEUMOLOGIE, Faculté de Médecine, Nice 31 octobre 1998.
- Diplôme Inter-Universitaire de Physiologie et Pathologie du sommeil, Faculté de médecine, Grenoble Juin 2000.
- Thèse de science en Modélisation et Instrumentation en Médecine et Biologie. Physiologie des voies aériennes supérieures au cours du sommeil. Université J Fourier, Grenoble, 1998-2001.

Responsabilités Hospitalières:
1998-2000 : Assistant chef de clinique en Pneumologie, CHU Nice, responsabilité de 15 lits service de Pneumologie traditionnelle (Pr B. Blaive)
2000-2002 : Assistant Hospitalo-Universitaire en Physiologie CHU Nice, responsable Médical de l'unité d'exploration fonctionnelles respiratoire.
1998-2002 : Responsable médical du service d'exploration des pathologies respiratoires au cours du sommeil, CHU Nice
1998-2001 : Participe au tableau de garde de réanimation médicale (50 gardes/an) CHU Nice (Pr M. Mattei).
Depuis Août 2004 : Praticien Hospitalier CHU de Grenoble, Responsable médical de L'UF 4ième B du Département de Médecine Aiguë Spécialisée.

Activité de recherche et universitaire:
1998-2002 : Membre de l'équipe de recherche (PRETA), département de Physiologie, Faculté de Medecine, Université Joseph Fourier, (unité CNRS 5525 TIM-C depuis 1995)
Depuis 2002 : Membre de l'équipe de recherche HP2 (Hypoxie PathoPhysiologie) département de Physiologie, Faculté de Medecine, Université Joseph Fourier, Grenoble France
Depuis 2002 : Membre de l'équipe de recherche du Dr J W Weiss au Pulmonary Research Laboratory, Beth Israel Deaconess Medical Center, Harvard Medical School, Boston, Massachusetts USA.

Responsabilités extra-hospitalières:
1999- 2002 Membre et conseillé médical de l'association régionale d'assistance respiratoire à domicile (ARARD)

Programation et Réunion scientifique
2001- Membre du Comité de programmation et d'organisation du Congrès de la SFRS et groupe sommeil de la SPLF Nice 2001
Depuis 2004 - Membre du Comité de programmation et d'organisation du Sleep & Breathing Symposium, Newport RI USA, Octobre 2004
Depuis 2004 - Membre du Comité de programmation de l'assemblée Respiratory and Neurobiology of Sleep de l'American Thoracic Society
Depuis 2004 – Reviewer pour l'American Journal of Respiratory and Critical Care

Sociétés Professionnelles:
Depuis 2000 - Membre de la Société française de Recherche du Sommeil
Depuis 2001 - Membre de la Société de Pneumologie de Langue française

Dernières publications

Baguet, J. P., P. Levy, G. Barone-Rochette, **R. Tamisier**, H. Pierre, M. Peeters, J. M. Mallion, and J. L. Pepin. 2008. Masked hypertension in obstructive sleep apnea syndrome. J Hypertens 26(5):885-92.

Faure, P., **R. Tamisier**, J. P. Baguet, A. Favier, S. Halimi, P. Levy, and J. L. Pepin. 2008. Impairment of serum albumin antioxidant properties in obstructive sleep apnoea syndrome. Eur Respir J 31(5):1046-53.

Gilmartin, G. S., **R. Tamisier**, M. Curley, and J. W. Weiss. 2008. Ventilatory, hemodynamic, sympathetic nervous system, and vascular reactivity changes after recurrent nocturnal sustained hypoxia in humans. Am J Physiol Heart Circ Physiol 295(2):H778-85.

Hunt, B. E., **R. Tamisier**, G. S. Gilmartin, M. Curley, A. Anand, and J. W. Weiss. 2008. Baroreflex responsiveness during ventilatory acclimatization in humans. Am J Physiol Heart Circ Physiol 295(4):H1794-801.

Lefebvre, B., J. L. Pepin, J. P. Baguet, **R. Tamisier**, M. Roustit, K. Riedweg, G. Bessard, P. Levy, and F. Stanke-Labesque. 2008. Leukotriene B4: early mediator of atherosclerosis in obstructive sleep apnoea? Eur Respir J 32(1):113-20.

Levy, P., J. L. Pepin, C. Arnaud, **R. Tamisier**, J. C. Borel, M. Dematteis, D. Godin-Ribuot, and C. Ribuot. 2008. Intermittent hypoxia and sleep-disordered breathing: current concepts and perspectives. Eur Respir J 32(4):1082-95.

Pepin, J. L., P. Defaye, E. Vincent, S. Christophle-Boulard, **R. Tamisier**, and P. L. P. 2008. Sleep apnea diagnosis using an ECG Holter device including a nasal pressure (NP) recording: Validation of visual and automatic analysis of nasal pressure versus full polysomnography. Sleep Med.

Laza, F., **R. Tamisier**, B. Wuyam, J. C. Borel, C. Saint Raymond, A. Labarre-Vila, P. Levy, and J. L. Pepin. 2009. [Chronic respiratory failure and Steinert's myotonia.]. Rev Mal Respir 26(1):83-6.

Liu, Y., E. S. Ji, S. Xiang, **R. Tamisier**, J. Tong, J. Huang, and J. W. Weiss. 2009. Exposure to cyclic intermittent hypoxia increases expression of functional NMDA receptors in the rat carotid body. J Appl Physiol 106(1):259-67.

Tamisier, R., G. S. Gilmartin, S. H. Launois, J. L. Pepin, H. Nespoulet, R. J. Thomas, P. Levy, and J. W. Weiss. 2009. A New Model of Chronic Intermittent Hypoxia in Humans: Effect on ventilation, sleep and blood pressure. J Appl Physiol. Epub ahead of print

Pr Dominic PERENNOU

Thématiques de recherche

Posture, équilibre et locomotion.

N° RPPS : 10002184769

Diplômes

- **Docteur en Médecine**. Faculté de Médecine de Montpellier (1990).
- **DES de Médecine Physique et réadaptation (MPR).** Faculté de Médecine de Montpellier (1990).
- **Titulaire de 6 DU et CES** dans les domaines de la santé
- **Maîtrise en biologie humaine** (1992).
- **DEA :** Sciences et techniques appliquées au Handicap (Dijon 1993).
- **Docteur d'Université** (1998).
- **Habilitation à diriger les recherches** (Montpellier 2002).
- **Professeur des Universités - Praticien Hospitalier** (Université de Bourgogne 2003).

Titres & fonctions

- **Internes des hôpitaux de Montpellier** (concours 1985).
- **Chef de Clinique à la Faculté de Médecine de Montpellier et Assistant des Hôpitaux** (1990-1993).
- **Praticien Hospitalier depuis** 1993
- **Responsable de l'Unité de Rééducation Neurologique des CHU Montpellier-Nîmes** (1995-2003).
- **Année à l'étranger à l'Institute of Neurology de Londres** (mai 2000- avril 2001).
- **Professeur à l'Université de Bourgogne, Chef du service de Rééducation Neurologique du CHU de Dijon** et au profit de l'unité INSERM U887 Motricité-plasticité de septembre 2003 à mars 2008.
- **Professeur à l'Université (UJF), Chef de la Clinique de Médecine Physique et Réadaptation du CHU**, équipe Motricité-Plasticité du laboratoire TimC-IMAG UMR UJF-CNRS 5525 (avril 2008 -)

Expérience professionnelle

- **Soins :** 30 ans dans différentes filières de soins en MPR : enfants, adultes et personnes âgées, rhumatologie et ortho-traumatologie, neurologie, amputé et cardiovasculaire. 19 ans de management d'équipe en CHU.
- **Enseignement :** 25 ans en 1er, 2nd et 3ème cycles d’études médicales, dans les cycles de recherche et en formation continue. Animateur du groupe ECN du COFEMER. Production de nombreux documents pédagogiques papiers et électroniques.
- **Recherche :** responsable de 12 projets financés. Co-inventeur du brevet (N°070170435). Auteur de 66 articles indexés (H index= 20 ; IF cumulé pondéré = 130 ; SIGAPS articles classés A 24%, B 13%, C 29%), 5 directions d'ouvrages, 75 chapitres dans des livres (6 en anglais), 109 résumés dans des périodiques à comité de lecture, 12 articles de vulgarisation, 269 communications à des congrès (67 conférences sur invitation). Expert pour de nombreuses revues scientifiques et conseils scientifiques de plusieures Fondations ou Institutions, expert pour l’HAS à plusieurs reprises. Organisation de 7 congrès dont le congrès annuel de la SOFMER en 2005. Membre de nombreuses sociétés savantes, président de

l'association Posture Equilibre locomotion depuis 2006, élu au CA de la SOFMER et du COFEMER, secrétaire scientifique de la SOFMER depuis 2012. Co-responsable du sous-groupe d'intérêt Equilibre de la société mondiale de rééducation neurologique depuis 2012. Editeur associé de la revue Annals of Physical and Rehabilitation Medicine depuis 2012. Editeur en chef au 2 janvier 2014.

Principales publications

(5 dernières années)

1. **Babyar SR, Peterson MG, Bohannon R, Perennou D, and Reding M**. Clinical examination tools for lateropulsion or pusher syndrome following stroke: a systematic review of the literature. *Clin Rehabil* 23: 639-650, 2009.

2. **Barbieri G, Fouque F, Pozzo T, and Perennou D**. A biological walker is faster and better recognized when aligned with body axis observer. *Gait Posture* 38: 981-986.

3. **Barbieri G, Gissot AS, and Perennou D**. Ageing of the postural vertical. *Age (Dordr)* 32: 51-60.

4. **Barra J, Benaim C, Chauvineau V, Ohlmann T, Gresty M, and Perennou D**. Are rotations in perceived visual vertical and body axis after stroke caused by the same mechanism? *Stroke* 39: 3099-3101, 2008.

5. **Barra J, Marquer A, Joassin R, Reymond C, Metge L, Chauvineau V, and Perennou D**. Humans use internal models to construct and update a sense of verticality. *Brain* 133: 3552-3563.

6. **Barra J, Perennou D, Thilo KV, Gresty MA, and Bronstein AM**. The awareness of body orientation modulates the perception of visual vertical. *Neuropsychologia* 50: 2492-2498.

7. **Genthon N, Rougier P, Gissot AS, Froger J, Pelissier J, and Perennou D**. Contribution of each lower limb to upright standing in stroke patients. *Stroke* 39: 1793-1799, 2008.

8. **Gremeaux V, Damak S, Troisgros O, Feki A, Laroche D, Perennou D, Benaim C, and Casillas JM**. Selecting a test for the clinical assessment of balance and walking capacity at the definitive fitting state after unilateral amputation: a comparative study. *Prosthet Orthot Int* 36: 415-422.

9. **Gremeaux V, Iskandar M, Kervio G, Deley G, Perennou D, and Casillas JM**. Comparative analysis of oxygen uptake in elderly subjects performing two walk tests: the six-minute walk test and the 200-m fast walk test. *Clin Rehabil* 22: 162-168, 2008.

10. **Guillebastre B, Rougier PR, Sibille B, Chrispin A, Detante O, and Perennou DA**. When might a cane be necessary for walking following a stroke? *Neurorehabil Neural Repair* 26: 173-177.

11. **Simoneau EM, Billot M, Martin A, Perennou D, and Van Hoecke J**. Difficult memory task during postural tasks of various difficulties in young and

older people: a pilot study. *Clin Neurophysiol* 119: 1158-1165, 2008.

12. **Yelnik AP, Le Breton F, Colle FM, Bonan IV, Hugeron C, Egal V, Lebomin E, Regnaux JP, Perennou D, and Vicaut E**. Rehabilitation of balance after stroke with multisensorial training: a single-blind randomized controlled study. *Neurorehabil Neural Repair* 22: 468-476, 2008.
