# Supplementary material for: Continuous positive airway pressure improves gait control in severe obstructive sleep apnoea: A prospective study
Source: PLoS One. 2018 Feb 23;13(2):e0192442. doi: 10.1371/journal.pone.0192442 (PMC5825012; doi:10.1371/journal.pone.0192442)
Supplement: S2 Protocol — English language summary. (DOCX) [file pone.0192442.s003.docx]

**The brain to effort:**

**Effects of hypoxia on the respiratory-ill patient**

**Running title:** NEUROX

**Summary of the project**

#### The brain to effort: Effects of hypoxia on the respiratory-ill patient

| Study centre: Grenoble Alpes University Hospital |
| --- |
| Principal Investigator: Pr. Patrick LEVY |
| Associated investigators:  Dr. Bernard WUYAM, Pr. Jean-Louis PEPIN, Dr. Renaud TAMISIER, Pr. Dominique PERENNOU.  Scientific coordinators:  Mr Samuel VERGES, Pr. Guillaume MILLET, Mr Mathieu GRUET, Mr Thomas RUPP, Pr. Stéphane PERREY, Mr Sébastien BAILLIEUL. |
| Promotor: C.H.U. de Grenoble. |
| Objectives:   - To study the effects of hypoxia, either chronic or intermittent on cerebral function, in chronically-ill respiratory patients (chronic obstructive pulmonary disease: COPD and obstructive sleep apnoea syndrome: OSAS) compared to healthy matched subjects; - To study cerebral neurophysiological responses at rest and to effort, including cerebral perfusion and oxygenation, cortical excitability and the resulting motor control, as well as non-automatic gait and postural control impairments in hypoxemic (diurnal continuous in COPD patients and nocturnal intermittent in OSAS patients) before and after a treatment aiming at normalizing blood gases abnormalities (nasal O_2_ in COPD patients and continuous positive airway pressure (CPAP) in OSAS patients. |
| Methodology: Prospective, controlled, interventional and physiopathological clinical study |
| Number of subjects: 18 COPD patients, 18 OSAS patients et 36 healthy control subjects |
| Inclusion criteria:  COPD group   - GOLD stage III-IV, MEVS/FVC < 0,7 et MEVS reversibility post bronchodilator < 50% of predicted values - Body mass index (BMI) < 30 kg/m² - 18 ≤ Age ≤ 80 years old - Non-smoker or former smoker (subjects who quit smoking for at least 3 months) - No exacerbation in the prior 3 months - PaCO2 < 45 mmHg at rest, in room air - No overlap syndrome (subject free of OSAS).  OSAS group  - Newly diagnosed OSAS patients (apnoea/hypopnoea index (AHI) >30 events/hour) - Epworth sleepiness scale score >10 - 18 ≤ Age ≤ 80 years old - BMI < 30 kg/m²   **Control subjects group**   - 18 ≤ Age ≤ 80 years old - BMI < 30 kg/m² - Non-smoker - Without chronic respiratory, cardiovascular, metabolic, kidney or neuromuscular diseases. Without neurological, vestibular and/or visual impairment. |
| **Exclusion criteria:**  COPD and OSAS group:   - Chronic cardiovascular, metabolic, kidney or neuromuscular diseases. - OH consumption - BMI > 30 kg/m² - Psychiatric or behavioural disorders; an existing vestibular and/or visual impairment; an existing neurological disorder with gait and/or postural control impairment (Parkinson disease, stroke…) and cognitive impairments - Contraindication to magnetic field appliance.   **Control subjects group**   - Chronic respiratory, cardiovascular, metabolic, kidney or neuromuscular diseases and or neurological, vestibular and/or visual impairment. - OH consumption - BMI > 30 kg/m² - Psychiatric or behavioural disorders; an existing vestibular and/or visual impairment; an existing neurological disorder with gait and/or postural control impairment (Parkinson disease, stroke…) and cognitive impairments - Contraindication to magnetic field appliance. |
| Participation length for a subject: 8 weeks (COPD group) - 14 weeks (OSAS group) - 4 weeks (controls) |
| Total study length : 24 months |
| Primary study endpoint:   - Desoxy-haemoglobin and total haemoglobin quantitative changes at a muscular and cerebral levels measured by functional near-infrared spectroscopy (fNIRS) from rest to effort and in response to different inspired fractions of O_2_ and CO_2_   Secondary study endpoints:   - Motor evoked potential amplitude and silent periods changes in response to transcranial magnetic stimulation (TMS) before and after treatment - Spatiotemporal gait (stride time, step length…) and postural (center of pressure length, surface and speed…) parameters changes in single and dual-task condition - Desoxy-haemoglobin and total haemoglobin quantitative changes at a muscular and cerebral levels measured by functional near-infrared spectroscopy (fNIRS) while walking and standing before and after treatment. |
| Security assessment: Clinical recording of unexpected medical events |
| **Ethical authorization (CPP Sud Est V, Grenoble):** 2012, April 4^th^  **AFSSAPS authorization**: 2012, February 2^nd^  **Clinical Trials registration:**   - **NCT:** NCT02854280 - **URL:** https://clinicaltrials.gov/ct2/results?cond=&term=neurox&cntry1=&state1=&SearchAll=Search+all+studies&recrs= |
