# Supplementary material for: Continuous positive airway pressure improves gait control in severe obstructive sleep apnoea: A prospective study
Source: PLoS One. 2018 Feb 23;13(2):e0192442. doi: 10.1371/journal.pone.0192442 (PMC5825012; doi:10.1371/journal.pone.0192442)
Supplement: S1 File — (PDF) [file pone.0192442.s004.pdf]

COMITE DE PROTECTION DES PERSONNES
Sud Est V

Président :
M. Jean GRUNWALD

Vice-Président :
Dr Daniel ANGLADE

Secrétaire Général :
Dr Dominique CHARLETY

Secrétaire Général Adjoint :
Dr Joel JUGE

Site internet
http://www.cppsudest5.fr

Secrétariat :
cppsudest5@chu-grenoble.fr

F. LUCZAK
N. CESTARO
04 76 76 57 83
04 76 76 51 77

Domiciliation :

CHU de Grenoble
Comité de Protection des Personnes

Adresse postale :
CS 10217
38043 GRENOBLE Cedex 9

Adresse géographique :
R.C. Haut
Hall Vercors
Bd de la Chantourne
38700 LA TRONCHE

N° SIRET : 130 016 017 00019
N°APE : 8412Z

IRB: 6705

Grenoble, le 21/01/2014

Promoteur :
Madame SABBAH-GUILLAUME H.
Directrice de la Recherche Clinique et de l'Innovation
CHU de Grenoble

Réf. CPP : 12-CHUG-12 (Modif. Subst. 14-01)
Réf. Etude : NEUROX
N° ID RCB : 2012-A00158-35 / 1
Réf. AFSSAPS
Documents fournis :
Protocole : V1.2 du 02.12.2013
Lettres d'information au patient BPCO, SAOS et au sujet contrôle : V1.2 du 02.12.2013
Formulaire de consentement : V1.2 du 02.12.2013
Promoteur : CHU de Grenoble
Investigateur principal : Pr. Patrick LEVY - CHU de Grenoble
Type Recherche Biomédicale : AUTRE

CHU de Grenoble
Délégation à la Recherche Clinique
24 JAN. 2014
Pavillon [illegible]

Objet : **Avis – Modification Substantielle**

Madame,

Le Comité a bien reçu le 12/12/2013 les modifications du protocole intitulé :

Le cerveau à l'effort : Effets de l'hypoxie chez le malade respiratoire

pour lequel le Comité avait donné un avis favorable en date du 04/04/2012 et 13/03/2013 (maintien d'avis)

Cette modification substantielle porte sur :

- L'ajout d'un l'investigateur : le Pr Dominique PERENNOU – CHU de Grenoble - N° de RPPS : 10002184769
- Ajout d'évaluations non invasives des dysfonctions cérébrales en lien avec l'exercice des sujets porteurs d'apnées du sommeil, portant sur les fonctions exécutives : ajout d'un examen neuropsychologique et évaluation de la marche et de la posture chez des patients SAOS et des sujets contrôles

Compte tenu des modifications apportées au protocole, et après avis des rapporteurs, cet avis reste **FAVORABLE** en date du **15 JANVIER 2014.**

Les personnes ayant délibéré sur le projet sont :

1er collège :
1 - Recherche biomédicale
Titulaire : Dr ANGLADE Daniel
Suppléants : Dr KUENTZ François
Dr SEIGNEURIN Aranud
2 - Médecin généraliste :
Titulaire : Dr TIRARD Véronique
3 – Pharmacien hospitalier
Titulaire : M. BEDOUCH Pierrick
Suppléant : Mme CHARLETY Dominique
4 – Infirmier :
Titulaire : Mr DESSUS Christophe

2ème collège :
5 - Personnes qualifiées "éthique"
Titulaire : M BOARINI Serge
Suppléant : M RIBUOT Christophe
6 - Psychologue
Titulaire : M. GRUNWALD Jean
7 – Travailleur social
Néant
8 - Personnes qualifiées "juridique"
Titulaire : Mme DUCKI Myriam
9 – Représentants d'association agréée de malades et d'usagers du système de santé
Titulaire : Mme PELLOUX Colette

Aucun membre délibérant du comité n'est affecté par un conflit d'intérêt

Je vous prie de croire, **Madame,** à l'expression de mes salutations distinguées.

Le Président,
Jean GRUNWALD
