# Supplementary figures and images for: Continuous positive airway pressure improves gait control in severe obstructive sleep apnoea: A prospective study
Source: PLoS One. 2018 Feb 23;13(2):e0192442. doi: 10.1371/journal.pone.0192442 (PMC5825012; doi:10.1371/journal.pone.0192442)

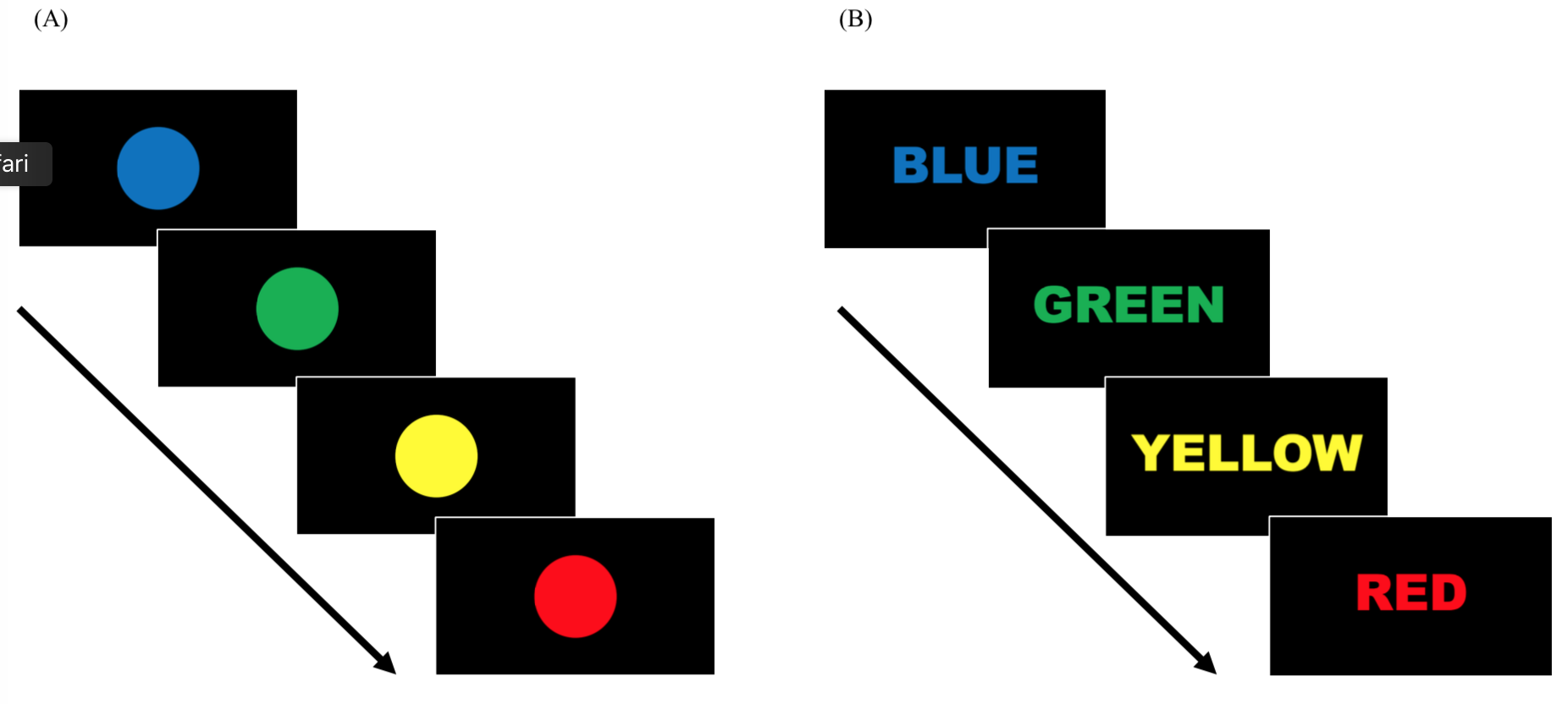

Supplement: S1 Fig — The four colours used in our Stroop test were consecutively displayed on the screen (A), then words written in the congruent font colour were displayed (B). Participants were instructed to give the right colour name (A) and to name the words font colour (B). (TIF) [file pone.0192442.s005.tif]
